# Evidence for the expression of abundant microRNAs in the locust genome

**DOI:** 10.1038/srep13608

**Published:** 2015-09-02

**Authors:** Yanli Wang, Feng Jiang, Huimin Wang, Tianqi Song, Yuanyuan Wei, Meiling Yang, Jianzhen Zhang, Le Kang

**Affiliations:** 1 Institute of Applied Biology, Shanxi University, Taiyuan, Shanxi, China; 2 Beijing Institutes of Life Science, Chinese Academy of Sciences, Beijing, China; 3 Institute of Zoology, Chinese Academy of Sciences, Beijing, China

## Abstract

Substantial accumulation of neutral sequences accounts for genome size expansion in animal genomes. Numerous novel microRNAs (miRNAs), which evolve in a birth and death manner, are considered evolutionary neutral sequences. The migratory locust is an ideal model to determine whether large genomes contain abundant neutral miRNAs because of its large genome size. A total of 833 miRNAs were discovered, and several miRNAs were randomly chosen for validation by Northern blot and RIP-qPCR. Three additional verification methods, namely, processing-dependent methods of miRNA biogenesis using RNAi, evolutionary comparison with closely related species, and evidence supported by tissue-specific expression, were applied to provide compelling results that support the authenticity of locust miRNAs. We observed that abundant local duplication events of miRNAs, which were unique in locusts compared with those in other insects with small genome sizes, may be responsible for the substantial acquisition of miRNAs in locusts. Together, multiple evidence showed that the locust genome experienced a burst of miRNA acquisition, suggesting that genome size expansion may have considerable influences of miRNA innovation. These results provide new insight into the genomic dynamics of miRNA repertoires under genome size evolution.

MicroRNAs (miRNAs) are endogenous ~22-nucleotide (nt) noncoding RNAs that act as post-transcriptional regulators of the stability or translation of mRNA targets^1^. MiRNA biogenesis is a multistep process that requires the coordination of several processing enzymes. In animals, miRNA transcription is initiated by RNA polymerase II in the nucleus. The primary transcripts, which are folded into hairpin molecules, are cleaved by *Drosha* endonuclease to form miRNA precursors^2^. The resulting miRNA precursors are transported from the nucleus to the cytoplasm by Exportin-5 and then further processed by *Dicer* endonuclease to form miRNA duplexes. miRNAs in either the 5′ or 3′ end of their precursors are loaded into an RNA-induced silencing complex, which yields functional miRNAs^3^. The binding of functional miRNAs to target 3′ untranslated regions of target mRNAs within the RNA-induced silencing complex generally results in either degradation of target mRNAs or translational repression^4^. In addition, several miRNAs are known to control gene expression by binding the coding region of target mRNAs^5^,^6^.

The birth and death of an miRNA are the major factors that influence the number of miRNAs in the genome of a given evolutionary lineage^7^. Genomic dynamics of miRNA repertoires have provided an enormous potential to drive evolutionary novelties through expanding gene regulatory networks^8^. Rapid expansions in miRNA repertoires correlate with the major innovations in the evolution of animal complexity^8^. The present status of a miRNA repertoire of any given species is the net effect of the interplay between the birth and death of miRNAs throughout evolution. MiRNA birth may emerge by *de novo* formation of new miRNA-like hairpins in existing transcripts or by duplication and subsequent divergence of pre-existing miRNAs^7^,^9^. MiRNA death may be ascribed to the accumulation of considerable variations that disrupt the formation of miRNA hairpins; therefore, the decayed miRNAs cannot be recognized by miRNA processing endonucleases and are subsequently swept out from the genome^10^. Previous studies on several evolutionary lineages have suggested that the rates of the birth and death of miRNAs considerably vary for different lineages^7^,^11^,^12^,^13^. Therefore, the effects of these processes result in the gain and loss of miRNAs in a species, thereby having profound influences on the genomic dynamics of miRNA repertoires^12^.

The migratory locust, *Locusta migratoria*, is a notorious pest that represents a classical species for studies on the effects of phenotypic plasticity on environmental adaption^14^,^15^. Recently, we reported that miRNA-dependent regulation of gene expression plays an important role in phenotypic transitions in locusts^6^. Compared with other currently sequenced animal genomes, the locust genome has a larger genome size, which is twice the genome size of human^16^. The genome size expansion of locusts is not derived from the two/three rounds of whole genome duplication, which are typical in vertebrate genomes^17^. MiRNA discovery in the locust genome can avoid the possible influence of whole genome duplication on genomic dynamics of miRNA repertoires. Thus, this genome represents an ideal model to accurately study the effects of the increase in genome size on miRNA evolution. However, to date, only seven locust miRNAs have been deposited in miRBase. Moreover, only a single study with limited data, independent of genomic sequences, has been conducted to identify such regulatory genes^18^. A comprehensive study for miRNA discovery has not been performed to determine the precise extent of miRNA repertoires in locusts.

The majority of other miRNA discovery studies only used small RNA sequencing data in miRNA identification. In this study, we utilized combined high-throughput sequencing and validation methods to identify locust miRNAs. MiRNA repertoires were comprehensively analyzed by combining genome sequence searching and small RNA sequencing for multiple developmental stages and multiple tissues to search for evolutionarily conserved and lineage-specific miRNAs, respectively. Our analyses revealed a large number of miRNA repertoires in the locust genome. Three additional validation methods were applied to support the authenticity of the identified miRNAs. These methods were processing-dependent methods of miRNA biogenesis using RNA interference (RNAi), evolutionary comparison with closely related species, and evidence supported by tissue-specific expression. We also found numerous miRNAs that arose from local duplication events. Comparative genomic searches across insect genomes indicated that the abundant local duplication events of miRNAs were unique in the locust genome, compared with other insect genomes with small genome sizes. These results suggested that genome size expansion had a profound effect on genomic dynamics of miRNA repertoires in locusts.

## Results

### Overview of the locust small RNA transcriptomes

We isolated small RNAs from eggs, gregarious and solitarious fourth-instar larvae, and adults of *L. migratoria* for miRNA identification. After size separation and preparation of the libraries, the small RNA libraries were successfully subjected to high-throughput sequencing using Illumina Genome Analyzer IIx sequencing system. The raw sequencing reads were processed through several steps of quality filtering to ensure that they pass the stringent quality criteria. The low quality reads, which represented ~1% of the raw sequencing reads, met the filtering criteria; they contained more than 3Ns, or consisted of simple repeat bases, or were not composed of bases with a minimum Phred score of Q20. After quality filtering for low quality reads and trimming for adaptor sequences, a total of 10,374,480 reads for eggs, 15,812,843 reads for gregarious larvae, 14,293,073 reads for solitarious larvae, and 11,618,874 reads for adults were obtained. The size distribution of the reads is shown in Figure S1. In these libraries, the total read percentages over different read lengths showed that the peak was found at 22 nt, which represents the typical insect miRNA length. Another peak at 27–28 bp could be attributed to Piwi-interacting RNAs, which are commonly detected in insect small RNA libraries^18^. After clustering the sequencing reads based on sequence similarity, 2,088,868, 2,239,067, 2,371,811, and 2,162,721 unique sequences were obtained in the libraries of eggs, gregarious larvae, solitarious larvae, and adults, respectively.

### Discovery and characterization of locust miRNAs

The recently sequenced and assembled *L. migratoria* genome by our group was used to identify miRNAs in locusts using two complementary strategies^16^. First, small RNA transcriptomes were determined by high-throughput sequencing, and the locust miRNAs were identified using the miRDeep package^19^. Second, all known arthropod miRNAs deposited in the latest release of miRBase were used to identify the locust miRNA homologs by homology searches.

In the first approach, all the locust miRNAs discovered were initially identified by the miRDeep package, which uses a probabilistic model of miRNA processing to score the compatibility of the position and frequency of sequenced reads^19^. To improve prediction capacity, sequencing data from multiple tissues were also involved in miRDeep prediction. A total of 779 distinct putative miRNA precursors were identified by the two criteria simultaneously. Sequence similarity searches showed that 2, 12, 1, and 3 of the 779 putative miRNA precursors provided significant hits against tRNAs, rRNAs, snRNAs, and snoRNAs, respectively. Therefore, these putative miRNA precursors were removed from further analysis because they were likely to be other types of non-coding small RNAs. These resulting putative miRNA precursors were further aligned against the locust genome assembly to determine whether they overlap repetitive elements identified by the RepeatMasker program. One miRNA precursor was considered likely to be a transposable element (TE) from a long interspersed element family. Figure S2 shows an example of *L*. *migratoria* miRNA that was identified from high-throughput sequencing. The putative miRNA precursor miR-993, which was 113 nt long, was located at the scaffold16951 of the locust genome assembly along with reads that aligned to this precursor. The discrete alignment manner of the sequencing reads that aligned to the precursor was observed and used to divide the sequencing reads into two categories: 5P and 3P reads, which were close to the 5′ and 3′ ends of the precursor, respectively. The predominant 5P reads, which were approximately fourfold more frequent than the 3P reads, were the mature miRNAs. All putative miRNAs predicted by the miRDeep program were further aligned to all known arthropod miRNAs present in miRBase using BLAST homology searches. A total of 69 distinct putative miRNA precursors of which 5P-miRNAs and/or their corresponding 3P-miRNA showed significant similarity to at least one of the known arthropod miRNAs. These 69 miRNAs were considered the evolutionarily conserved miRNAs. The remaining putative miRNA precursors could not be classified into any of the known miRNA families in miRBase, although they were rated at or above the miRDeep score threshold, and a number of sequencing reads were aligned at the expected location as true 5P/3P mature miRNAs. We also confirmed the presence of consistent 5′ end processing and correct 3′ overhang of mature sequences, and these two consensus criteria were critical in the recognition of novel miRNAs^20^. A total of 760 distinct novel miRNA precursors were identified in the first approach.

In the second approach, we used the *MapMi* annotation system to identify the locust miRNA homologs by determining the validated miRNAs in miRBase to their most likely homologs in the locust genome^21^. This approach has been performed for miRNA identification in a wide range of metazoan genomes^21^,^22^. To provide a conservative estimate in miRNA prediction, only the arthropod miRNAs in miRBase were used as reference. Considering the quality of the mature miRNA alignment and structure of the stem-loop hairpin, we identified 144 distinct putative miRNA precursors that could meet the criterion that a *MapMi* score threshold is more than 35 following the previous studies^22^,^23^. Among these putative miRNA precursors, 71 of 144 were identified by the first approach. For the remaining 73 putative miRNA precursors, we investigated their expression levels in the small RNA libraries. We detected 24 of these putative miRNA precursors with variable expression in at least one small RNA library, which suggested that a number of authentic miRNAs were filtered because of the conservative estimation used in the first approach. Although abundant miRNA precursors were identified in the first approach, the number of miRNA precursors in the locust genome may be underestimated by available sequencing data. For those miRNAs that were not detected in the small RNA libraries, their expression patterns may have been specific to developmental stages that were not sampled in our sequencing samples.

**Figure 1:**
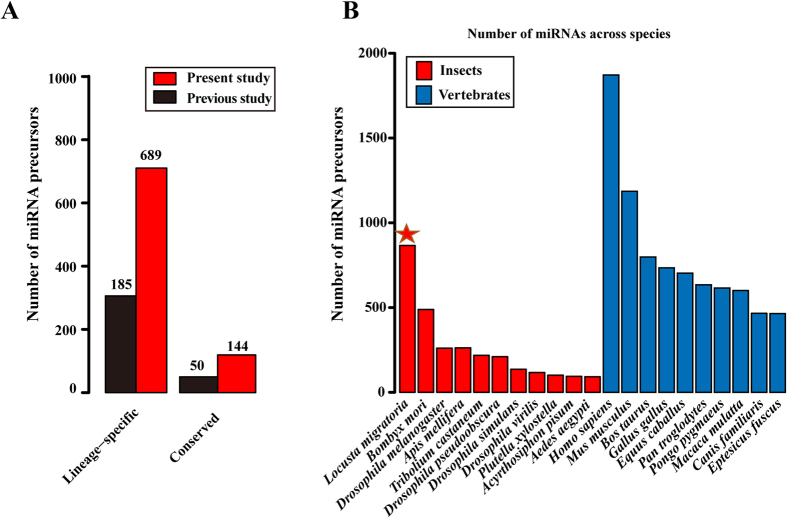
Number of miRNA precursors in the locust genome. (**A**) Summary of identified miRNA precursors in the present study and previous study. (**B**) Summary of identified miRNA precursors from miRBase in metazoan species. The vertebrate and insect miRNA precursors are the predominant representatives (~84%) of metazoan miRNA precursors in miRBase Release 21. The numbers of miRNA precursors at the top ranks are shown in insects and vertebrates.

Independent of the locust genome sequence, 50 conserved miRNA precursors and 185 lineage-specific miRNA precursors (The lineage-specific miRNAs represent the miRNAs showing no homology with insect miRNAs deposited in miRBase in this study) were identified using loop folding methods^18^. All the 50 conserved miRNAs and 136 of 185 lineage-specific miRNAs were detected in the present study (Fig. 1A). We manually checked the 49 lineage-specific miRNAs that were absent in the present study. A large number of these miRNAs (23 in 49) could not be aligned to the locust genome. Seventeen miRNAs that showed multiple genomic hits were related to the locust-specific repetitive elements, which were *de novo* identified by the RepeatModeler program. In addition, nine miRNAs failed to meet the minimum free energy (MFE) criterion of folding, which suggested their self-complementary hairpin origins from random genomic sequences.

Thus, 833 miRNA precursors were identified in the locust genome by combining the results from the two approaches. They included 144 evolutionarily conserved miRNA precursors and 689 lineage-specific miRNA precursors. Six of them were randomly selected for Northern blot validation of miRNA expression (Figure S3). A limited portion (13%, 110 in 833) of the locust miRNAs are located in the intragenic regions, and most of them are intron-derived miRNAs. This suggests that, in comparison to the intergenic regions, the intron size expansion in the locust genome make relatively minor contributions to miRNA innovation. Using the available locust genome, the miRNA precursors identified in the present study were approximately threefold greater than those identified in previous studies, which emphasized the need and crucial importance of the genome sequences in miRNA identification. A summary of these miRNAs is included in Table S1. MiRNAs are usually less abundant in the insect genomes than those in vertebrate genomes^24^. The vertebrate (14,384 in 21,263, ~67%) and insect (3,119 in 21,263, ~15%) miRNA precursors are the predominant representatives (~84%) of metazoan miRNA precursors in miRBase Release 21. Despite considerable sampling and sequencing efforts, the number of miRNAs in an insect genome is generally much less than that in a vertebrate genome (Fig. 1B). The locust genome is an exception because its miRNA precursor number was comparable with that of vertebrate genomes, thereby representing the third highest amount of known metazoan miRNA precursors. These data dramatically extended our comprehension of the characteristics of miRNAs in locusts.

### MiRNA validation using processing-dependent methods of miRNA biogenesis

**Figure 2:**
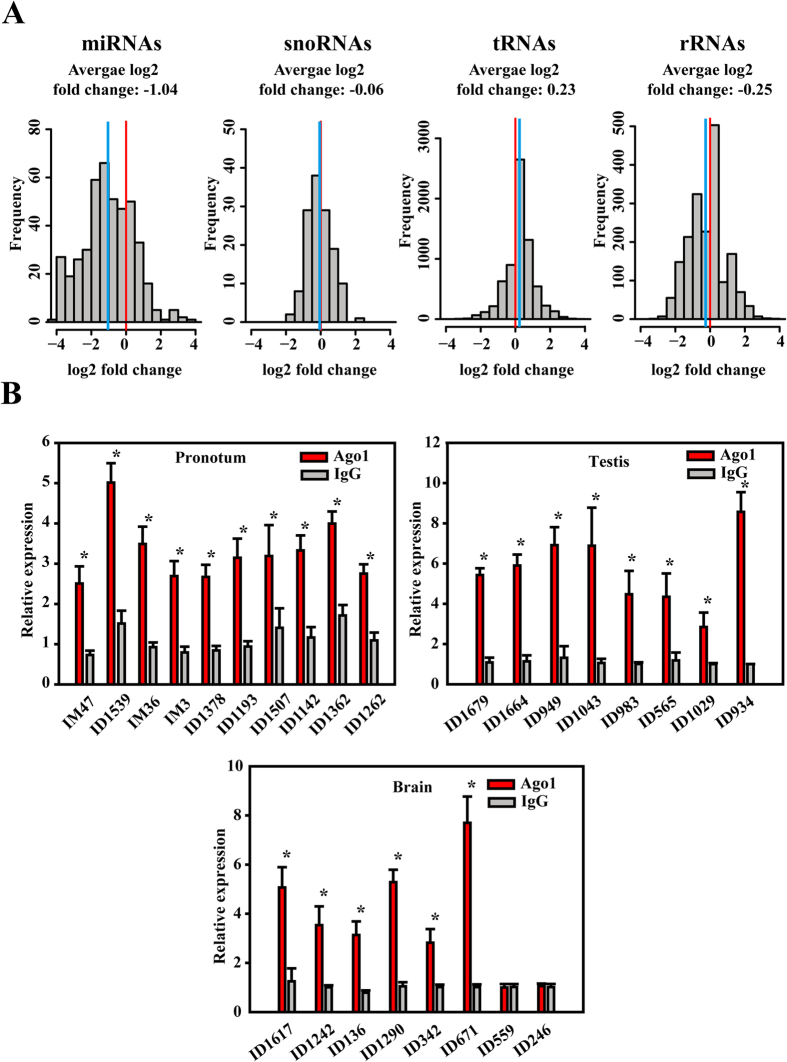
MiRNA validation using processing-dependent methods of miRNA biogenesis. (**A**) Effects of *Drosha* knockdown using RNAi on miRNA biogenesis. MiRNA expression was determined with small RNA sequencing for silenced and negative control tissues. The density distribution of log2 fold changes in the expression for each miRNA between silenced and negative control tissues is shown. (**B**) RIP was performed with an anti-Ago1 antibody, and IgG was used as a negative control. QPCR analysis was performed to amplify miRNAs from the Ago-1 immunoprecipitates from extracts of pronotums, testes and brains. The data for the RIP assay are presented as the mean 6 SEM (n = 6). *indicates P < 0.05.

Numerous locust miRNAs were identified despite the adoption of a conservative threshold. Therefore, we further validated the locust miRNAs using processing-dependent methods of miRNA biogenesis. The RNase III gene family member *Drosha* is the key component of miRNA processing machinery. To determine whether the expression of these miRNAs depends on *Drosha* processing, we knocked down the *Drosha* transcript in pronotums using RNAi (dsRNA), and profiled the small RNA expression by sequencing. We quantized the expression for the small reads that are not frequently cleaved by *Drosha* as negative controls. Specifically, we compared the sequencing reads that mapped to snoRNA, tRNA, and rRNA transcripts in the control and dsRNA pronotums. The mRNA expression of *Drosha* was efficiently reduced (Figure S4, P = 0.011, Student’s *t*-tests), and we observed a good correlation between fold changes determined by miRNA qPCR and small RNA profiling (Figure S5, P = 0.022, R^2^ = 0.441). A total of 440 locust miRNAs were detected in the pronotums. To show that *Drosha* knockdown affects the global level of miRNA expression, we plotted a cumulative distribution of miRNA expression in the control and *Drosha* knockdown pronotums (Figure S6). The global expression of the predicted miRNAs in the *Drosha* knockdown pronotums was significantly lower than those in the control pronotums (P < 0.01, Mann–Whitney–Wilcoxon tests), indicating a global expression reduction induced by *Drosha* knockdown. The average log2 fold change in miRNA expression between the control and dsRNA pronotums was −1.04 upon *Drosha* knockdown, whereas those of snoRNA, tRNA, and rRNA expression were −0.06, 0.23, and −0.25, respectively (Fig. 2A).The expression of miRNAs was significantly reduced, compared with that of snoRNA, tRNA, and rRNA transcripts (P < 0.001, Mann–Whitney–Wilcoxon tests). Argonaute 1 (Ago1) protein plays a role in miRNA biogenesis during miRNA processing^25^. We then investigated whether the locust miRNAs directly interact with Ago1 by RNA-binding protein immunoprecipitation followed by qPCR (RIP-qPCR). Endogenous Ago1 with its miRNA partners was immunoprecipitated using an anti-Ago1 antibody. Ten miRNAs were randomly chosen, and their expression was quantified. As shown in Fig. 2B, all of the locust miRNAs were significantly enriched in Ago1 RIP in pronotums compared with IgG (negative controls). To specifically check the enrichments of lineage-specific miRNAs with low expression, the 16 miRNA were assessed in the testes or brains. A total of 14 (88%, 14 in 16) of them showed highly significant enrichments in Ago1 RIP (Fig. 2B). Overall, these evaluations by processing-dependent methods of miRNA biogenesis showed that the locust miRNAs identified in this study were supported by downregulated expression upon silencing of miRNA biogenesis or interactions with processing proteins in miRNA biogenesis.

### Evidence for the presence of numerous locust miRNAs in an evolutionary view

**Figure 3:**
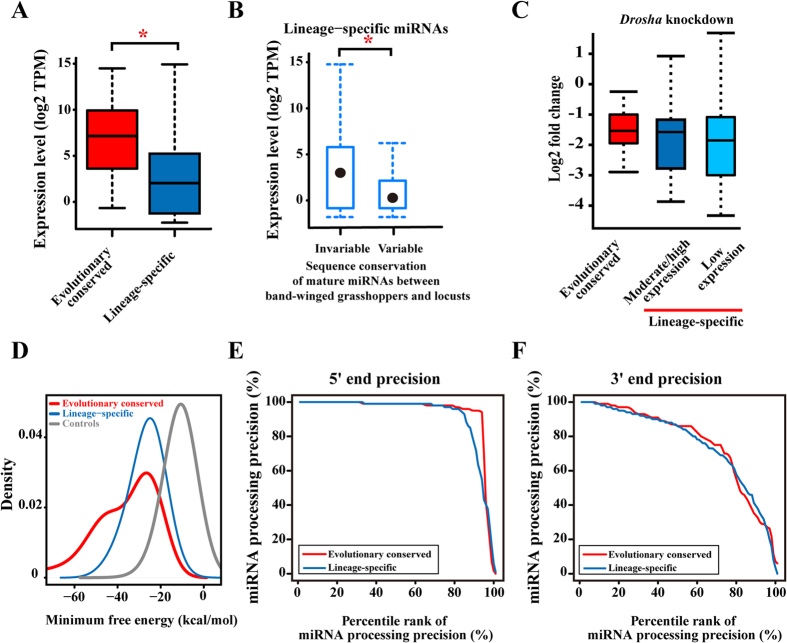
Evidence for the presence of numerous locust miRNAs in an evolutionary view. (**A**) Box plot of miRNA expression for evolutionarily conserved miRNAs and lineage-specific miRNAs. *indicates P < 0.001 (Mann–Whitney–Wilcoxon tests). (**B**) Box plot of lineage-specific miRNA expression for lineage-specific miRNAs that showed no variations and those with sequence variations. The variable group represents the lineage-specific miRNAs in locusts that differed from those in band-winged grasshoppers. *indicates P = 0.009 (Mann–Whitney–Wilcoxon tests). (**C**) Effects of Drosha knockdown using RNAi for miRNAs of the three different categories, namely evolutionarily conserved miRNAs, lineage-specific miRNAs with moderate/high expression and lineage-specific miRNAs with low expression. (**D**) Inferred MFEs of the lineage-specific miRNAs were similar to those of the evolutionarily conserved miRNAs, and significantly stronger than the binding of shuffled control sequences. (**E**,**F**) The miRNA 5′ end and 3′ end processing precision of evolutionarily conserved and lineage-specific miRNAs. The processing precision was calculated as the fraction of mapped reads that corresponded precisely to the consensus sequences of genomic locus. The miRNAs are shown on the *x*-axis and ordered by the processing precision. The miRNA with the most precision is at percentile 1, and the one with the least precision is at percentile 100.

Newly emerged miRNAs in an ancestral lineage are integrated into gene regulatory networks and play important roles in expression regulation. Therefore, their mature sequences should be under strong purifying selection, and they are rarely mutated with secondary loss in the descendants^8^. To check the presence of locust miRNAs in the closely related species sharing common ancestor with *L. migratoria*, we generated 9,483,626 million reads of 18–30 bp from adults of band-winged grasshopper, *Oedaleus asiaticus*^26^. A substantial fraction (46%, 385 in 833) of the locust lineage-specific miRNA precursors (37%, 255 in 689) and evolutionarily conserved miRNA precursors (90%, 130 in 144) were detected in the band-winged grasshopper, which confirmed that they were evolutionarily conserved at least after the emergence of Orthoptera. Similar to a previous report, evolutionarily conserved miRNAs had significantly higher expression levels than the lineage-specific miRNAs (Fig. 3A, P < 0.001, Mann–Whitney–Wilcoxon tests), which implied that the lineage-specific miRNAs were prone to show low expression^27^. Newly emerged miRNAs have been continuously created in metazoan genomes as lineage-specific miRNAs, and these evolutionarily young miRNAs are generally lowly expressed and appear to have nonsubstantial effects on regulatory networks^28^,^29^. Pearson’s correlation tests showed that the expression of the lineage-specific miRNAs in adult locusts had a significantly positive correlation with that of their respective homologs in adult band-winged grasshoppers (P < 0.001, Pearson’s correlation tests). Based on the expression cut-off of TPM > 10, miRNAs were classified into the moderate/high and low expression groups. Numerous homologs (34%, 86 in 255) of the lineage-specific miRNAs exhibit moderate/high expression [transcripts per million (TPM) > 10] in the band-winged grasshoppers. Almost all the homologs of the lineage-specific miRNAs (92%, 79 in 86) that displayed moderate/high expression in band-winged grasshoppers were also found with moderate/high expression in locusts. The substantial expression of these miRNAs implied that these lineage-specific miRNAs might not be lowly expressed genomic by-products, and they have considerable effects on the transcriptome and their target genes in terms of regulatory networks^29^. We examined the strength of natural selection acting on the lineage-specific miRNAs to determine whether the lineage-specific miRNAs with low expression have undergone rapid sequence evolution^28^. Specifically, we determined the sequence variations in the miRNA mature region between band-winged grasshoppers and locusts, and compared the number of variant miRNAs between the lineage-specific miRNAs with moderate/high expression and those with low expression. We excluded the first and last three bases of sequencing reads in sequence variation detection, because of the frequent un-templated modifications at both 5P and 3P ends of mature miRNAs^30^. For the 130 evolutionarily conserved miRNAs detected in band-winged grasshoppers, only one miRNA in the moderate/high expression group had sequence variation in their mature region. This characteristic was consistent with the fact that strong purifying selection intensely constrained evolutionarily conserved miRNAs. However, contrary to that observed in evolutionarily conserved miRNAs (1 in 144), we observed a significant signal of sequence variances for the lineage-specific miRNAs (26 in 255, P < 0.001, χ^2^-tests). We found that the lineage-specific miRNAs that showed no variations were more highly expressed than those with sequence variations (Fig. 3B, P = 0.009, Mann–Whitney–Wilcoxon tests). Based on the expression cut-off of TPM > 10, miRNAs were classified into the moderate/high and low expression groups. Compared with those in the moderate/high expression groups, the lineage-specific miRNAs in the low expression group were subject to a significant signal of sequence variances (3 in 86 of the moderate/high expression group and 23 in 169 of the low expression group; P = 0.025, χ^2^-tests). We repeated the analysis with different cut-offs of TPM (P = 0.010 for TPM > 5 and P = 0.004 for TPM > 15, χ^2^-tests), reaching the same significant signals. Therefore, our analytical results suggested that the numerous lineage-specific miRNAs with moderate/high expression might be under high selective pressures, and showed evolutionary conservation at least after the emergence of Orthoptera. This finding was consistent with the fact that highly expressed miRNAs are under strict selective constraints to maintain sequence uniformity and play critical roles in a broad manner^31^. To determine the silencing effects of *Drosha* knockdown for miRNAs with different evolutionary origin, we compared the expression changes for the evolutionarily conserved miRNAs, the lineage-specific miRNAs with moderate/high expression and with low expression (Fig. 3C). The fold changes between the lineage-specific miRNAs with moderate/high expression and the lineage-specific miRNAs with low expression were comparable (P = 0.681, Mann–Whitney–Wilcoxon tests). Compared with those of the lineage-specific miRNAs with moderate/high expression (P = 0.9347, Mann–Whitney–Wilcoxon tests) and with those of the lineage-specific miRNAs with low expression (P = 0.9815, Mann–Whitney–Wilcoxon tests), the fold changes of evolutionarily conserved miRNAs was not significantly low. These data suggested that, similar to the evolutionarily conserved miRNAs, the lineage-specific miRNAs dependent on *Drosha* processing for their biogenesis, implying the authenticity of the lineage-specific miRNAs.

MiRNA precursors can fold into a stable stem-loop structure, and are processed into mature miRNA with precisely defined 5′ ends and imprecisely defined 3′ ends^32^. This behavior is critical because processing of mature miRNAs from miRNA precursors contributes significantly to mRNA targeting specificity. We compared the fold ability and processing precision of lineage-specific miRNAs with that of evolutionarily conserved miRNAs as positive controls, because evolutionarily conserved miRNAs are more likely to represent authentic miRNAs. Figure 3D shows the distribution of MFE values for structures inferred from the evolutionarily conserved miRNAs, lineage-specific miRNAs, and randomized control genomic sequences flanking the small RNA reads. The lineage-specific miRNAs were predicted to fold into the stem-loop structure with high thermodynamic stability, comparable with the evolutionarily conserved miRNAs, and more strongly than the randomized control sequences. Precise processing of the 5′ ends of mature miRNAs determines miRNA target selection, and imprecise processing of the 3′ ends of mature miRNAs modulates the effectiveness of miRNAs^33^. We found that lineage-specific miRNAs had precisely processed 5′ ends, similar to the evolutionarily conserved miRNAs (Fig. 3E, P = 0.587, Kolmogorov–Smirnov tests). Contrary to the 5′ ends, most of which were imprecisely processed, evolutionarily conserved and lineage-specific miRNAs had abundant mature sequences with the variant 3′ end (Fig. 3F). The lineage-specific miRNAs did not show significant deviation from the evolutionarily conserved miRNAs of imprecisely processed 3′ end (P = 0.705, Kolmogorov–Smirnov tests). Therefore, the processing signatures of lineage-specific miRNAs were comparable with those of evolutionarily conserved miRNAs, which implied that lineage-specific miRNAs were authentic miRNAs processed by *Drosha*.

### Abundant tissue-specific expression of locust lineage-specific miRNAs

**Figure 4:**
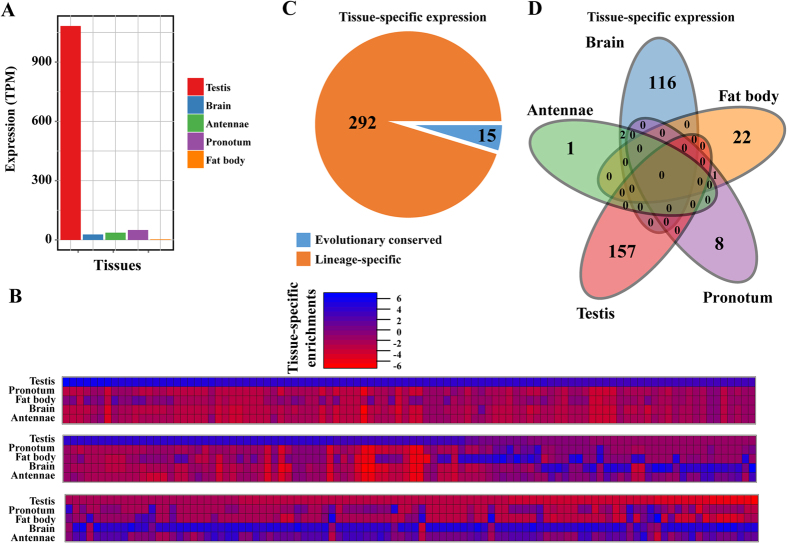
Abundant tissue-specific expression of locust lineage-specific miRNAs. (**A**) ID1685-5P is an example of tissue-specific miRNA, because it shows significantly higher expression in the testes compared with those in other tissues. (**B**) Numerous miRNAs that showed strong specificity in multiply tissues were determined by comparing the expression between the candidate tissue and other condition-specific tissues/developmental stages using statistical differential expression analysis of the edgeR program. (**C**) The majority of miRNAs that showed tissue-specific expression were lineage-specific miRNAs. (**D**) The predominant portion of locust lineage-specific miRNAs showed a tissue-specific manner in the testes or brains.

Given that lineage-specific (new) miRNAs are generally lowly expressed in a tissue-specific manner, especially in the testes or brains, we explored the expression pattern of locust miRNAs by determining tissue specificity^34^,^35^. To determine the spatial expression pattern, we sequenced small RNAs from five tissue samples, namely, the pronotum, testes, antennae, fat bodies, and brains. The tissue-specific expressed miRNAs were identified as they are significantly higher expressed in a given tissue versus its aggregate expression in other samples. For example, ID1685-5P was highly expressed in the testes. The substantial reduction in other non-testis samples showed that the high expression of ID1685-5P was exclusively restricted in the testes (Fig. 4A). We identified numerous miRNAs that showed strong specificity in multiple tissues (Fig. 4B). Approximately 36.9% (307 in 833) miRNAs showed clear tissue bias. These miRNAs were exclusively expressed in a given tissue, but their expression was absent in other tissues or weakly expressed in some other tissues. The homology searches with known miRNAs indicated that the majority (292 in 307, 95%) of the tissue-specific expressed miRNAs were lineage-specific in locusts (Fig. 4C), which represented 42% (292 in 689) of all locust lineage-specific miRNAs. The predominant tissue-specific expressed miRNAs were exclusive in the testes or brains (Fig. 4D). Thus, a large portion of locust lineage-specific miRNAs showed tissue specificity in the testes or brains, which implied the specialized roles of these miRNAs. This result was consistent with the notion that new emergent miRNAs exhibit tissue-specific expression in the testes or brains.

### Numerous miRNAs arise from local duplication events

**Figure 5:**
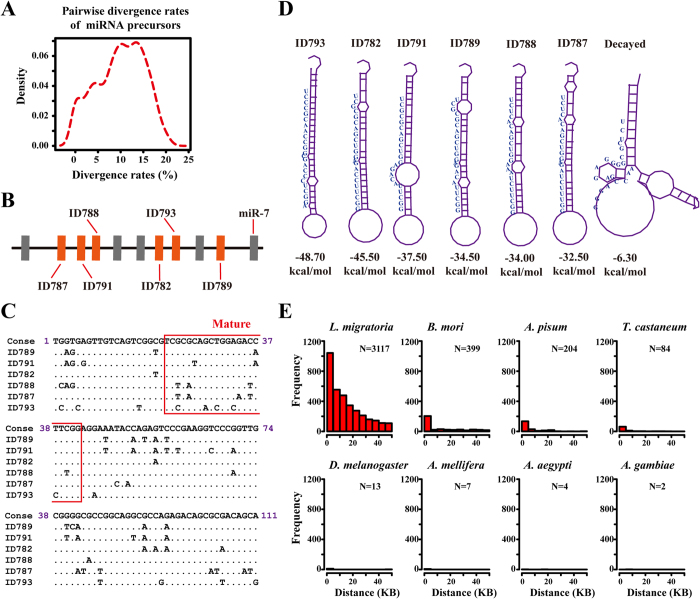
Numerous miRNAs arise from local duplication events. (**A**) A large fraction of miRNA precursors showed sequence similarity to each other. (**B**) Six miRNA precursors that showed sequence similarity were located close to each other in a neighboring region, which suggested that they emerged from local duplication events. (**C**) These six miRNA precursors comprised members of different seed families, although they showed sequence similarity to each other. (**D**) Variations in the six miRNA precursors resulted in fluctuations in the MFEs, thereby influencing the stability of the hairpin structures of miRNA precursors. (**E**) The global–local alignment searches, in which all locust miRNA precursors (termed as original miRNAs) were used, were performed to identify sequences of similarity in their genomic flanking regions (duplication events). The distances between similar copies and original miRNAs were calculated for each miRNA, and the hits for the original miRNA itself were excluded in distance calculation. *L. migratoria*, *Locusta migratoria*; *B. mori*, *Bombyx mori*; *A. pisum*, *Acyrthosiphon pisum*; *T. castaneum*, *Tribolium castaneum*; *D. melanogaster*, *Drosophila melanogaster*; *A. mellifera*, *Apis mellifera*; *A. aegypti*, *Aedes aegypti*; *A. gambiae*, *Anopheles gambiae*.

In the 144 evolutionarily conserved miRNAs, we observed several miRNA duplications that were reported at least in one insect genome in miRBase. These duplications included miR-2, miR-8, miR-9, miR-13, miR-92, miR-193, miR-210, miR-252, miR-263, miR-279, miR-927, miR-2723, and miR-3015. We also detected numerous miRNA duplicates that were unique in locusts, including miR-79, miR-137, miR-184, miR-275, miR-305, miR-306, miR-932, miR-994, miR-998, miR-2788, miR-3879, miR-3930, miR-4957, miR-4960, and miR-9544. Therefore, 65 of 144 (45%) evolutionarily conserved miRNAs were potentially related to duplication events. Duplication is considered to be the major cause for novel miRNA emergence^9^. We performed BLAST searches against themselves of all miRNA miRNA precursors to detect miRNA duplication events, and pairwise sequence searches of miRNA precursors were extracted. The duplication events were identified based on the significant hits, which must meet a minimum cutoff of E-value < 1E-5 and over 50% length coverage of miRNA precursors. With the use of BLASTN searches, we observed a large fraction of miRNA precursors (54%, 480 in 833) that showed sequence similarity to each other. The density distribution of the divergence rates (the percentage of mismatched bases in the miRNA precursors) showed a relatively broad peak centered at 13% (Fig. 5A).This finding suggested that miRNA precursors underwent fast evolution after duplication. BLASTN searches are more prone to align the most similar regions of two miRNA duplicates in a local alignment. Therefore, the divergence rates among miRNA duplicates were underestimated. An example of miRNA duplication is shown in Fig. 5B. Eleven miRNAs were tightly clustered in tandem, and six of them, namely, ID787, ID791, ID788, ID782, ID793, and ID789, shared sequence similarity to each other. The sequence alignments of miRNA precursors showed that the 111-nt miRNA precursor contained 46 variable sites, 11 of which were located at the mature region (Fig. 5C). This result indicated that rapid sequence evolution possibly occurred after duplication of pre-existing miRNAs. Given that the 7–8 bp in the 5′ end of miRNAs (the so-called seed region) are critical for the recognition of miRNA targets, the four distinct seed patterns in these six miRNA paralogs resulted in changes in the complementary interaction between miRNA and targets. The distinguishing feature of miRNAs is the stable folding of their miRNA precursors into proper hairpin structures that are cleaved by the enzyme *Dicer* to yield mature miRNAs^36^. We assessed the stability of the hairpin structures by calculating the MFE using the RNAfold program^37^. The variations in the six miRNA precursors resulted in the remarkable fluctuations of MFE from −32.50 kcal/mol to −48.7 kcal/mol (Fig. 5D), which indicated the influence of rapid miRNA sequence evolution on accurate hairpin structures. The birth and death evolution of miRNAs results in the pseudogenization of active miRNAs in a species-specific manner^38^. In the adjacent genomic region, we also found a decayed miRNA precursor, which might be an ancient copy that suffered from intense sequence variations. Its putative mature sequence was not in the stem portion of the hairpin structure, and showed 64% sequence similarity to the consensus sequence of these six miRNA paralogs (inferred by maximum parsimony methods). The MFE for this decayed miRNA precursor was −6.30 kcal/mol, and it could not be folded into a typical hairpin structure of the miRNA precursor (Fig. 5D). Therefore, the alternative secondary structure could not be subject to *Dicer*-dependent processing, which implied that rapid birth and death processing of miRNAs determined the fate of local duplicates of paralogous miRNAs^34^.

To systematically detect recent local duplication events for both active and decayed miRNAs, we performed global–local alignment searches using all locust miRNA precursors (termed as original miRNAs) to identify sequences of similarity in their genomic flanking regions. The distances between similar copies and original miRNAs were calculated for each miRNA, and the hits for the original miRNA were excluded in distance calculation. For comparison, we also repeated the same analysis for insect representatives whose genomes and miRNA transcriptomes were sequenced. We found abundant similar copies around the original miRNAs at intervals between 0 and 50 kb, with the most frequent interval being <5 kb followed by 5–10 kb (Fig. 5E). As the distance increased, the number of similar copies decreased along the genome, which suggested that tandem duplication of locust miRNAs occurred in a short distance from the original miRNA. Similar copies were scarce around original miRNAs for the other seven insect genomes, the miRNAs of which were less abundant than those of locusts. The total number of similar copies in locusts was at least an order of magnitude greater than those of other insects. Most similar copies in other insects were located from 0 kb to 10 kb, which indicated that the distant similar copies undergoing substantial variations may be progressively removed from these insect genomes under natural selection. Therefore, compared with the other insect genomes, the locust genome could endure numerous paralogous copies that emerged from local duplication, which is a frequent mechanism for miRNA expansion in locusts.

## Discussion

In this study, we performed comprehensive analyses for miRNA discovery and identified 833 miRNAs from the migratory locust *L. migratoria*, an important member of Orthopteran insects with large genome size in animals. Among the identified miRNAs, 144 were evolutionarily conserved to known miRNAs, and 689 were lineage-specific miRNAs. Several of these miRNAs were randomly chosen for validation by Northern blot validation and anti-Ago1 RNA-binding protein immunoprecipitation followed by qPCR. To further verify the miRNAs identified in this study, we provided three additional methods that were dependent on miRNA biogenesis, evolutionary conservation of miRNA characteristics, and tissue-specific expression pattern. These methods confirmed that a large amount of miRNAs emerged during locust evolution. Comparative genomic analyses showed that the expansion of miRNA repertoires could be largely ascribed to extensive local duplication events. This phenomenon was apparently unique in the locust genome compared with other insect genomes with small genome sizes.

The number of putative miRNAs in a given genome could be influenced by the parameters adopted in computational prediction. In the present study, we attempted to make conservative estimates to avoid overestimating the miRNA number in the locust genome using the most stringent cut-off of miRDeep score. Although a high number of miRNAs in the conservative estimates had been identified, we inferred that the locust genome underwent a burst of miRNA emergence. However, in addition to the adoption of a stringent cut-off, more lines of evidence from experimental data and comparative bioinformatics analyses are required to further verify the reliability of bioinformatics prediction. Considering that we used conservative estimates, the number of miRNAs in the locust genome might be underestimated. Combined with sophisticated experimental design, further study for sequencing of more tissues at greater depth is required to discern the extent of miRNA expansion in the locust genome, because it provides new insights into the effects of genome size on miRNA innovation.

Previous studies have reported that TEs can be the source of novel miRNA emergence^39^. Enrichments of TEs in a specific genomic region might facilitate rearrangement by non-allelic homologous recombination, thereby leading to the formation of local duplication of miRNAs^38^. At least 65% of the locust genome were identified as TEs, which demonstrated that TEs significantly contributed to locust genome evolution^16^. Many TEs were detected in the locust transcriptome, and some of these TEs have recently been found to be active or deprived of retrotransposition activities^40^. This phenomenon raises the possibility that many locust-specific miRNAs may be derived from the activities of TEs. In this study, for a conservative estimation, sequencing reads with hits to multiple genomic regions derived from TEs were removed in further miRNA prediction. In addition, the miRNA precursors that overlapped with TEs were also filtered out. Therefore, the actual number of locust miRNAs was likely to be underestimated. We observed several cases in which the expression of sequencing reads related to TEs was silenced in the brain sample with *Drosha* RNAi treatment (data not shown). Given the random expression changes in *Drosha*-independent reads, these TE-derived fragments may not be conclusively considered as authentic miRNAs. With more small RNA and genome data becoming available in the future, a specific study may better address the issue of how many TE-derived miRNAs there are in the locust genome.

Insect genomes are highly dynamic and span three orders of magnitude in size, with very small genomes in Diptera, as well as very large genomes in Orthoptera (see Animal Genome Size Database, www.genomesize.com). A positive correlation between TEs and genome size was observed, although the TE content might be underestimated in large genomes because of the failure to detect diverged or degraded TEs^41^,^42^. The low rate of turnover of TE neutral elements, in which the slow loss of neutral inserts could not offset the new insertion of TE copies, accounted for the accumulation of abundant TEs, thereby leading to increased genome size^43^,^44^,^45^. Up to 60% of the locust genome sequence was dominated by diverse TEs. DNA loss rates measured by neutral TE copies in locusts were lower than those of other insects with small genomes, which suggested that the locust genome was more inclined to tolerate neutral elements^16^. This phenomenon was similar to a case with neutral nuclear elements of mitochondrial genes, which were also slowly lost in *Podisma pedestris*, an orthopteran species with a large genome^45^. The insect genomes sequenced so far are considerably compact compared with vertebrate genomes, except for the *L. migratoria* genome. The *L. migratoria* genome consists of approximately 6.5 Gb, which is over 50 times as large as that of *Drosophila melanogaster*^16^. The vast majority of the genome is transcribed as low expression, a phenomenon known as pervasive transcription^46^,^47^. The pervasive transcription of large genomes might lead to the formation of more miRNA-like hairpins, and then more potential miRNAs^8^. The locust genome has more miRNAs than insects with small genomes, which may be largely ascribed to the expansion of lineage-specific miRNAs. These miRNAs tended to be lowly expressed, which implied that they might be raised by pervasive transcription and represent neutral elements^29^. This finding was consistent with our comparison of miRNA duplication across insect genomes, which suggested that, similar to TE-derived neutral elements, miRNA-derived neutral elements could not be rapidly lost from large genomes. Hence, our data suggested that large genomes demonstrated a propensity to yield more abundant miRNA repertoires, and pervasive transcription and low loss rates of miRNA duplicates might contribute to the creation of abundant miRNA repertoires.

## Materials and Methods

### Insect Rearing

The migratory locusts used in this study were obtained from colonies reared at the Institute of Zoology, Chinese Academy of Sciences, Beijing, China. Solitarious locusts were cultured alone in white metal boxes (10 × 10 × 25 cm^3^) supplied with charcoal-filtered compressed air. Gregarious locusts were reared in large plastic boxes (40 × 40 × 40 cm^3^) at densities of 500–1000 individuals per container. Gregarious and solitarious colonies were reared under a 14 h/10 h light/dark cycle at 30 ± 2 °C and on a diet of fresh greenhouse-grown wheat seedlings and wheat bran^48^.

### Small RNA isolation and deep sequencing

Total RNA was extracted from each sample using TRIzol (Invitrogen) and treated with DNase I following the manufacturer’s instructions. The RNA concentration and purity were measured in an Agilent 2100 Bioanalyzer (Agilent) to verify RNA integrity. Small RNA libraries were constructed using TruSeq small RNA sample preparation kit (Illumina) following the manufacturer’s protocol. The protocol was designed based on miRNA structure properties, i.e., most mature miRNAs have a 5′-phosphate and 3′-hydroxyl group. In brief, the RNA 3′ and RNA 5′ RNA adapters were ligated to their corresponding ends of RNA molecules. Following 5′- and 3′-adapter ligation, the ligated RNA fragments were reverse transcribed using M-MLV reverse transcriptase (Invitrogen). The resulting cDNAs were PCR-amplified with two primers that were complementary to the ends of the adapter sequences. After PCR amplification, the samples were separated by size in a 6% Novex polyacrylamide gel to enrich for miRNA molecules, and sequenced on an Illumina Genome Analyzer IIx sequencing system. The raw sequencing data were deposited at the National Center for Biotechnology Information (Accession number SRP062155).

### Discovery of *L. migratoria* miRNAs

The miRNAs were predicted along the locust genome using sequencing-based and homology approaches. In the sequencing-based approach, the *L. migratoria* genome sequence (GenBank accession number: AVCP000000000) was used as a reference genome. The small RNA sequencing data from eggs, fourth-instar larvae, and adults, and five tissue samples, namely, the pronotum, testes, antennae, fat bodies, and brains, were involved in the miRNA identification. The miRNA identification analyses were independently performed in each sample to allow for authentic detection of miRNAs. The raw sequencing reads from each sample were transformed from the image data, and the quality was assessed. Consequently, the low quality reads and reads with copy number less than 3 were removed from further analysis. In addition, the reads, which showed sequence similarity to adaptor sequences at the start or end terminals, were also filtered using Cutadapt software (https://code.google.com/p/cutadapt/). The high-quality sequencing reads were used as the input data. The miRNA identification analyses were performed against the *L. migratoria* reference genome using the miRDeep (version 2.0.0.5) software package, which depends on the properties of the secondary structure of miRNA precursors^19^. A total miRDeep score was designated based on an algorithm incorporating the statistics of read positions, read frequencies within stem-loops, and posterior probability that the stem-loop was derived from an authentic miRNA. To prevent false positive detection of miRNA stem-loops, the signal-to-noise ratios estimated over 100 rounds of independent permutations were calculated for different miRDeep log-odds score cut-offs. For a conservative prediction, a high stringent criterion of miRDeep score of 10 was adopted as a cut-off point. Therefore, all precursors with total miRDeep scores above the cut-off point were considered as putative miRNAs. The putative miRNAs were removed as degradation fragments if they fell within the protein coding sequences or were classified as other classes of non-coding RNAs, such as tRNAs, rRNAs, snRNAs, and snoRNAs. The repetitive elements were identified using a combination of RepeatModeler (version 1.0.7) and RepeatMasker (version 4.0.5) with cross-match as a search engine^49^. The putative miRNAs mapped to repetitive elements were discarded before further analysis. A putative miRNA that showed a significant BLAST hit (no base mismatches in seed region and no more than two adjacent mismatches elsewhere) against miRBase Release 21 was accepted as an authentic miRNA, and it was annotated as the *L. migratoria* ortholog of the corresponding best-hit miRNA in miRBase^20^. The putative miRNAs identified by the miRDeep program that did not show a significant match to miRBase were regarded as novel miRNAs.

In the homology approach, the *MapMi* program (version 1.5.0) was used to identify the *L. migratoria* miRNA homologs by mapping all the metazoan miRNAs in miRBase against the *L. migratoria* genome^21^. This approach has been implemented recently in a large-scale analysis of microRNA evolution across metazoan species^22^. It was also used to characterize the miRNA repertoire of the red flour beetle *Tribolium castaneum*, an insect with an established genome sequence^50^. In brief, the metazoan miRNAs in miRBase were aligned to the *L. migratoria* genome by Bowtie^51^. Both upstream and downstream regions of each genome alignment were retrieved to produce a pair of potential miRNA stem-loops through extension of 110 nt. Hairpin base-paired structures of each potential miRNA stem-loop were predicted by the RNAfold program from the ViennaRNA package^37^. The putative miRNAs were selected based on the *MapMi* score, which considers both the quality of the genome alignment and hairpin structure of the stem-loops. Following the previous studies, a *MapMi* score threshold of 35 was used according to an empirical analysis of authentic and permutated miRNAs^21^,^22^. Consistent with the sequencing-based approach, we discarded the putative miRNAs showing significant hits with the protein coding sequences or repetitive elements or other classes of non-coding RNAs.

### Evidences to support authenticity of the predicted miRNAs

Templates for *in vitro* transcription reactions were prepared by PCR amplification from plasmid DNA of the cDNA clone of *Drosha* dsRNAs, which were synthesized using T7 RiboMAX Express RNAi System (Promega) following the manufacturer’s instructions. The 2-day old fourth instar nymphs were used for injection experiments. We injected 10 μg of *Drosha* dsRNAs into the abdomen between the second and third abdominal segments using a manual microinjector. Control nymphs were injected with equivalent volumes of Green fluorescent protein dsRNAs. The impacts of miRNA expression after RNAi treatments were assessed for the pronotums of the 5-day old fourth instar nymphs.

To check the presence of locust miRNAs in the closely related species sharing common ancestor with *L. migratoria*, we generated small RNA libraries from adults of band-winged grasshopper, *Oedaleus asiaticus*. We aligned the *O. asiaticus* reads to the locust miRNA precursors with a tolerance of up to two mismatches using Bowtie alignment program^52^. Variations for an alignment were identified using SAMtools/BCFtools programs^53^.

To assess the stability of the structure of the miRNA precursors, the MFE of the miRNA precursors was determined by maximizing the number of favorable base-pairing interactions using RNAfold program from the ViennaRNA package^37^. The processing precision of the 5′ and 3′ ends was calculated as the fraction of mapped reads that corresponded precisely to the consensus sequences of genomic locus. The miRNAs are shown on the *x*-axis and ordered by the processing precision. The miRNA with the most precision is at percentile 1, and the one with the least precision is at percentile 100.

The miRNA expression for each sample was determined by a subordinate program of the miRDeep software package. Following the methods in a previous study, we compared the miRNA expression from five tissues with those from whole animal data at different developmental stages, namely, embryo, larva, and adult^35^. We utilized the edgeR package implemented in the Bioconductor R package to identify tissue-specific expressed miRNAs, which were significantly higher expressed in a given tissue versus its aggregate expression in other samples^54^.

### miRNA duplication event detection

To systematically detect recent local duplication events, we performed global–local alignment searches using all locust miRNA precursors to identify sequences of similarity in their genomic flanking regions. The genomic flanking region for each miRNA precursor was extracted using custom Perl scripts. The miRNA precursor was aligned to its genomic flanking sequences using the GLSEARCH program in the FASTA software package.

### Assays of quantitative PCR for mRNA and miRNA

Total RNAs, including mRNAs and small RNAs, were isolated using the mirVana miRNA Isolation Kit (Life Technologies). M-MLV Reverse Transcriptase (Promega) and miRNA First-Strand cDNA Synthesis Kit (Life Technologies) were used to prepare the OligoDT-primed cDNAs and stem-loop miRNA cDNAs, respectively. Quantitative PCRs of mRNAs and miRNAs were performed using the SYBR Green gene expression and miRNA expression assays, respectively (Tiangen), on a LightCycler® 480 instrument (Roche). Relative expression was determined using the 2^−ΔΔCt^ method of relative quantification. Dissociation curves were verified to confirm the unique amplification of PCR products.

### RNA-binding protein immunoprecipitation

In RNA-binding protein immunoprecipitation experiments, tissues were dissected and homogenized in immunoprecipitation lysis buffer. Each lysate was divided into two samples for anti-Ago1 and anti-IgG (negative controls). The quality and specificity of Ago1 antibody have been validated in our previous study^6^. RNA-binding protein immunoprecipitation was performed using Magna RIP RNA-Binding Protein Immunoprecipitation Kit (Millipore). The amounts of locust miRNAs in the immunoprecipitations were measured as relative enrichments in Ago1 treatments compared with IgG controls.

## Additional Information

**How to cite this article**: Wang, Y. *et al*. Evidence for the expression of abundant microRNAs in the locust genome. *Sci. Rep*. **5**, 13608; doi: 10.1038/srep13608 (2015).

## Supplementary Material

Supplementary Information

## References

[b1] Carthew R. W. & Sontheimer E. J. Origins and Mechanisms of miRNAs and siRNAs. Cell 136, 642–655, 10.1016/j.cell.2009.01.035 (2009). 19239886 PMC2675692

[b2] Pasquinelli A. E. MicroRNAs and their targets: recognition, regulation and an emerging reciprocal relationship. Nat Rev Genet 13, 271–282, 10.1038/nrg3162 (2012). 22411466

[b3] Ameres S. L. & Zamore P. D. Diversifying microRNA sequence and function. Nat Rev Mol Cell Biol 14, 475–488, 10.1038/nrm3611 (2013). 23800994

[b4] Chekulaeva M. & Filipowicz W. Mechanisms of miRNA-mediated post-transcriptional regulation in animal cells. Current opinion in cell biology 21, 452–460, 10.1016/j.ceb.2009.04.009 (2009). 19450959

[b5] Forman J. J., Legesse-Miller A. & Coller H. A. A search for conserved sequences in coding regions reveals that the let-7 microRNA targets Dicer within its coding sequence. Proc Natl Acad Sci USA 105, 14879–14884, 10.1073/pnas.0803230105 (2008). 18812516 PMC2567461

[b6] Yang M. . MicroRNA-133 inhibits behavioral aggregation by controlling dopamine synthesis in locusts. PLoS Genet 10, e1004206, 10.1371/journal.pgen.1004206 (2014). 24586212 PMC3937255

[b7] Meunier J. . Birth and expression evolution of mammalian microRNA genes. Genome Res 23, 34–45, 10.1101/gr.140269.112 (2013). 23034410 PMC3530682

[b8] Heimberg A. M., Sempere L. F., Moy V. N., Donoghue P. C. & Peterson K. J. MicroRNAs and the advent of vertebrate morphological complexity. Proc Natl Acad Sci USA 105, 2946–2950, 10.1073/pnas.0712259105 (2008). 18287013 PMC2268565

[b9] Marco A., Ninova M., Ronshaugen M. & Griffiths-Jones S. Clusters of microRNAs emerge by new hairpins in existing transcripts. Nucleic Acids Res 41, 7745–7752, 10.1093/nar/gkt534 (2013). 23775791 PMC3763532

[b10] Quah S., Hui J. H. & Holland P. W. A Burst of miRNA Innovation in the Early Evolution of Butterflies and Moths. Mol Biol Evol 32, 1161–1174, 10.1093/molbev/msv004 (2015). 25576364 PMC4408404

[b11] Nozawa M., Miura S. & Nei M. Origins and evolution of microRNA genes in Drosophila species. Genome Biol Evol 2, 180–189, 10.1093/gbe/evq009 (2010). 20624724 PMC2942034

[b12] Lu J. . The birth and death of microRNA genes in Drosophila. Nat Genet 40, 351–355, 10.1038/ng.73 (2008). 18278047

[b13] Fromm B., Worren M. M., Hahn C., Hovig E. & Bachmann L. Substantial loss of conserved and gain of novel MicroRNA families in flatworms. Mol Biol Evol 30, 2619–2628, 10.1093/molbev/mst155 (2013). 24025793 PMC3840308

[b14] Wang X. & Kang L. Molecular mechanisms of phase change in locusts. Annu Rev Entomol 59, 225–244, 10.1146/annurev-ento-011613-162019 (2014). 24160426

[b15] Pener M. P. & Simpson S. J. Locust Phase Polyphenism: An Update. Adv Insect Physiol 36, 1–272, 10.1016/S0065-2806(08)36001-9 (2009).

[b16] Wang X. . The locust genome provides insight into swarm formation and long-distance flight. Nat Commun 5, 2957, 10.1038/ncomms3957 (2014). 24423660 PMC3896762

[b17] Dehal P. & Boore J. L. Two rounds of whole genome duplication in the ancestral vertebrate. PLoS Biol 3, e314, 10.1371/journal.pbio.0030314 (2005). 16128622 PMC1197285

[b18] Wei Y., Chen S., Yang P., Ma Z. & Kang L. Characterization and comparative profiling of the small RNA transcriptomes in two phases of locust. Genome Biol 10, R6, 10.1186/gb-2009-10-1-r6 (2009). 19146710 PMC2687794

[b19] Friedlander M. R., Mackowiak S. D., Li N., Chen W. & Rajewsky N. miRDeep2 accurately identifies known and hundreds of novel microRNA genes in seven animal clades. Nucleic Acids Res 40, 37–52, 10.1093/nar/gkr688 (2012). 21911355 PMC3245920

[b20] Kozomara A. & Griffiths-Jones S. miRBase: integrating microRNA annotation and deep-sequencing data. Nucleic Acids Res 39, D152–157, 10.1093/nar/gkq1027 (2011). 21037258 PMC3013655

[b21] Guerra-Assuncao J. A. & Enright A. J. MapMi: automated mapping of microRNA loci. BMC Bioinformatics 11, 133, 10.1186/1471-2105-11-133 (2010). 20233390 PMC2858034

[b22] Guerra-Assuncao J. A. & Enright A. J. Large-scale analysis of microRNA evolution. BMC Genomics 13, 218, 10.1186/1471-2164-13-218 (2012). 22672736 PMC3497579

[b23] Gu J. . miRNA genes of an invasive vector mosquito, Aedes albopictus. PLoS One 8, e67638, 10.1371/journal.pone.0067638 (2013). 23840875 PMC3698096

[b24] Sempere L. F., Cole C. N., McPeek M. A. & Peterson K. J. The phylogenetic distribution of metazoan microRNAs: insights into evolutionary complexity and constraint. Journal of experimental zoology. Part B, Molecular and developmental evolution 306, 575–588, 10.1002/jez.b.21118 (2006). 16838302

[b25] Azzam G., Smibert P., Lai E. C. & Liu J. L. Drosophila Argonaute 1 and its miRNA biogenesis partners are required for oocyte formation and germline cell division. Dev Biol 365, 384–394, 10.1016/j.ydbio.2012.03.005 (2012). 22445511 PMC3763516

[b26] Ma C. . Mitochondrial genomes reveal the global phylogeography and dispersal routes of the migratory locust. Mol Ecol 21, 4344–4358, 10.1111/j.1365-294X.2012.05684.x (2012). 22738353

[b27] Chiang H. R. . Mammalian microRNAs: experimental evaluation of novel and previously annotated genes. Genes Dev 24, 992–1009, 10.1101/gad.1884710 (2010). 20413612 PMC2867214

[b28] Liang H. & Li W. H. Lowly expressed human microRNA genes evolve rapidly. Mol Biol Evol 26, 1195–1198, 10.1093/molbev/msp053 (2009). 19299536 PMC2727378

[b29] Friedländer M. R. . Evidence for the biogenesis of more than 1,000 novel human microRNAs. Genome Biol 15, R57 (2014). 24708865 10.1186/gb-2014-15-4-r57 PMC4054668

[b30] Berezikov E. . Deep annotation of Drosophila melanogaster microRNAs yields insights into their processing, modification, and emergence. Genome Res 21, 203–215, 10.1101/gr.116657.110 (2011). 21177969 PMC3032924

[b31] Romao J. M., Jin W., He M., McAllister T. & le Guan, L. MicroRNAs in bovine adipogenesis: genomic context, expression and function. BMC Genomics 15, 137, 10.1186/1471-2164-15-137 (2014). 24548287 PMC3930007

[b32] Bonnet E., Wuyts J., Rouze P. & Van de Peer Y. Evidence that microRNA precursors, unlike other non-coding RNAs, have lower folding free energies than random sequences. Bioinformatics 20, 2911–2917, 10.1093/bioinformatics/bth374 (2004). 15217813

[b33] Burroughs A. M. . A comprehensive survey of 3’ animal miRNA modification events and a possible role for 3’ adenylation in modulating miRNA targeting effectiveness. Genome Res 20, 1398–1410, 10.1101/gr.106054.110 (2010). 20719920 PMC2945189

[b34] Lyu Y. . New microRNAs in Drosophila–birth, death and cycles of adaptive evolution. PLoS Genet 10, e1004096, 10.1371/journal.pgen.1004096 (2014). 24465220 PMC3900394

[b35] Mohammed J. . Adaptive evolution of testis-specific, recently evolved, clustered miRNAs in Drosophila. RNA 20, 1195–1209, 10.1261/rna.044644.114 (2014). 24942624 PMC4105746

[b36] Berezikov E. Evolution of microRNA diversity and regulation in animals. Nat Rev Genet 12, 846–860, 10.1038/nrg3079 (2011). 22094948

[b37] Lorenz R. . ViennaRNA Package 2.0. Algorithms Mol Biol 6, 26, 10.1186/1748-7188-6-26 (2011). 22115189 PMC3319429

[b38] Zhang R., Wang Y. Q. & Su B. Molecular evolution of a primate-specific microRNA family. Mol Biol Evol 25, 1493–1502, 10.1093/molbev/msn094 (2008). 18417486

[b39] Piriyapongsa J., Marino-Ramirez L. & Jordan I. K. Origin and evolution of human microRNAs from transposable elements. Genetics 176, 1323–1337, 10.1534/genetics.107.072553 (2007). 17435244 PMC1894593

[b40] Jiang F., Yang M., Guo W., Wang X. & Kang L. Large-scale transcriptome analysis of retroelements in the migratory locust, Locusta migratoria. PLoS One 7, e40532, 10.1371/journal.pone.0040532 (2012). 22792363 PMC3391268

[b41] Michael T. P. Plant genome size variation: bloating and purging DNA. Brief Funct Genomics 13, 308–317, 10.1093/bfgp/elu005 (2014). 24651721

[b42] de Koning A. P., Gu W., Castoe T. A., Batzer M. A. & Pollock D. D. Repetitive elements may comprise over two-thirds of the human genome. PLoS Genet 7, e1002384, 10.1371/journal.pgen.1002384 (2011). 22144907 PMC3228813

[b43] Blass E., Bell M. & Boissinot S. Accumulation and rapid decay of non-LTR retrotransposons in the genome of the three-spine stickleback. Genome Biol Evol 4, 687–702, 10.1093/gbe/evs044 (2012). 22534163 PMC3381678

[b44] Hawkins J. S., Proulx S. R., Rapp R. A. & Wendel J. F. Rapid DNA loss as a counterbalance to genome expansion through retrotransposon proliferation in plants. Proc Natl Acad Sci USA 106, 17811–17816, 10.1073/pnas.0904339106 (2009). 19815511 PMC2764891

[b45] Bensasson D., Petrov D. A., Zhang D. X., Hartl D. L. & Hewitt G. M. Genomic gigantism: DNA loss is slow in mountain grasshoppers. Mol Biol Evol 18, 246–253 (2001). 11158383 10.1093/oxfordjournals.molbev.a003798

[b46] Dinger M. E., Amaral P. P., Mercer T. R. & Mattick J. S. Pervasive transcription of the eukaryotic genome: functional indices and conceptual implications. Brief Funct Genomic Proteomic 8, 407–423, 10.1093/bfgp/elp038 (2009). 19770204

[b47] Clark M. B. . The reality of pervasive transcription. PLoS Biol 9, e1000625; discussion e1001102, 10.1371/journal.pbio.1000625 (2011). 21765801 PMC3134446

[b48] Kang L. . The analysis of large-scale gene expression correlated to the phase changes of the migratory locust. Proc Natl Acad Sci USA 101, 17611–17615, 10.1073/pnas.0407753101 (2004). 15591108 PMC535406

[b49] Tarailo-Graovac M. & Chen N. Using RepeatMasker to identify repetitive elements in genomic sequences. Current Protocols in Bioinformatics, 4.10. 11-14.10. 14 (2009). 10.1002/0471250953.bi0410s25 19274634

[b50] Marco A., Hui J. H., Ronshaugen M. & Griffiths-Jones S. Functional shifts in insect microRNA evolution. Genome Biol Evol 2, 686–696, 10.1093/gbe/evq053 (2010). 20817720 PMC2956262

[b51] Langmead B. & Salzberg S. L. Fast gapped-read alignment with Bowtie 2. Nat Methods 9, 357–359, 10.1038/nmeth.1923 (2012). 22388286 PMC3322381

[b52] Langmead B., Trapnell C., Pop M. & Salzberg S. L. Ultrafast and memory-efficient alignment of short DNA sequences to the human genome. Genome Biol 10, R25, 10.1186/gb-2009-10-3-r25 (2009). 19261174 PMC2690996

[b53] Li H. . The Sequence Alignment/Map format and SAMtools. Bioinformatics 25, 2078–2079, 10.1093/bioinformatics/btp352 (2009). 19505943 PMC2723002

[b54] Robinson M. D., McCarthy D. J. & Smyth G. K. edgeR: a Bioconductor package for differential expression analysis of digital gene expression data. Bioinformatics 26, 139–140, 10.1093/bioinformatics/btp616 (2010). 19910308 PMC2796818

