# Supplementary material for: Evidence for the expression of abundant microRNAs in the locust genome
Source: Sci Rep. 2015 Sep 2;5:13608. doi: 10.1038/srep13608 (PMC4556993; doi:10.1038/srep13608)
Supplement: Supplementary Information [file srep13608-s1.pdf]

Evidence for the expression of abundant microRNAs in the locust genome

Yanli Wang[1, 2, *], Feng Jiang[2, *], Huimin Wang[2], Tianqi Song[1], Yuanyuan Wei[3], Meiling Yang[3], Jianzhen Zhang[1], Le Kang[2, 3]

[1] Institute of Applied Biology, Shanxi University, Taiyuan, Shanxi, China

[2] Beijing Institutes of Life Science, Chinese Academy of Sciences, Beijing, China

[3] Institute of Zoology, Chinese Academy of Sciences, Beijing, China

*These authors contributed equally to this study.

Running head: Genome-wide characterization of miRNAs in locusts

Supplemental Data

Supplementary Figure S1

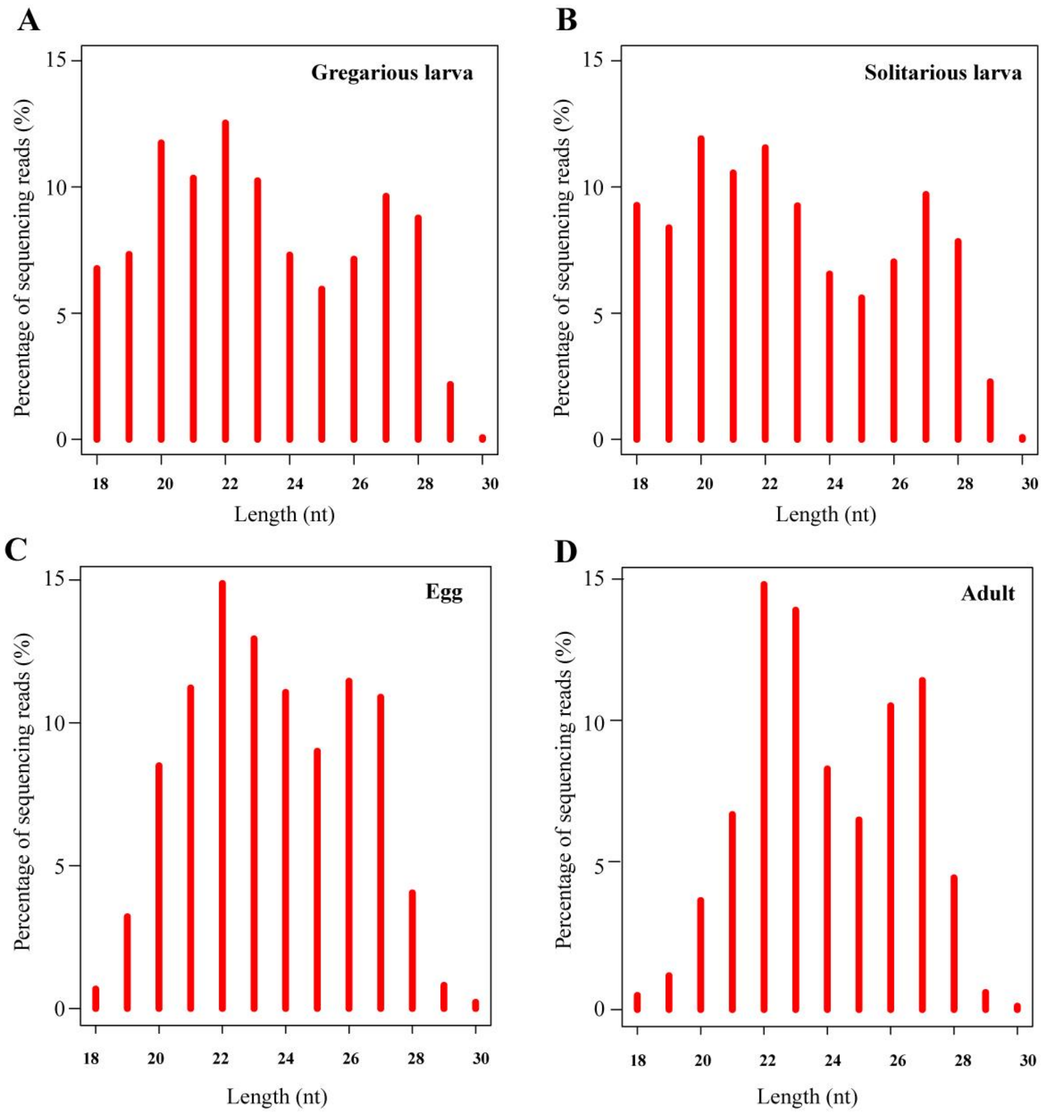

Figure S1. Size distribution of sequencing reads. The size distribution of sequencing reads was identified by high-throughput sequencing of small RNA transcriptome of (A) gregarious and (B) solitarious fourth-instar larvae, (C) eggs, and (D) adults using Illumina Genome Analyzer IIx sequencing system. Nucleotide, nt.

Supplementary Figure S2

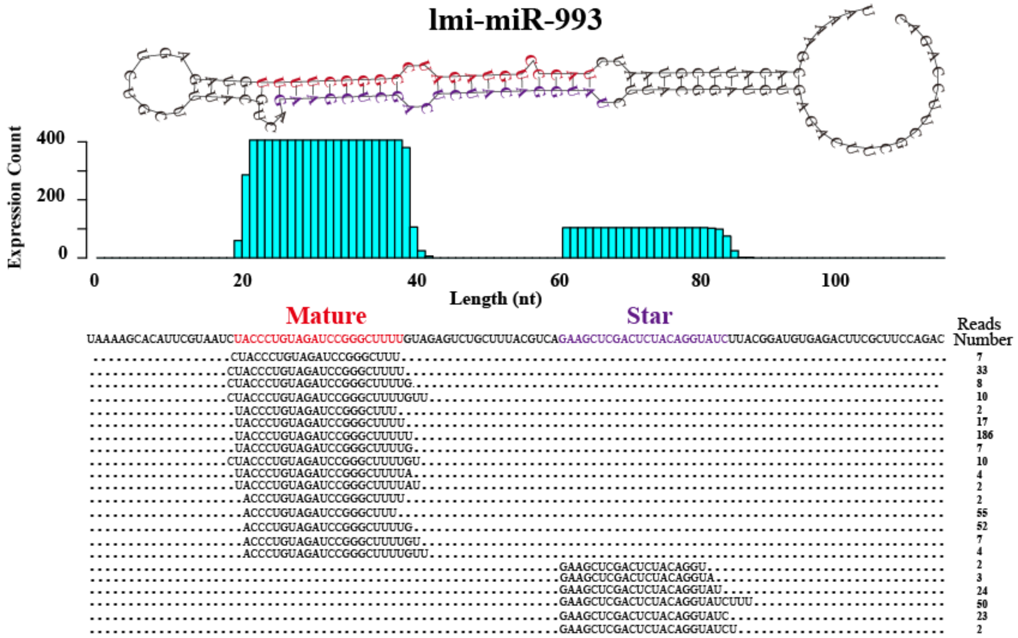

Figure S2. Example of *Locusta migratoria* miRNA identified from high-throughput sequencing. The miRNA precursor (113 nt long) is located at the scaffold16951 of the locust genome assembly along with reads aligned to this precursor. The total number of unique sequencing reads that aligned to the miRNA precursor is listed at the right side of alignment. Their expression levels in the small RNA transcriptomes were used in the prediction of the mature miRNA and star sequences. The predicted mature miRNA and star sequences are shown in red and purple, respectively. A graphic illustration of the hairpin-loop structure of this miRNA precursor is shown on top of the figure. The homology searches against miRBase confirmed this miRNA precursor as an authentic arthropod-specific miRNA gene, which is homologous to the evolutionarily conserved miR-993 gene.

Supplementary Figure S3

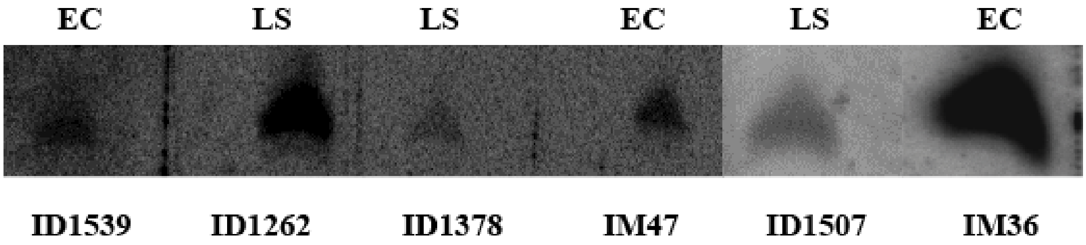

Figure S3. Northern blot validation of miRNA expression. Northern blot analysis was performed for randomly selected miRNAs in pronotums. LS, lineage-specific; EC, evolutionary conserved.

Supplementary Figure S4

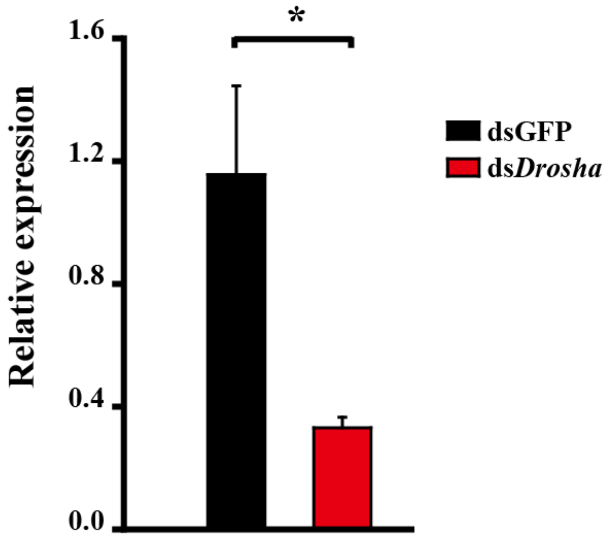

Figure S4. Expression levels of *Drosha* after RNAi knockdown. In the gregarious locusts, the expression level of *Drosha* was significantly reduced 3 d after injection of dsRNA ($n = 8$ each). The asterisks above the strip indicate the significance of treatments after Student's *t*-test (*, P = 0.011).

Supplementary Figure S5

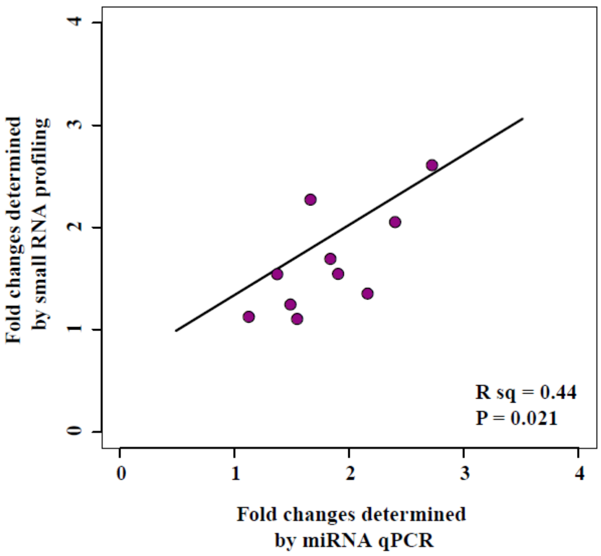

Figure S5. Correlation between fold changes in miRNA expression determined by miRNA qPCR and small RNA profiling after RNAi knockdown. In gregarious locusts, the expression level of miRNAs was significantly reduced 3 d after injection of *Drosha* dsRNA. The fold changes in Green fluorescent protein controls and dsRNA-treated samples were determined by miRNA qPCR ($n = 8$ each) and small RNA profiling, respectively.

Supplementary Figure S6

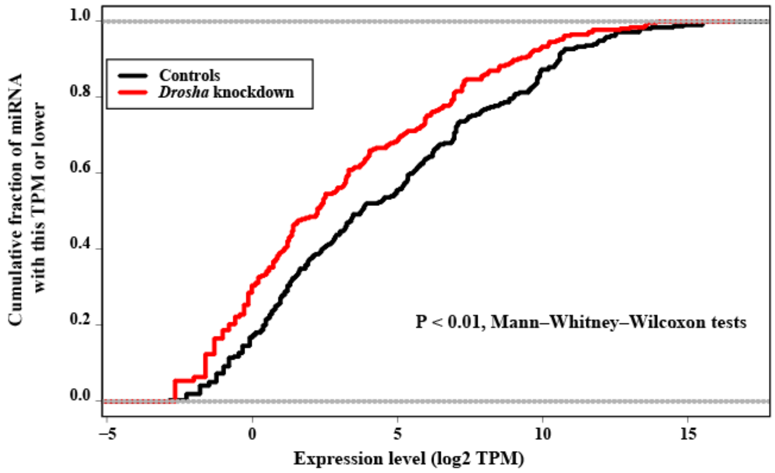

Figure S6. Expression quantification of the predicted miRNAs in the control and *Drosha* knockdown pronotums. TPM, transcripts per million.

Supplementary Methods

Northern blotting

Total RNA was extracted using TRIzol reagent (Invitrogen). 100 ug of total RNA was separated on a 15% 7M Urea PAGE and transferred to Hybond N+ (Amersham Biosciences) membranes by wet transfer for 12 hours at 14V. Membranes were cross-linked by chemical crosslinking agents for 2 hours at 60 ℃ and for 2 hours at 85 ℃. MiRNA complementary probes were end-labelled with gamma-P32-adenosine triphosphate using T4 polynucleotide kinase (New England BioLabs). The membranes were hybridized at 37 ℃ with hybridization buffer for overnight. Final washes of the membranes were performed at 37 ℃ with wash buffer. The washed membrane was then wrapped and exposed on a PhosphorImager screen.

Table S1. Summary of miRNAs in the *Locusta migratoria* genome

Genomic Accessions	Annotation Methods	Begin	End	Score	Rnafold	miRNA Accessions	Mature miRNA	Sequences of mature miRNA	Homologous microRNAs
78907018	miRDeep2	117	228	3099.5	rnafold:yes	ID=ID1181	Mature:ID1181-5P:c178907018:194..216:-	TGATGCTGCAGGAGTTGTTGTGA	-
c181814947	miRDeep2	88	199	4347.4	rnafold:yes	ID=ID1275	Mature:ID1275-3P:c181814947:108..129:-	TGGACTGTGCTAGCAGCGGACT	-
c186012159	miRDeep2	166	277	20855.2	rnafold:yes	ID=ID1149	Mature:ID1149-5P:c186012159:236..257:-	TGGAACACGAGGTAGATTTGTC	-
c186085701	miRDeep2	362	473	26228.8	rnafold:yes	ID=ID914	Mature:ID914-5P:c186085701:382..404:+	TCTTCGTCGCGGTTTGCTGCCAT	-
c188188083	miRDeep2	133	240	137654.6	rnafold:yes	ID=ID1254	Mature:ID1254-3P:c188188083:203..223:+	TGACTAGATCCACACTCATCA	tur-miR-279
c188608704	miRDeep2	471	579	1152.9	rnafold:yes	ID=ID1619	Mature:ID1619-3P:c188608704:541..561:+	TCTGTCAGCACTCAGTGGACA	-
c189365984	miRDeep2	152	264	164.8	rnafold:yes	ID=ID1550	Mature:ID1550-3P:c189365984:172..194:-	CATAACGCTCTTTGGTCAGCTGT	-
c190111347	miRDeep2	325	436	22	rnafold:yes	ID=ID1132	Mature:ID1132-3P:c190111347:345..366:-	TCACCTGACGATGGCGGAACGA	-
scaffold1009	miRDeep2	164580	164689	91.6	rnafold:yes	ID=ID1353	Mature:ID1353-3P:scaffold1009:164613..164634:-	ACCTAGATCTGATTATAACTAG	-
scaffold1009	miRDeep2	164594	164705	9035.2	rnafold:yes	ID=ID1354	Mature:ID1354-5P:scaffold1009:164614..164635:+	TAGTTATAATCAGATCTAGGTT	-
scaffold10116	miRDeep2	69074	69185	47	rnafold:yes	ID=ID136	Mature:ID136-3P:scaffold10116:69094..69116:-	GGCATCTGTTCGGTGAAGCTGAG	-
scaffold10193	miRDeep2	229826	229938	55775.5	rnafold:yes	ID=ID71	Mature:ID71-3P:scaffold10193:229846..229868:-	TAAATGCACTATCTGGTACGACA	tca-miR-277
scaffold10193	miRDeep2	194923	195036	5800	rnafold:yes	ID=ID72	Mature:ID72-5P:scaffold10193:194993..195016:-	TGGCAGTGTGGTTAGCTGGTTGTG	ame-miR-34
scaffold10227	miRDeep2	351532	351640	10025.3	rnafold:yes	ID=ID1119	Mature:ID1119-3P:scaffold10227:351602..351622:+	TTCGTTGTCGACGAAACCTGC	api-miR-981
scaffold103	miRDeep2	339367	339475	14343.4	rnafold:yes	ID=ID1086	Mature:ID1086-5P:scaffold103:339387..339408:+	GCAGAACTCAGACATTTACTAG	-
scaffold1033	miRDeep2	3E+06	3E+06	36	rnafold:yes	ID=ID36	Mature:ID36-5P:scaffold1033:2793037..2793059:-	ACTGACGTACTGCCGGTTCATAG	-
scaffold10339	miRDeep2	563304	563416	1356.6	rnafold:yes	ID=ID1176	Mature:ID1176-3P:scaffold10339:563374..563396:+	AGCCAACCGCTCGTGACTACACA	-
scaffold10435	miRDeep2	87729	87840	581	rnafold:yes	ID=ID456	Mature:ID456-3P:scaffold10435:87749..87770:-	ATATACATAATCGGTGCTGTAT	-
scaffold10435	miRDeep2	45484	45595	108.7	rnafold:yes	ID=ID458	Mature:ID458-3P:scaffold10435:45504..45526:-	GATCTACATAATCGGTGCTGTGT	-
scaffold10456	miRDeep2	319300	319408	1969.6	rnafold:yes	ID=ID659	Mature:ID659-3P:scaffold10456:319370..319390:+	CATCGCCTGGGATCCCTGATG	-
scaffold10456	miRDeep2	815236	815347	782.7	rnafold:yes	ID=ID660	Mature:ID660-5P:scaffold10456:815298..815320:-	AGAAGACAAGGCCTGACAACTTG	-
scaffold10690	miRDeep2	342899	343010	18.9	rnafold:yes	ID=ID177	Mature:ID177-3P:scaffold10690:342919..342940:-	ATATACATAATCGGTGCTGTAT	-

scaffold108	miRDeep2	190145	190256	341547.9	rnafold:yes	ID=ID1624	Mature:ID1624-5P:scaffold108:190215..190236:-	AACCCGTAGATCCGAACTTGTG	dps-miR-100
scaffold108	miRDeep2	190006	190117	932163.2	rnafold:yes	ID=ID1625	Mature:ID1625-5P:scaffold108:190076..190097:-	TGAGGTAGTAGGTTGTATAGTT	tca-let-7
scaffold10840	miRDeep2	40719	40828	24.3	rnafold:yes	ID=ID1228	Mature:ID1228-5P:scaffold10840:40738..40758:+	GTGGGCAGACATTTGTAGAAA	-
scaffold10863	miRDeep2	55426	55533	6069.6	rnafold:yes	ID=ID1234	Mature:ID1234-3P:scaffold10863:55496..55517:+	TACTGGCCTGCTAAGTCCCAAG	dme-miR-193
scaffold10863	miRDeep2	55682	55789	1378.4	rnafold:yes	ID=ID1235	Mature:ID1235-3P:scaffold10863:55752..55773:+	CAATGCCCTTGGAAATCCCAAA	ame-miR-2788
scaffold11042	miRDeep2	30970	31081	2238.4	rnafold:yes	ID=ID1647	Mature:ID1647-5P:scaffold11042:31040..31061:-	TCGAGATTCCTGGAATGCCACG	-
scaffold11042	miRDeep2	36397	36508	1333.1	rnafold:yes	ID=ID1648	Mature:ID1648-5P:scaffold11042:36467..36488:-	TACAGGACTCTGCAACTTACCG	-
scaffold11070	miRDeep2	238320	238430	18.5	rnafold:yes	ID=ID15	Mature:ID15-5P:scaffold11070:238376..238398:-	AGGTGGAGGAATGCCTTTGTACG	-
scaffold11070	miRDeep2	118782	118893	23.4	rnafold:yes	ID=ID18	Mature:ID18-5P:scaffold11070:118852..118873:-	CGATGGAATTGTGAACCCTGGC	-
scaffold11154	miRDeep2	167917	168027	898.5	rnafold:yes	ID=ID184	Mature:ID184-3P:scaffold11154:167937..167958:-	TCGAGTGCAGTAACTTCCCCGA	-
scaffold11158	miRDeep2	6537	6647	166.4	rnafold:yes	ID=ID553	Mature:ID553-3P:scaffold11158:6607..6628:+	TGTTAAAGTGCAGTTCAGCCGG	-
scaffold11197	miRDeep2	230605	230717	6190.1	rnafold:yes	ID=ID196	Mature:ID196-5P:scaffold11197:230675..230698:-	CGACACATTCGTGGGACTCTGTGC	-
scaffold11197	miRDeep2	184214	184325	1947	rnafold:yes	ID=ID197	Mature:ID197-5P:scaffold11197:184284..184306:-	TGACGTTTGCATCAAACAGCTGC	-
scaffold11241	miRDeep2	36695	36808	55.1	rnafold:yes	ID=ID1495	Mature:ID1495-3P:scaffold11241:36715..36738:-	TATTACAAAATTTGAGAGTCCAT	-
scaffold1153	miRDeep2	2E+06	2E+06	46.2	rnafold:yes	ID=ID675	Mature:ID675-3P:scaffold1153:2368548..2368569:+	TTCGGATCTCTTACTCAGGAAG	-
scaffold116858	miRDeep2	107	218	46.6	rnafold:yes	ID=ID294	Mature:ID294-3P:scaffold116858:177..199:+	ATGCTGCTGTCTCTCCTGCTGCT	-
scaffold11774	miRDeep2	52186	52298	308.3	rnafold:yes	ID=ID679	Mature:ID679-5P:scaffold11774:52256..52278:-	TTTGTTGATATTTTCGATGCACT	-
scaffold11860	miRDeep2	287476	287587	607.5	rnafold:yes	ID=ID1748	Mature:ID1748-3P:scaffold11860:287496..287517:-	TACCAGCCGGATATTCGACTGT	-
scaffold11864	miRDeep2	17739	17852	149895.3	rnafold:yes	ID=ID1321	Mature:ID1321-5P:scaffold11864:17809..17832:-	TGGAAGACTAGTGATTTTGTTGT	tca-miR-7
scaffold12210	miRDeep2	747970	748079	1924.8	rnafold:yes	ID=ID19	Mature:ID19-3P:scaffold12210:748040..748061:+	TACAGAAGACGTGTGTCAGAGA	-
scaffold122787	miRDeep2	331	440	56918.6	rnafold:yes	ID=ID1226	Mature:ID1226-3P:scaffold122787:401..423:+	TACGGCCTGAAGATGTTCCTAGT	-
scaffold124129	miRDeep2	5043	5152	453.2	rnafold:yes	ID=ID1494	Mature:ID1494-3P:scaffold124129:5068..5085:-	GTGCTGTCAGCGATTACT	-
scaffold1251	miRDeep2	318397	318509	12	rnafold:yes	ID=ID241	Mature:ID241-3P:scaffold1251:318417..318439:-	AGCAACTCGGCAGCCAACCTGGC	-
scaffold1251	miRDeep2	319205	319317	399	rnafold:yes	ID=ID242	Mature:ID242-3P:scaffold1251:319226..319248:-	AGCAACTTGGCAGTCGGCCCATC	-
scaffold1251	miRDeep2	593706	593817	1164.6	rnafold:yes	ID=ID243	Mature:ID243-3P:scaffold1251:593726..593748:-	CAGACGTCTCGATGTACTCCCCA	-

scaffold1251	miRDeep2	411698	411809	13.9	rnafold:yes	ID=ID245	Mature:ID245-3P:scaffold1251:411718..411739:-	CATCTGGCAGGGGCGCCACCCA	-
scaffold1251	miRDeep2	605384	605495	137.4	rnafold:yes	ID=ID246	Mature:ID246-3P:scaffold1251:605404..605426:-	CAGGTGCCTCGCTATGCTCCACC	-
scaffold1251	miRDeep2	280569	280680	64.9	rnafold:yes	ID=ID247	Mature:ID247-5P:scaffold1251:280639..280660:-	GGGGAGCTGGCTGCCGATTGCT	-
scaffold1251	miRDeep2	315325	315437	164.4	rnafold:yes	ID=ID248	Mature:ID248-5P:scaffold1251:315382..315403:-	TGGGGTGGCTGCCGGGTGCTGT	-
scaffold1251	miRDeep2	329077	329189	888.7	rnafold:yes	ID=ID250	Mature:ID250-3P:scaffold1251:329097..329120:-	AGCAACTCAGCAGTCGGCCCATCC	-
scaffold1251	miRDeep2	328272	328383	254.7	rnafold:yes	ID=ID252	Mature:ID252-5P:scaffold1251:328342..328363:-	TGGGTCGGCTGCCGGGTGCTGT	-
scaffold1251	miRDeep2	392192	392301	258.5	rnafold:yes	ID=ID254	Mature:ID254-3P:scaffold1251:392212..392233:-	ACTATTTGACAGCCAGTCCCCC	-
scaffold1251	miRDeep2	338522	338633	75	rnafold:yes	ID=ID256	Mature:ID256-5P:scaffold1251:338592..338613:-	GGAGAGTCGGCTGCCAGTTGCT	-
scaffold1251	miRDeep2	317551	317662	360.7	rnafold:yes	ID=ID257	Mature:ID257-5P:scaffold1251:317621..317642:-	TGGGTCGGCTGCCGGGTGCTGT	-
scaffold1251	miRDeep2	401444	401553	258.4	rnafold:yes	ID=ID258	Mature:ID258-3P:scaffold1251:401464..401485:-	ACTATTTGACAGCCAGTCCCCC	-
scaffold12662	miRDeep2	115386	115495	6718.9	rnafold:yes	ID=ID877	Mature:ID877-3P:scaffold12662:115456..115477:+	ATCCCCTGGTGAAACTTTCCGC	-
scaffold12835	miRDeep2	350145	350256	181.8	rnafold:yes	ID=ID670	Mature:ID670-3P:scaffold12835:350165..350186:-	AATTTTATGAGCAACCGTGGGG	-
scaffold12835	miRDeep2	349753	349865	42.8	rnafold:yes	ID=ID671	Mature:ID671-3P:scaffold12835:349773..349795:-	GATGTTATGAGGGACCTTAGAGT	-
scaffold13134	miRDeep2	18208	18319	429.9	rnafold:yes	ID=ID562	Mature:ID562-3P:scaffold13134:18278..18299:+	TCTCTGAGACCCATACGTCACT	-
scaffold13134	miRDeep2	12207	12318	187.1	rnafold:yes	ID=ID564	Mature:ID564-3P:scaffold13134:12277..12298:+	TTGCTGAGGCCCATACGTTACT	-
scaffold13134	miRDeep2	6518	6629	194.6	rnafold:yes	ID=ID565	Mature:ID565-3P:scaffold13134:6588..6609:+	TATCTGAGGCCCATTCGTTACT	-
scaffold133197	miRDeep2	5247	5358	188.3	rnafold:yes	ID=ID816	Mature:ID816-5P:scaffold133197:5307..5329:-	CCTCTGAGTGCTGGACAGAGGGC	-
scaffold133197	miRDeep2	8785	8896	902.2	rnafold:yes	ID=ID817	Mature:ID817-5P:scaffold133197:8855..8876:-	TGGCTGAGTGCTGGCCAGAGGG	-
scaffold13443	miRDeep2	239766	239878	444639.2	rnafold:yes	ID=ID929	Mature:ID929-3P:scaffold13443:239786..239808:-	GTAGGCCGGCGGAAACTACTTGC	tca-miR-2796
scaffold137	miRDeep2	156828	156939	103.7	rnafold:yes	ID=ID153	Mature:ID153-5P:scaffold137:156848..156869:+	TCACTGTTGCCATAGTTCCGGG	-
scaffold137	miRDeep2	95349	95458	264.7	rnafold:yes	ID=ID155	Mature:ID155-3P:scaffold137:95419..95440:+	GAGGGGGGCTGTGTGTGGTGCC	-
scaffold137	miRDeep2	172135	172242	1648.4	rnafold:yes	ID=ID156	Mature:ID156-3P:scaffold137:172205..172226:+	GAAGGCTTCAGGTGGAGAACGC	-
scaffold137	miRDeep2	140966	141073	4347.4	rnafold:yes	ID=ID157	Mature:ID157-3P:scaffold137:141036..141057:+	TGGACTGTGCTAGCAGCGGACT	-
scaffold137	miRDeep2	164161	164268	1224.9	rnafold:yes	ID=ID158	Mature:ID158-3P:scaffold137:164231..164252:+	ATATAGCTGTCCTGTCCGCGTC	-
scaffold13802	miRDeep2	227982	228093	488.8	rnafold:yes	ID=ID1242	Mature:ID1242-3P:scaffold13802:228002..228024:-	TGGATGGTAAGCCTATCGACGTG	-

scaffold13892	miRDeep2	758868	758977	506.5	rnafold:yes	ID=ID737	Mature:ID737-3P:scaffold13892:758938..758957:+	TCAGCCTGATTGCTAGGCGA	-
scaffold14243	miRDeep2	115711	115821	64477.8	rnafold:yes	ID=ID1345	Mature:ID1345-5P:scaffold14243:115731..115753:+	TAATCTCATGTGGTAACTGTGAG	ame-miR-3477
scaffold14243	miRDeep2	117386	117497	21575.9	rnafold:yes	ID=ID1346	Mature:ID1346-5P:scaffold14243:117406..117427:+	TGAGTATTACATCAGGTACTGA	tur-miR-12a
scaffold14243	miRDeep2	110422	110529	18935.5	rnafold:yes	ID=ID1347	Mature:ID1347-5P:scaffold14243:110442..110463:+	AAATATCAGTTGGTAATTCTGG	tca-miR-283
scaffold1425	miRDeep2	593916	594028	1065.8	rnafold:yes	ID=ID1465	Mature:ID1465-3P:scaffold1425:593986..594010:+	GGCATGTCCTTGGGAAAAGTAACG	-
scaffold1426	miRDeep2	176468	176579	13.5	rnafold:yes	ID=ID1790	Mature:ID1790-5P:scaffold1426:176538..176559:-	AGGAGGCGACAATAGCTGCCAA	-
scaffold1426	miRDeep2	248401	248513	40399	rnafold:yes	ID=ID1791	Mature:ID1791-3P:scaffold1426:248421..248443:-	TGTCTACCTCCACTGATCCCGCT	-
scaffold14267	miRDeep2	1E+06	1E+06	4283.1	rnafold:yes	ID=ID862	Mature:ID862-5P:scaffold14267:1019379..1019402:	TCCAAATGATTTCCCACAGGCTGC	-
scaffold14267	miRDeep2	992411	992520	183790.8	rnafold:yes	ID=ID863	Mature:ID863-5P:scaffold14267:992431..992453:+	TCTGAAGGATTTGTTACAGACTG	-
scaffold14267	miRDeep2	995012	995120	408354.1	rnafold:yes	ID=ID864	Mature:ID864-5P:scaffold14267:995032..995054:+	TCTAAAGGATTTGTTACAGACTG	-
scaffold14267	miRDeep2	966637	966746	5741.4	rnafold:yes	ID=ID865	Mature:ID865-5P:scaffold14267:966657..966678:+	TCTAAAGTATTTGTTACAGACT	-
scaffold14267	miRDeep2	993610	993719	183790.8	rnafold:yes	ID=ID866	Mature:ID866-5P:scaffold14267:993630..993652:+	TCTGAAGGATTTGTTACAGACTG	-
scaffold14267	miRDeep2	1E+06	1E+06	2805.8	rnafold:yes	ID=ID867	Mature:ID867-5P:scaffold14267:1018524..1018546:	TGTAAAGGACTTGCCACAAGCTG	-
scaffold14267	miRDeep2	1E+06	1E+06	183905.6	rnafold:yes	ID=ID868	Mature:ID868-5P:scaffold14267:1006087..1006109:	TCTGAAGGATTTGTTACAGACTG	-
scaffold14267	miRDeep2	963304	963412	408157.2	rnafold:yes	ID=ID869	Mature:ID869-5P:scaffold14267:963324..963346:+	TCTAAAGGATTTGTTACAGACTG	-
scaffold14267	miRDeep2	1E+06	1E+06	12	rnafold:yes	ID=ID870	Mature:ID870-5P:scaffold14267:1006852..1006875:	TGTGAAAGATTTGCCACAAACTGT	-
scaffold1444	miRDeep2	1E+06	1E+06	8909.3	rnafold:yes	ID=ID1271	Mature:ID1271-3P:scaffold1444:1141855..1141876:-	CCAGCAAAGTGGAAGAGGCCGA	ame-miR-316
scaffold14451	miRDeep2	64765	64876	1558.4	rnafold:yes	ID=ID782	Mature:ID782-5P:scaffold14451:64835..64856:-	TCGCGCAGCTGGAGACCTTCGG	-
scaffold14451	miRDeep2	70676	70789	22.4	rnafold:yes	ID=ID783	Mature:ID783-5P:scaffold14451:70746..70769:-	ACCCCTGTCGGCTGCCTCTTCGCT	-
scaffold14451	miRDeep2	101979	102090	285.5	rnafold:yes	ID=ID784	Mature:ID784-3P:scaffold14451:101999..102021:-	CGAAAGGGGCCGACAGTGGACGC	-
scaffold14451	miRDeep2	129374	129485	1648.3	rnafold:yes	ID=ID785	Mature:ID785-3P:scaffold14451:129394..129415:-	GAAGGCTTCAGGTGGAGAACGC	-
scaffold14451	miRDeep2	104661	104773	106.6	rnafold:yes	ID=ID787	Mature:ID787-5P:scaffold14451:104731..104753:-	TCTCACAGCTGGAAATCTTCGGA	-
scaffold14451	miRDeep2	121241	121353	5624	rnafold:yes	ID=ID788	Mature:ID788-5P:scaffold14451:121311..121334:-	TCTCACAGCTGGAGATCTTTGGAG	-
scaffold14451	miRDeep2	99820	99931	2312.3	rnafold:yes	ID=ID789	Mature:ID789-5P:scaffold14451:99890..99911:-	TCGCGCAGCTGGAGACATTCGG	-
scaffold14451	miRDeep2	111191	111302	2484.2	rnafold:yes	ID=ID791	Mature:ID791-5P:scaffold14451:111261..111282:-	TCGCGCAGCTGGAGACATTCGG	-

scaffold14451	miRDeep2	54457	54568	285.2	rnafold:yes	ID=ID792	Mature:ID792-3P:scaffold14451:54477..54499:-	CGAAAGGGGCCGACAGTGGACGC	-
scaffold14451	miRDeep2	70106	70217	29	rnafold:yes	ID=ID793	Mature:ID793-5P:scaffold14451:70165..70187:-	TCCCGCAACCGGCGACCCTCGGA	-
scaffold1446	miRDeep2	558093	558200	2748.7	rnafold:yes	ID=ID878	Mature:ID878-5P:scaffold1446:558113..558133:+	GTGCATTGTAGTTGCATTGCA	dme-miR-33
scaffold1454	miRDeep2	6E+06	6E+06	3868.6	rnafold:yes	ID=ID822	Mature:ID822-5P:scaffold1454:6164889..6164911:-	CCCATCATCAGGTGCAGTCGCCT	-
scaffold1454	miRDeep2	6E+06	6E+06	3918.3	rnafold:yes	ID=ID823	Mature:ID823-5P:scaffold1454:6156741..6156763:-	CCCATCATCAGGTGCAGTCGCCT	-
scaffold1454	miRDeep2	6E+06	6E+06	2632.5	rnafold:yes	ID=ID824	Mature:ID824-5P:scaffold1454:6185313..6185335:-	CCCATCATCAGGTGCAGTCGTTC	-
scaffold1454	miRDeep2	6E+06	6E+06	1596.5	rnafold:yes	ID=ID826	Mature:ID826-5P:scaffold1454:6168009..6168031:-	TCCATCACCAGGTGCAGCCGTCT	-
scaffold1454	miRDeep2	6E+06	6E+06	1992.9	rnafold:yes	ID=ID827	Mature:ID827-3P:scaffold1454:6162052..6162074:-	CGACTGCACCTGACGGCGGAGGC	-
scaffold149847	miRDeep2	582	691	530.1	rnafold:yes	ID=ID1365	Mature:ID1365-3P:scaffold149847:652..674:+	ATGCTTCTGTTTCTCCTGCTGCT	-
scaffold15022	miRDeep2	237668	237781	18	rnafold:yes	ID=ID1183	Mature:ID1183-5P:scaffold15022:237738..237762:-	GCTTTCTTCTTGTGGTTTGACTGT	-
scaffold15123	miRDeep2	122602	122713	70	rnafold:yes	ID=ID1458	Mature:ID1458-5P:scaffold15123:122622..122644:+	AGATGGAGTTGAGCCGGAATACG	-
scaffold15123	miRDeep2	74127	74238	70	rnafold:yes	ID=ID1460	Mature:ID1460-5P:scaffold15123:74147..74169:+	AGATGGAGTTGAGCCGGAATACG	-
scaffold152	miRDeep2	2E+06	2E+06	30.5	rnafold:yes	ID=ID186	Mature:ID186-3P:scaffold152:2215927..2215948:+	TCCGGCAGCAAACCCGAAGAGA	-
scaffold153536	miRDeep2	6713	6824	5846.2	rnafold:yes	ID=ID639	Mature:ID639-3P:scaffold153536:6783..6804:+	CCTGCTCCAGCACTCAGAGGGG	-
scaffold15492	miRDeep2	28756	28867	1004	rnafold:yes	ID=ID366	Mature:ID366-5P:scaffold15492:28776..28797:+	ACGTGCATCCCCAATGGAGTCG	-
scaffold1572	miRDeep2	871215	871328	147565	rnafold:yes	ID=ID803	Mature:ID803-5P:scaffold1572:871285..871308:-	ATTGTACTTCATCAGGTGCTCTGG	dpu-miR-305
scaffold1572	miRDeep2	61131	61245	11.7	rnafold:yes	ID=ID804	Mature:ID804-3P:scaffold1572:61151..61175:-	TCAGTGTAAGGACTTGTACCATGG	-
scaffold1572	miRDeep2	871360	871471	630322.6	rnafold:yes	ID=ID805	Mature:ID805-3P:scaffold1572:871380..871401:-	TCAGGTACCTGAAGTAGCGCGC	aae-miR-275
scaffold16487	miRDeep2	74770	74880	23715.6	rnafold:yes	ID=ID1443	Mature:ID1443-5P:scaffold16487:74790..74810:+	CGTTAATTTCATGTGCAAGCA	-
scaffold16642	miRDeep2	93322	93436	10.7	rnafold:yes	ID=ID1584	Mature:ID1584-5P:scaffold16642:93372..93394:-	GCGGATGAGATTGCAGAAGCGTG	-
scaffold1665	miRDeep2	802503	802614	10.6	rnafold:yes	ID=ID1764	Mature:ID1764-5P:scaffold1665:802573..802594:-	TACGAAAAATTCATCATCCGCC	-
scaffold16691	miRDeep2	200229	200339	16	rnafold:yes	ID=ID661	Mature:ID661-5P:scaffold16691:200249..200271:+	TGATAGATCTTCCAGGTTGTAAG	-
scaffold16948	miRDeep2	107438	107549	7102.5	rnafold:yes	ID=ID1735	Mature:ID1735-3P:scaffold16948:107458..107479:-	CTGGGTACTCCAATGTCAGGGG	-
scaffold16951	miRDeep2	29893	30005	1445.4	rnafold:yes	ID=ID445	Mature:ID445-3P:scaffold16951:29913..29935:-	GAAGCTCGACTCTACAGGTATCT	dme-miR-993
scaffold1753	miRDeep2	140324	140433	1203.9	rnafold:yes	ID=ID189	Mature:ID189-3P:scaffold1753:140394..140414:+	TTATTGCTTGAGAATACACGT	tca-miR-137

scaffold1753	miRDeep2	137767	137876	1203.9	rnafold:yes	ID=ID190	Mature:ID190-3P:scaffold1753:137837..137857:+	TTATTGCTTGAGAATACACGT	tca-miR-137
scaffold1755	miRDeep2	2E+06	2E+06	24.2	rnafold:yes	ID=ID102	Mature:ID102-5P:scaffold1755:2304054..2304075:-	TTTCGAATGTATTCCTATCACG	-
scaffold1755	miRDeep2	2E+06	2E+06	167.4	rnafold:yes	ID=ID103	Mature:ID103-3P:scaffold1755:2196657..2196678:-	TCTCTGAGACCCATACGTCACT	-
scaffold1755	miRDeep2	2E+06	2E+06	401.1	rnafold:yes	ID=ID81	Mature:ID81-3P:scaffold1755:2168745..2168766:-	TGCCACAACCACATACGTCACT	-
scaffold1755	miRDeep2	2E+06	2E+06	252.2	rnafold:yes	ID=ID82	Mature:ID82-5P:scaffold1755:2332460..2332481:-	TCACAGAAGCGTTAGTTGCACA	-
scaffold1755	miRDeep2	2E+06	2E+06	3098	rnafold:yes	ID=ID84	Mature:ID84-5P:scaffold1755:2337861..2337883:-	CGGTGGAAACCTACACGAACACG	-
scaffold1755	miRDeep2	2E+06	2E+06	93.3	rnafold:yes	ID=ID85	Mature:ID85-3P:scaffold1755:2185560..2185581:-	TACTTGAGGCACATTCGTTACT	-
scaffold1755	miRDeep2	2E+06	2E+06	257.3	rnafold:yes	ID=ID86	Mature:ID86-3P:scaffold1755:2195445..2195466:-	TTCACGGGACCCATACGTTACT	-
scaffold1755	miRDeep2	2E+06	2E+06	262.2	rnafold:yes	ID=ID88	Mature:ID88-3P:scaffold1755:2176531..2176552:-	TTCCTGTGGTACATACGTTACT	-
scaffold1755	miRDeep2	2E+06	2E+06	157.9	rnafold:yes	ID=ID89	Mature:ID89-3P:scaffold1755:2251664..2251685:-	TTGATCAGGCACTTCTAACGAT	-
scaffold1755	miRDeep2	2E+06	2E+06	4629.2	rnafold:yes	ID=ID91	Mature:ID91-5P:scaffold1755:2338798..2338820:-	CGGAGGAAACCTACACGAACACG	-
scaffold1755	miRDeep2	2E+06	2E+06	5615.2	rnafold:yes	ID=ID92	Mature:ID92-3P:scaffold1755:2242884..2242905:-	TCCATTATACACTTCTGACGAC	-
scaffold1755	miRDeep2	2E+06	2E+06	77	rnafold:yes	ID=ID94	Mature:ID94-5P:scaffold1755:2273001..2273022:-	ATTCGGATGTATTCCCATCACG	-
scaffold1755	miRDeep2	2E+06	2E+06	378.2	rnafold:yes	ID=ID96	Mature:ID96-3P:scaffold1755:2310075..2310095:-	TGGCAGGTACATCTTTGATGG	-
scaffold1755	miRDeep2	2E+06	2E+06	3707.9	rnafold:yes	ID=ID97	Mature:ID97-5P:scaffold1755:2338276..2338298:-	CGGTGGAAACCTACACGAACACG	-
scaffold1755	miRDeep2	2E+06	2E+06	378.3	rnafold:yes	ID=ID98	Mature:ID98-5P:scaffold1755:2190541..2190563:-	TGACGTATGTGGTGGGGGAACAC	-
scaffold1755	miRDeep2	2E+06	2E+06	1839.7	rnafold:yes	ID=ID99	Mature:ID99-5P:scaffold1755:2332748..2332769:-	TGATGGGCGTGCGATTACACAT	-
scaffold17867	miRDeep2	189718	189825	94139.4	rnafold:yes	ID=ID1454	Mature:ID1454-3P:scaffold17867:189788..189809:+	AAGATCTAGTAGAGACGGCTGC	-
scaffold17867	miRDeep2	178377	178486	1164	rnafold:yes	ID=ID1455	Mature:ID1455-5P:scaffold17867:178397..178417:+	TTGATTATGGGCCGAAGCATT	-
scaffold1821	miRDeep2	409418	409529	12.7	rnafold:yes	ID=ID1540	Mature:ID1540-5P:scaffold1821:409488..409509:-	TGATGTCTGTCTGTATTGTTGA	-
scaffold18345	miRDeep2	73925	74036	48.7	rnafold:yes	ID=ID1626	Mature:ID1626-5P:scaffold18345:73945..73966:+	ATCCTGCATCATGTGCATTGCT	-
scaffold18392	miRDeep2	343590	343700	30.8	rnafold:yes	ID=ID373	Mature:ID373-3P:scaffold18392:343660..343680:+	TTTTATCACCCTCACATCACC	-
scaffold18641	miRDeep2	102483	102594	1031.5	rnafold:yes	ID=ID1310	Mature:ID1310-5P:scaffold18641:102553..102574:-	TAAGCTCGTCTTTCTGAGCAGT	-
scaffold18761	miRDeep2	219833	219942	16442.6	rnafold:yes	ID=ID1131	Mature:ID1131-3P:scaffold18761:219903..219924:+	TCGGTGGGACTTTCGTCCGTTT	bmo-miR-278
scaffold18836	miRDeep2	120172	120282	1764.9	rnafold:yes	ID=ID543	Mature:ID543-5P:scaffold18836:120242..120262:-	TAGAGGTCAGTTGTTAAGATT	-

scaffold18836	miRDeep2	120004	120115	3480.7	rnafold:yes	ID=ID544	Mature:ID544-5P:scaffold18836:120054..120075:-	CAGTGGTCGAGTGGTCTGAGGC	-
scaffold188787	miRDeep2	65350	65463	236.4	rnafold:yes	ID=ID719	Mature:ID719-3P:scaffold188787:65372..65395:-	GTTTACGATAGTAACTCGGGCGTC	-
scaffold1946	miRDeep2	327754	327865	148.2	rnafold:yes	ID=ID1544	Mature:ID1544-3P:scaffold1946:327774..327796:-	TTTCTCGCGGATAATTACTGTTT	-
scaffold19764	miRDeep2	200763	200876	116186	rnafold:yes	ID=ID1292	Mature:ID1292-5P:scaffold19764:200833..200856:-	CTTGGCACTGGAAGAATTCACAGA	dme-miR-263b
scaffold19764	miRDeep2	275220	275332	336870.5	rnafold:yes	ID=ID1293	Mature:ID1293-5P:scaffold19764:275290..275312:-	AATGGCACTGGAAGAATTCACGG	tca-miR-263b
scaffold19949	miRDeep2	202952	203060	921.9	rnafold:yes	ID=ID1595	Mature:ID1595-3P:scaffold19949:203022..203043:+	CTGTCAGACACACAATTTTATT	-
scaffold20043	miRDeep2	411441	411552	167.8	rnafold:yes	ID=ID1793	Mature:ID1793-3P:scaffold20043:411461..411482:-	GCGGTGAGGTATGTAGCAAAGA	-
scaffold20043	miRDeep2	381903	382017	4023.7	rnafold:yes	ID=ID1794	Mature:ID1794-5P:scaffold20043:381973..381997:-	GTGTCCTGTCAGCGTCGATCTTCG	-
scaffold20043	miRDeep2	490533	490644	506.8	rnafold:yes	ID=ID1795	Mature:ID1795-5P:scaffold20043:490603..490624:-	TGTTGCTCTCATCGTCATCATC	-
scaffold20043	miRDeep2	463413	463523	151.2	rnafold:yes	ID=ID1797	Mature:ID1797-3P:scaffold20043:463433..463453:-	TTTCGTGCACCGATCGAACAC	-
scaffold20043	miRDeep2	403717	403828	47.8	rnafold:yes	ID=ID1798	Mature:ID1798-5P:scaffold20043:403787..403808:-	TGTAATACCAGCGCCTGTCTTC	-
scaffold20043	miRDeep2	472684	472793	9992.6	rnafold:yes	ID=ID1799	Mature:ID1799-3P:scaffold20043:472704..472723:-	TAGGAGAAACGAGAGCTACT	-
scaffold20043	miRDeep2	511271	511382	574.4	rnafold:yes	ID=ID1800	Mature:ID1800-3P:scaffold20043:511291..511312:-	TGGTAACTGGCAGAACTTTTTC	-
scaffold20043	miRDeep2	443081	443192	21.5	rnafold:yes	ID=ID1801	Mature:ID1801-3P:scaffold20043:443101..443122:-	TGGTGGATGCCTCAAAGTGCAA	-
scaffold20043	miRDeep2	388000	388110	1290.7	rnafold:yes	ID=ID1802	Mature:ID1802-5P:scaffold20043:388070..388091:-	TGTCCTATCAGTGTCGGTCTTC	-
scaffold20043	miRDeep2	392148	392261	649.6	rnafold:yes	ID=ID1803	Mature:ID1803-5P:scaffold20043:392218..392242:-	TGTGTATTGTCAGCGTCGGTCTTC	-
scaffold20043	miRDeep2	388278	388389	206.4	rnafold:yes	ID=ID1804	Mature:ID1804-5P:scaffold20043:388348..388370:-	TGCCTTATCAGCCTCGGTCTTCG	-
scaffold20043	miRDeep2	409204	409315	2593.3	rnafold:yes	ID=ID1805	Mature:ID1805-5P:scaffold20043:409274..409296:-	TGTCCTATCAGCGTCGGTCTTCG	-
scaffold20043	miRDeep2	472912	473023	314.5	rnafold:yes	ID=ID1806	Mature:ID1806-5P:scaffold20043:472982..473003:-	TGTTGCTCCGTGATTCTATAGT	-
scaffold202156	miRDeep2	2309	2420	312.8	rnafold:yes	ID=ID559	Mature:ID559-3P:scaffold202156:2367..2388:+	GTCTGTGCCTCCTGCTATGAGA	-
scaffold2035	miRDeep2	160722	160830	60120.7	rnafold:yes	ID=ID836	Mature:ID836-3P:scaffold2035:160792..160813:+	TGACTAGATTCACACTCATCCT	tca-miR-279b
scaffold20811	miRDeep2	764431	764539	9470.4	rnafold:yes	ID=ID642	Mature:ID642-5P:scaffold20811:764451..764474:+	AGATATGTTTGATATTCTTGGTT	dme-miR-190
scaffold20942	miRDeep2	680	789	1271.7	rnafold:yes	ID=ID1225	Mature:ID1225-5P:scaffold20942:700..721:+	CGGACAGACTTGAGCTGCGCCG	-
scaffold2111	miRDeep2	233199	233312	580.7	rnafold:yes	ID=ID193	Mature:ID193-5P:scaffold2111:233269..233292:-	TGCGGTTCGCGCCTGGCGGTACGA	-
scaffold213	miRDeep2	1E+06	1E+06	191.3	rnafold:yes	ID=ID673	Mature:ID673-3P:scaffold213:1331840..1331860:-	CTCCCTAACGGAGTCAGGTTG	tca-miR-929

scaffold21393	miRDeep2	166313	166423	30895.7	rnafold:yes	ID=ID387	Mature:ID387-5P:scaffold21393:166333..166354:+	TCAGGAAATCAATCGTGTAAGT	-
scaffold21482	miRDeep2	148040	148149	162.6	rnafold:yes	ID=ID1628	Mature:ID1628-5P:scaffold21482:148110..148131:-	TTGGGCGGACATACTGTAGAAC	-
scaffold2175	miRDeep2	743809	743920	2949.6	rnafold:yes	ID=ID286	Mature:ID286-3P:scaffold2175:743863..743884:+	TATCACATTCTTAGTTCTTTCT	-
scaffold2175	miRDeep2	743471	743582	687.2	rnafold:yes	ID=ID287	Mature:ID287-3P:scaffold2175:743541..743562:+	TCACCGGGTGCAGTTCAATACT	-
scaffold2176	miRDeep2	483728	483838	86.7	rnafold:yes	ID=ID314	Mature:ID314-5P:scaffold2176:483748..483771:+	AAGGATGTAACAGTGGCAGATGGG	-
scaffold22106	miRDeep2	167010	167121	5294.9	rnafold:yes	ID=ID1134	Mature:ID1134-3P:scaffold22106:167080..167102:+	GTACCCGGTCACCTCCTTCCTGA	-
scaffold2219	miRDeep2	5E+06	5E+06	2016.2	rnafold:yes	ID=ID174	Mature:ID174-3P:scaffold2219:5237242..5237264:+	CCGACCAGCCAGTTCGTTCCAGA	-
scaffold22250	miRDeep2	43925	44036	16.1	rnafold:yes	ID=ID319	Mature:ID319-3P:scaffold22250:43995..44017:+	GTTGTATGATGAAATCAAGGCAC	-
scaffold22558	miRDeep2	5616	5724	1304.2	rnafold:yes	ID=ID599	Mature:ID599-3P:scaffold22558:5685..5706:+	TCGGGTCTGCCTCGGTGGCTAG	-
scaffold225892	miRDeep2	898	1010	4222.7	rnafold:yes	ID=ID231	Mature:ID231-5P:scaffold225892:968..991:-	TGTTAGCAAACCCAGCCGCGCTGC	-
scaffold2260	miRDeep2	326962	327071	103.4	rnafold:yes	ID=ID1153	Mature:ID1153-3P:scaffold2260:327032..327052:+	TTACGTGCTCAGCTTTCCTTG	-
scaffold2260	miRDeep2	462992	463104	30.7	rnafold:yes	ID=ID1154	Mature:ID1154-3P:scaffold2260:463062..463084:+	TTCGTGAGTTTTTGCCTCTCTAG	-
scaffold2260	miRDeep2	385930	386040	47.9	rnafold:yes	ID=ID1156	Mature:ID1156-3P:scaffold2260:386000..386021:+	TAATGTCCTTAGCTTCTCTTGC	-
scaffold2260	miRDeep2	335759	335868	30	rnafold:yes	ID=ID1160	Mature:ID1160-5P:scaffold2260:335788..335810:+	AGGGAGAGCTGGGTACATAGAAG	-
scaffold2260	miRDeep2	413795	413902	26.4	rnafold:yes	ID=ID1163	Mature:ID1163-5P:scaffold2260:413822..413844:+	TAGGGGAAGCTAGGGTACTTAGA	-
scaffold2260	miRDeep2	389188	389299	62.6	rnafold:yes	ID=ID1165	Mature:ID1165-3P:scaffold2260:389258..389279:+	TCAAGTCCTTAGTTTCTCTTGC	-
scaffold2260	miRDeep2	365081	365192	10.1	rnafold:yes	ID=ID1167	Mature:ID1167-5P:scaffold2260:365101..365122:+	AGGGGAAGCTAGGGTACTCAGA	-
scaffold227	miRDeep2	34075	34186	1358.9	rnafold:yes	ID=ID1388	Mature:ID1388-3P:scaffold227:34095..34116:-	TTCGGCGATGAGATCAGCCAGT	tca-miR-6012
scaffold22856	miRDeep2	99030	99144	93.7	rnafold:yes	ID=ID390	Mature:ID390-3P:scaffold22856:99088..99109:+	TTACGTATCAGTTAAGTAATAG	-
scaffold22931	miRDeep2	44325	44437	40.9	rnafold:yes	ID=ID747	Mature:ID747-3P:scaffold22931:44345..44368:-	AGACGTGCCGGAAGTGCGGGTCAG	-
scaffold22931	miRDeep2	40419	40530	97.9	rnafold:yes	ID=ID748	Mature:ID748-3P:scaffold22931:40439..40460:-	TCAAGTGAGGTAACATTTTAGA	-
scaffold22931	miRDeep2	43471	43582	143.1	rnafold:yes	ID=ID753	Mature:ID753-3P:scaffold22931:43491..43512:-	ATTTGGTCCAGGCAAAAATGCT	-
scaffold22931	miRDeep2	39969	40079	97.2	rnafold:yes	ID=ID754	Mature:ID754-3P:scaffold22931:39989..40010:-	TGAAGCAGTAGAAGCCAAGACA	-
scaffold23128	miRDeep2	112734	112845	2613.2	rnafold:yes	ID=ID1361	Mature:ID1361-5P:scaffold23128:112754..112775:+	CTCAATGTCGCCTGTAATGCCT	-
scaffold23556	miRDeep2	9159	9270	2624.3	rnafold:yes	ID=ID212	Mature:ID212-5P:scaffold23556:9229..9250:-	CTCAATGTCGCCTGTAATGCCT	-

scaffold237	miRDeep2	55421	55535	15.6	rnafold:yes	ID=ID594	Mature:ID594-3P:scaffold237:55491..55515:+	CACGCAGTGACAATGACCTTCCCT	-
scaffold24256	miRDeep2	90471	90584	31.8	rnafold:yes	ID=ID680	Mature:ID680-5P:scaffold24256:90541..90564:-	TCTTGCAAGGGATATTGCTAGGGG	-
scaffold24716	miRDeep2	5319	5430	745.4	rnafold:yes	ID=ID808	Mature:ID808-3P:scaffold24716:5339..5360:-	CTCTGTCAAGCACTCAGAGGGG	-
scaffold24716	miRDeep2	18128	18238	2523.5	rnafold:yes	ID=ID809	Mature:ID809-3P:scaffold24716:18148..18169:-	CTTTATCCAGCACTCAGAGGGG	-
scaffold24716	miRDeep2	10331	10443	315242.8	rnafold:yes	ID=ID810	Mature:ID810-5P:scaffold24716:10401..10423:-	CGCTGAATGCTGGACAGAGGACT	-
scaffold248	miRDeep2	470423	470535	1010.2	rnafold:yes	ID=ID1747	Mature:ID1747-5P:scaffold248:470493..470516:-	CGGCTAACAGTAGTGCGCCCCGGC	-
scaffold249	miRDeep2	2E+06	2E+06	594	rnafold:yes	ID=ID890	Mature:ID890-3P:scaffold249:1599140..1599160:-	CTTGTACTGCGACGCTAACAA	-
scaffold249575	miRDeep2	4758	4870	48.8	rnafold:yes	ID=ID20	Mature:ID20-5P:scaffold249575:4792..4813:+	GGGGTGACATGTACCAGCAGTG	-
scaffold252751	miRDeep2	1025	1133	1549.1	rnafold:yes	ID=ID326	Mature:ID326-5P:scaffold252751:1095..1113:-	ACGTATACTGAATGTATCC	dvi-miR-iab
scaffold25339	miRDeep2	405919	406030	55.1	rnafold:yes	ID=ID656	Mature:ID656-5P:scaffold25339:405939..405961:+	TGGTCGACTTTCTTCTGAGGTGC	-
scaffold25647	miRDeep2	25668	25776	8488.1	rnafold:yes	ID=ID1529	Mature:ID1529-3P:scaffold25647:25738..25758:+	TGCAAGCAGTGCGGAAGTGAG	ame-miR-932
scaffold2603	miRDeep2	75043	75154	1756	rnafold:yes	ID=ID1393	Mature:ID1393-3P:scaffold2603:75063..75084:-	CATACTCAATATATGTTGCTCT	-
scaffold26048	miRDeep2	148244	148354	7246.5	rnafold:yes	ID=ID1074	Mature:ID1074-5P:scaffold26048:148314..148335:-	TTTTAGAATTTGTACGCTTTGT	tca-miR-927b
scaffold266	miRDeep2	217212	217320	46.3	rnafold:yes	ID=ID1576	Mature:ID1576-5P:scaffold266:217289..217310:-	GATATGGGTTTTAAGGAAGTGG	-
scaffold2708	miRDeep2	1E+06	1E+06	9196.6	rnafold:yes	ID=ID1594	Mature:ID1594-5P:scaffold2708:1250075..1250096:	TGAGACAGACAAAGGGAGAAAG	-
scaffold271461	miRDeep2	1254	1365	691.5	rnafold:yes	ID=ID1447	Mature:ID1447-3P:scaffold271461:1274..1295:-	GCGCGCCGGGGTTTGCTGCGTG	-
scaffold27290	miRDeep2	78967	79077	1522.7	rnafold:yes	ID=ID465	Mature:ID465-5P:scaffold27290:78989..79009:+	TCTGAGTGCTGGACAGAGGGC	-
scaffold27290	miRDeep2	128744	128855	196.7	rnafold:yes	ID=ID466	Mature:ID466-5P:scaffold27290:128764..128785:+	TTATTTGGCACTCATGGAATCG	-
scaffold27290	miRDeep2	110073	110184	884.4	rnafold:yes	ID=ID467	Mature:ID467-5P:scaffold27290:110093..110114:+	GTCTGGGTGCTGGACAGACCGC	-
scaffold27290	miRDeep2	111390	111499	1163.7	rnafold:yes	ID=ID468	Mature:ID468-5P:scaffold27290:111410..111430:+	TCCGCGTGTTGGACAGAGCGC	-
scaffold27290	miRDeep2	108525	108635	355.6	rnafold:yes	ID=ID469	Mature:ID469-5P:scaffold27290:108545..108565:+	CGTCTGTGTGCTGGGCAGAGC	-
scaffold27290	miRDeep2	131429	131540	5846.2	rnafold:yes	ID=ID470	Mature:ID470-3P:scaffold27290:131499..131520:+	CCTGCTCCAGCACTCAGAGGGG	-
scaffold27290	miRDeep2	104223	104335	18108.6	rnafold:yes	ID=ID471	Mature:ID471-5P:scaffold27290:104243..104265:+	GCTTTGAGTGCTGGACAGAGGGC	-
scaffold27290	miRDeep2	103648	103759	2530.6	rnafold:yes	ID=ID472	Mature:ID472-5P:scaffold27290:103668..103689:+	ATCAGGGTGCTGGACAGAGCGC	-
scaffold27290	miRDeep2	72287	72397	2062.2	rnafold:yes	ID=ID474	Mature:ID474-3P:scaffold27290:72357..72378:+	CTCTGTTCAGCACTCTGAGGGG	-

scaffold27290	miRDeep2	77140	77250	3161.8	rnafold:yes	ID=ID475	Mature:ID475-5P:scaffold27290:77160..77182:+	CGTCTGAGTGCTGGACAGAGGGC	-
scaffold27290	miRDeep2	101046	101158	117.4	rnafold:yes	ID=ID476	Mature:ID476-3P:scaffold27290:101101..101123:+	CCTCTGTCCAGCACTCAGAGGGG	-
scaffold27290	miRDeep2	101278	101389	196.4	rnafold:yes	ID=ID477	Mature:ID477-5P:scaffold27290:101298..101320:+	CGTCTGGGTGCTGGACAGATCAG	-
scaffold27290	miRDeep2	142546	142655	248.7	rnafold:yes	ID=ID478	Mature:ID478-3P:scaffold27290:142616..142637:+	AGCTGTCGGGCACGCAGAGGGA	-
scaffold2732	miRDeep2	596325	596435	5352	rnafold:yes	ID=ID589	Mature:ID589-3P:scaffold2732:596345..596367:-	GTACCCGGTCACCTCCTTCCTGA	-
scaffold27460	miRDeep2	86491	86602	92	rnafold:yes	ID=ID1082	Mature:ID1082-5P:scaffold27460:86514..86535:+	CACGGCCCACTCGCTGGAGCGC	-
scaffold2756	miRDeep2	1E+06	1E+06	183.7	rnafold:yes	ID=ID322	Mature:ID322-5P:scaffold2756:1033246..1033268:+	AGAAGGGCATCGACGGCAGCTTG	-
scaffold276129	miRDeep2	403	512	161.4	rnafold:yes	ID=ID1236	Mature:ID1236-5P:scaffold276129:423..444:+	AGAGTGTCAGGAATGTGACTCG	-
scaffold280175	miRDeep2	345	459	67.4	rnafold:yes	ID=ID546	Mature:ID546-5P:scaffold280175:365..385:+	TGGACTTTGATCGGGATAGGA	-
scaffold28606	miRDeep2	62662	62771	275.4	rnafold:yes	ID=ID1617	Mature:ID1617-3P:scaffold28606:62732..62753:+	CCAGAGGGCTGTGTGGTGACCG	-
scaffold28917	miRDeep2	17105	17215	202.4	rnafold:yes	ID=ID645	Mature:ID645-3P:scaffold28917:17175..17196:+	TGCTGCCCTGTGCAGAGGTTGA	-
scaffold28981	miRDeep2	86587	86699	7760.6	rnafold:yes	ID=ID904	Mature:ID904-3P:scaffold28981:86607..86629:-	TAGCTGCCTAGAGAAGGGCATCA	aae-miR-980
scaffold2935	miRDeep2	2E+06	2E+06	393	rnafold:yes	ID=ID595	Mature:ID595-5P:scaffold2935:1678222..1678245:+	TGGCAGGAAGTTTCGTTTTCTAGA	-
scaffold29372	miRDeep2	4094	4203	1381.4	rnafold:yes	ID=ID699	Mature:ID699-3P:scaffold29372:4164..4185:+	TTGAGATCTCGATTCCGTTACT	-
scaffold29372	miRDeep2	45	156	357.1	rnafold:yes	ID=ID700	Mature:ID700-5P:scaffold29372:79..103:+	TGACGGAAGCGAGGTCTGTACAAG	-
scaffold29372	miRDeep2	1841	1952	357.1	rnafold:yes	ID=ID702	Mature:ID702-5P:scaffold29372:1875..1899:+	TGACGGAAGCGAGGTCTGTACAAG	-
scaffold29372	miRDeep2	6658	6767	1400.7	rnafold:yes	ID=ID703	Mature:ID703-3P:scaffold29372:6728..6749:+	TTGAGATCTCGATTCCGTTACT	-
scaffold29670	miRDeep2	102219	102330	1822.9	rnafold:yes	ID=ID729	Mature:ID729-3P:scaffold29670:102239..102261:-	CATCACATTTGTATTGTCTCACA	-
scaffold29670	miRDeep2	49955	50065	5823.3	rnafold:yes	ID=ID730	Mature:ID730-3P:scaffold29670:49975..49996:-	TACATCATAGGTGTGCGGGTGT	-
scaffold29670	miRDeep2	63035	63146	13605.2	rnafold:yes	ID=ID731	Mature:ID731-3P:scaffold29670:63055..63077:-	CATCACATTTGTATGGTCTCATA	-
scaffold29676	miRDeep2	77062	77171	65.4	rnafold:yes	ID=ID369	Mature:ID369-5P:scaffold29676:77081..77102:+	CTGGGCGGACATACTGTAGAAA	-
scaffold297269	miRDeep2	1243	1350	12228.6	rnafold:yes	ID=ID885	Mature:ID885-3P:scaffold297269:1313..1333:+	ACAGAAATTGTGTATTGTCGA	-
scaffold2978	miRDeep2	4840	4951	36.4	rnafold:yes	ID=ID335	Mature:ID335-3P:scaffold2978:4910..4931:+	TGAACTGGCAGTGCACCTGTGG	-
scaffold29860	miRDeep2	79004	79113	152076.8	rnafold:yes	ID=ID1725	Mature:ID1725-5P:scaffold29860:79024..79045:+	TGAAAGACATGGGTAGTGAGAT	bmo-miR-71
scaffold29860	miRDeep2	79153	79263	8881.1	rnafold:yes	ID=ID1726	Mature:ID1726-3P:scaffold29860:79223..79245:+	TATCACAGCCAGCTTTGATGAGC	bmo-miR-2a

scaffold29860	miRDeep2	79691	79801	110893.4	rnafold:yes	ID=ID1727	Mature:ID1727-3P:scaffold29860:79761..79784:+	TATCACAGCCATTTTTGACGAGTT	aae-miR-13
scaffold29860	miRDeep2	79938	80048	10095.2	rnafold:yes	ID=ID1728	Mature:ID1728-3P:scaffold29860:80008..80030:+	TATCACAGCCAGCTTTGATGAGC	bmo-miR-13a
scaffold29860	miRDeep2	79816	79926	15744.6	rnafold:yes	ID=ID1729	Mature:ID1729-3P:scaffold29860:79886..79906:+	TATCACAGCCAGCTTTGATGA	bmo-miR-13a
scaffold29860	miRDeep2	79278	79387	89749.4	rnafold:yes	ID=ID1730	Mature:ID1730-3P:scaffold29860:79348..79370:+	TATCACAGCCACTTTGATGAGCT	dme-miR-2c
scaffold2993	miRDeep2	196834	196945	58.9	rnafold:yes	ID=ID262	Mature:ID262-5P:scaffold2993:196854..196876:+	CGCAGAATCCGACGGTGACGTCC	-
scaffold30145	miRDeep2	47780	47894	3652	rnafold:yes	ID=ID1468	Mature:ID1468-5P:scaffold30145:47850..47874:-	AACGATGGTACTGTACAGTATGTG	-
scaffold3024	miRDeep2	1E+06	1E+06	642	rnafold:yes	ID=ID1754	Mature:ID1754-3P:scaffold3024:1322886..1322907:-	TCGGCACAGAGTGAGGTGATGG	-
scaffold3030	miRDeep2	123207	123318	1475.4	rnafold:yes	ID=ID167	Mature:ID167-5P:scaffold3030:123227..123248:+	GTGAGAAGATAGCACTCTAGTT	-
scaffold3030	miRDeep2	129185	129294	17101	rnafold:yes	ID=ID168	Mature:ID168-3P:scaffold3030:129255..129277:+	TGTAGAGCGAAGTTTTCTCACGG	-
scaffold3030	miRDeep2	128599	128706	3885.8	rnafold:yes	ID=ID169	Mature:ID169-5P:scaffold3030:128631..128653:+	GTGAGAAAGCGGCATTCTAGTTG	-
scaffold3030	miRDeep2	141435	141546	15373.9	rnafold:yes	ID=ID170	Mature:ID170-5P:scaffold3030:141455..141476:+	GTGAAAAAGTAGCACTCTAGTT	-
scaffold3030	miRDeep2	108241	108349	7691.1	rnafold:yes	ID=ID171	Mature:ID171-3P:scaffold3030:108311..108333:+	CTAGTGTGAAGCTTTCTCACATT	-
scaffold30454	miRDeep2	58614	58724	97.2	rnafold:yes	ID=ID537	Mature:ID537-3P:scaffold30454:58634..58655:-	TGAAGCAGTAGAAGCCAAGACA	-
scaffold30454	miRDeep2	62147	62258	143.1	rnafold:yes	ID=ID539	Mature:ID539-3P:scaffold30454:62167..62188:-	ATTTGGTCCAGGCAAAAATGCT	-
scaffold30454	miRDeep2	59051	59162	97.9	rnafold:yes	ID=ID541	Mature:ID541-3P:scaffold30454:59071..59092:-	TCAAGTGAGGTAACATTTTAGA	-
scaffold30454	miRDeep2	63201	63313	40.9	rnafold:yes	ID=ID542	Mature:ID542-3P:scaffold30454:63221..63244:-	AGACGTGCCGGAAGTGCGGGTCAG	-
scaffold3052	miRDeep2	69318	69427	6718.9	rnafold:yes	ID=ID323	Mature:ID323-3P:scaffold3052:69388..69409:+	ATCCCCTGGTGAAACTTTCCGC	-
scaffold305423	miRDeep2	379	490	602.5	rnafold:yes	ID=ID1284	Mature:ID1284-3P:scaffold305423:449..470:+	GGGGAGTCGGAACAATGGCAGC	-
scaffold3080	miRDeep2	409146	409257	132.1	rnafold:yes	ID=ID1589	Mature:ID1589-5P:scaffold3080:409216..409237:-	ACTGTTCATGTGGAGAGCTCCT	-
scaffold3104	miRDeep2	764371	764483	46.2	rnafold:yes	ID=ID1312	Mature:ID1312-3P:scaffold3104:764392..764413:-	AGGTCGAAACCGGTCACCTTAT	-
scaffold3107	miRDeep2	773827	773937	19048.7	rnafold:yes	ID=ID1766	Mature:ID1766-5P:scaffold3107:773860..773881:+	TGCTGGACACTGAATACAGCAG	-
scaffold3107	miRDeep2	712549	712660	27248.8	rnafold:yes	ID=ID1767	Mature:ID1767-5P:scaffold3107:712569..712590:+	TGCTGGACACTGAATACAGCGG	-
scaffold3107	miRDeep2	773825	773936	395.1	rnafold:yes	ID=ID1768	Mature:ID1768-5P:scaffold3107:773895..773916:-	CACTGGACACTGCCTACAGCAG	-
scaffold3107	miRDeep2	699969	700078	1441.5	rnafold:yes	ID=ID1769	Mature:ID1769-5P:scaffold3107:700009..700031:+	TGCTGGACACTGAATACAGCAGT	-
scaffold3107	miRDeep2	701457	701566	1441.5	rnafold:yes	ID=ID1770	Mature:ID1770-5P:scaffold3107:701497..701519:+	TGCTGGACACTGAATACAGCAGT	-

scaffold3107	miRDeep2	714840	714951	27248.8	rnafold:yes	ID=ID1771	Mature:ID1771-5P:scaffold3107:714860..714881:+	TGCTGGACACTGAATACAGCGG	-
scaffold3107	miRDeep2	712177	712286	1441.5	rnafold:yes	ID=ID1772	Mature:ID1772-5P:scaffold3107:712217..712239:+	TGCTGGACACTGAATACAGCAGT	-
scaffold3107	miRDeep2	749971	750082	27292.5	rnafold:yes	ID=ID1773	Mature:ID1773-5P:scaffold3107:749991..750012:+	TGCTGGACACTGAATACAGCGG	-
scaffold3107	miRDeep2	712901	713012	27248.8	rnafold:yes	ID=ID1774	Mature:ID1774-5P:scaffold3107:712921..712942:+	TGCTGGACACTGAATACAGCGG	-
scaffold3107	miRDeep2	682717	682828	26857.4	rnafold:yes	ID=ID1775	Mature:ID1775-5P:scaffold3107:682737..682758:+	TGCTGGACACTGAATACAGCGG	-
scaffold31186	miRDeep2	5758	5870	67.5	rnafold:yes	ID=ID1621	Mature:ID1621-5P:scaffold31186:5828..5850:-	CCACTGCAGAACCCTCAAACTCT	-
scaffold312	miRDeep2	531902	532013	35.5	rnafold:yes	ID=ID277	Mature:ID277-5P:scaffold312:531972..531993:-	AGAATCAGTAGTTGCTGAGTCA	-
scaffold312	miRDeep2	529510	529621	21.7	rnafold:yes	ID=ID278	Mature:ID278-5P:scaffold312:529580..529601:-	AGAATCAGTAGTTGCTGAGTCA	-
scaffold3120	miRDeep2	1E+06	1E+06	3732.3	rnafold:yes	ID=ID1095	Mature:ID1095-5P:scaffold3120:1167720..1167742:	CGGGTGAAGCTATAGCGATTATG	dpu-miR-965
scaffold313105	miRDeep2	167	276	1751.9	rnafold:yes	ID=ID1290	Mature:ID1290-3P:scaffold313105:237..258:+	GAGGAGTCGGAACAATGGCAGC	-
scaffold313453	miRDeep2	4308	4419	62.2	rnafold:yes	ID=ID501	Mature:ID501-3P:scaffold313453:4329..4351:-	ACCACTGGTGATGCGTCTCAACG	-
scaffold3162	miRDeep2	2E+06	2E+06	4188.8	rnafold:yes	ID=ID1402	Mature:ID1402-3P:scaffold3162:1630531..1630552:	TAGGACGGTCCTTATGACACCT	-
scaffold3162	miRDeep2	2E+06	2E+06	4284	rnafold:yes	ID=ID1403	Mature:ID1403-5P:scaffold3162:1535109..1535131:	AGGGTGTGGCTAGTAATCGACAT	-
scaffold3162	miRDeep2	1E+06	1E+06	116.6	rnafold:yes	ID=ID1404	Mature:ID1404-5P:scaffold3162:1467297..1467319:	AGAAGAACATCAGCTGGCTTACA	-
scaffold3162	miRDeep2	2E+06	2E+06	15	rnafold:yes	ID=ID1405	Mature:ID1405-5P:scaffold3162:1655221..1655240:	CGTCATAAGGGCAGTGAGCA	-
scaffold3162	miRDeep2	2E+06	2E+06	764.7	rnafold:yes	ID=ID1406	Mature:ID1406-5P:scaffold3162:1529862..1529881:	GGGGGAAATTGACTGGTGAG	-
scaffold3162	miRDeep2	2E+06	2E+06	39.9	rnafold:yes	ID=ID1407	Mature:ID1407-3P:scaffold3162:1603683..1603704:	TCTTTGGTCTCTTACGACACCT	-
scaffold3162	miRDeep2	2E+06	2E+06	946.1	rnafold:yes	ID=ID1408	Mature:ID1408-3P:scaffold3162:1546281..1546302:	CTTGTAATTCCATTGTTGTTCG	-
scaffold3162	miRDeep2	2E+06	2E+06	562.6	rnafold:yes	ID=ID1409	Mature:ID1409-5P:scaffold3162:1540715..1540736:	TGACAACAGTTGGTGTAGAAAC	-
scaffold3162	miRDeep2	2E+06	2E+06	78.2	rnafold:yes	ID=ID1410	Mature:ID1410-3P:scaffold3162:1621068..1621088:	GACGCTGGTTACTTGTGGCAG	-
scaffold3162	miRDeep2	2E+06	2E+06	14553.8	rnafold:yes	ID=ID1411	Mature:ID1411-3P:scaffold3162:1654774..1654794:	CACTCTGCCCCTATGACGCCT	-
scaffold3162	miRDeep2	2E+06	2E+06	54317.3	rnafold:yes	ID=ID1412	Mature:ID1412-5P:scaffold3162:1647393..1647414:	AAATAGGACCAGACGGAACACA	-
scaffold3162	miRDeep2	2E+06	2E+06	2255.4	rnafold:yes	ID=ID1413	Mature:ID1413-3P:scaffold3162:1658436..1658456:	CCCATTGACCTTGTGACGCCT	-
scaffold3162	miRDeep2	2E+06	2E+06	14553.7	rnafold:yes	ID=ID1414	Mature:ID1414-3P:scaffold3162:1658677..1658697:	CACTCTGCCCCTATGACGCCT	-
scaffold3162	miRDeep2	2E+06	2E+06	868.4	rnafold:yes	ID=ID1415	Mature:ID1415-3P:scaffold3162:1655501..1655521:	CATTCTGCCCCTATGACGCCT	-

scaffold3162	miRDeep2	2E+06	2E+06	5441.6	rnafold:yes	ID=ID1416	Mature:ID1416-3P:scaffold3162:1656824..1656844:	CACTCTGCTCCTATGACGCCT	-
scaffold3162	miRDeep2	2E+06	2E+06	34.5	rnafold:yes	ID=ID1418	Mature:ID1418-3P:scaffold3162:1540491..1540512:	TTGGTGCTGCCACTGTTGGCGG	-
scaffold3162	miRDeep2	2E+06	2E+06	23541.7	rnafold:yes	ID=ID1419	Mature:ID1419-3P:scaffold3162:1655981..1656003:	TGGACACTGGCCTTATGACGCCT	-
scaffold3162	miRDeep2	2E+06	2E+06	30.6	rnafold:yes	ID=ID1420	Mature:ID1420-3P:scaffold3162:1547174..1547195:	GTTGTACTTCCTTTGATGCTCG	-
scaffold3162	miRDeep2	2E+06	2E+06	150.2	rnafold:yes	ID=ID1421	Mature:ID1421-3P:scaffold3162:1651752..1651773:	TGTCTCGTCGCTTATGACGCCT	-
scaffold3162	miRDeep2	2E+06	2E+06	250.4	rnafold:yes	ID=ID1422	Mature:ID1422-5P:scaffold3162:1541512..1541533:	AGTGGCAATGGTTTTACACACT	-
scaffold3162	miRDeep2	2E+06	2E+06	3905.2	rnafold:yes	ID=ID1423	Mature:ID1423-3P:scaffold3162:1629680..1629701:	TAGCTCTGGACTTATGACGCCT	-
scaffold3162	miRDeep2	2E+06	2E+06	838.5	rnafold:yes	ID=ID1424	Mature:ID1424-3P:scaffold3162:1658917..1658939:	TGCGCATTGACCTTATGACACCT	-
scaffold3162	miRDeep2	2E+06	2E+06	49.4	rnafold:yes	ID=ID1425	Mature:ID1425-5P:scaffold3162:1534309..1534330:	AAAAGAAAATGGCTGTTTAACA	-
scaffold3162	miRDeep2	2E+06	2E+06	5441.6	rnafold:yes	ID=ID1426	Mature:ID1426-3P:scaffold3162:1657065..1657085:	CACTCTGCTCCTATGACGCCT	-
scaffold3162	miRDeep2	2E+06	2E+06	840.5	rnafold:yes	ID=ID1427	Mature:ID1427-3P:scaffold3162:1655742..1655762:	CATTCTGCCCCTATGACGCCT	-
scaffold3162	miRDeep2	2E+06	2E+06	121.9	rnafold:yes	ID=ID1428	Mature:ID1428-5P:scaffold3162:1521640..1521663:	AGGAGAGACGCAGCAGTGGAACAT	-
scaffold3162	miRDeep2	1E+06	1E+06	170358.6	rnafold:yes	ID=ID1430	Mature:ID1430-5P:scaffold3162:1474781..1474803:	AGAAGTGCAGTAGTTGACGAACA	-
scaffold3162	miRDeep2	2E+06	2E+06	3802.4	rnafold:yes	ID=ID1431	Mature:ID1431-3P:scaffold3162:1699492..1699513:	TGGTTGCATGCTTATGACGTCA	-
scaffold3162	miRDeep2	2E+06	2E+06	320.4	rnafold:yes	ID=ID1432	Mature:ID1432-3P:scaffold3162:1655014..1655034:	TTCATTGGCCTTGTGACACCT	-
scaffold3162	miRDeep2	2E+06	2E+06	34.4	rnafold:yes	ID=ID1435	Mature:ID1435-3P:scaffold3162:1637484..1637505:	TCTTTGGCCTCTTATGACTCCT	-
scaffold31857	miRDeep2	2558	2669	28.1	rnafold:yes	ID=ID7	Mature:ID7-3P:scaffold31857:2628..2649:+	TGACTTTCGCTACTCCATCTAG	-
scaffold31873	miRDeep2	145399	145510	68110	rnafold:yes	ID=ID603	Mature:ID603-3P:scaffold31873:145419..145440:-	CCAGGTAACAGATCCTCGACCG	-
scaffold31873	miRDeep2	91324	91435	47941	rnafold:yes	ID=ID604	Mature:ID604-3P:scaffold31873:91344..91365:-	TAGGTAACAGAACCCCGAGTGT	-
scaffold31873	miRDeep2	121109	121220	68109.9	rnafold:yes	ID=ID605	Mature:ID605-3P:scaffold31873:121129..121150:-	CCAGGTAACAGATCCTCGACCG	-
scaffold31873	miRDeep2	90580	90691	50929.4	rnafold:yes	ID=ID607	Mature:ID607-3P:scaffold31873:90600..90621:-	TAGGTAACAGAACCCCGAGTGT	-
scaffold31873	miRDeep2	91121	91232	47304.2	rnafold:yes	ID=ID608	Mature:ID608-3P:scaffold31873:91141..91162:-	TAGGTAACAGAACCCCGAGTGT	-
scaffold31873	miRDeep2	102838	102949	8774.6	rnafold:yes	ID=ID609	Mature:ID609-5P:scaffold31873:102908..102929:-	TGGTCGGGTATCTGTGCCTTGT	-
scaffold31873	miRDeep2	90923	91034	47940.9	rnafold:yes	ID=ID610	Mature:ID610-3P:scaffold31873:90943..90964:-	TAGGTAACAGAACCCCGAGTGT	-
scaffold31873	miRDeep2	101838	101949	944.2	rnafold:yes	ID=ID611	Mature:ID611-5P:scaffold31873:101908..101929:-	TGTTATTCTGATTGACAGCTGT	-

scaffold32024	miRDeep2	22325	22435	23715.7	rnafold:yes	ID=ID873	Mature:ID873-5P:scaffold32024:22345..22365:+	CGTTAATTTCATGTGCAAGCA	-
scaffold328365	miRDeep2	868	977	758	rnafold:yes	ID=ID1088	Mature:ID1088-3P:scaffold328365:938..960:+	TTTAAGAGGCACTTCTGACGACT	-
scaffold328365	miRDeep2	1351	1459	405.4	rnafold:yes	ID=ID1089	Mature:ID1089-3P:scaffold328365:1421..1442:+	TTGATGAGGCACTTCTAACGAT	-
scaffold3305	miRDeep2	674564	674671	1999.8	rnafold:yes	ID=ID1068	Mature:ID1068-3P:scaffold3305:674634..674655:+	AGATGCCGAGTGGTTAAGGCGA	-
scaffold3305	miRDeep2	677301	677409	100675.6	rnafold:yes	ID=ID1069	Mature:ID1069-5P:scaffold3305:677321..677342:+	CTAAGTACTAGTGCCGCAGGAG	bmo-miR-252
scaffold3306	miRDeep2	271600	271711	101080.5	rnafold:yes	ID=ID1269	Mature:ID1269-5P:scaffold3306:271670..271691:-	TGGTAACTCCACCACCGTTGGC	bmo-miR-2765
scaffold33260	miRDeep2	205872	205982	196405.5	rnafold:yes	ID=ID1378	Mature:ID1378-3P:scaffold33260:205942..205963:+	ATGAGCAATGTTATTCAAATGG	-
scaffold332781	miRDeep2	486	598	10095.1	rnafold:yes	ID=ID903	Mature:ID903-3P:scaffold332781:506..528:-	TATCACAGCCAGCTTTGATGAGC	bmo-miR-13a
scaffold338065	miRDeep2	16	127	50.1	rnafold:yes	ID=ID1277	Mature:ID1277-3P:scaffold338065:86..107:+	ATATACATAATCGGTGCTTTGT	-
scaffold34243	miRDeep2	69289	69401	36.7	rnafold:yes	ID=ID1381	Mature:ID1381-3P:scaffold34243:69311..69331:-	TCACTGGCACGATTCCATCAT	-
scaffold350968	miRDeep2	5126	5238	48.8	rnafold:yes	ID=ID1646	Mature:ID1646-5P:scaffold350968:5160..5181:+	GGGGTGACATGTACCAGCAGTG	-
scaffold351	miRDeep2	2E+06	2E+06	59.1	rnafold:yes	ID=ID724	Mature:ID724-5P:scaffold351:1738088..1738111:+	CATTGTGATGATAATGCTTTGGTC	-
scaffold3515	miRDeep2	316994	317101	820086.6	rnafold:yes	ID=ID388	Mature:ID388-3P:scaffold3515:317064..317086:+	TGAGATCATTGTGAAAGCTGATT	aga-bantam
scaffold35332	miRDeep2	118693	118804	73435.3	rnafold:yes	ID=ID698	Mature:ID698-5P:scaffold35332:118763..118784:-	AGGACAATGACGATGACGATGA	-
scaffold353623	miRDeep2	1220	1331	449	rnafold:yes	ID=ID1535	Mature:ID1535-5P:scaffold353623:1293..1315:-	CATCTGAGTGCTGGACAGAGGGC	-
scaffold35765	miRDeep2	259412	259521	12584.7	rnafold:yes	ID=ID1623	Mature:ID1623-5P:scaffold35765:259432..259453:+	AACTCTGTGGTGCTAGTGACAG	-
scaffold358752	miRDeep2	1046	1154	34632.2	rnafold:yes	ID=ID830	Mature:ID830-5P:scaffold358752:1066..1087:+	TGCTGGATACTGAATACAGCGG	-
scaffold361561	miRDeep2	510	622	115.5	rnafold:yes	ID=ID1591	Mature:ID1591-3P:scaffold361561:580..602:+	GATCTACATAATCGGTGCTGTGT	-
scaffold361561	miRDeep2	959	1071	115.5	rnafold:yes	ID=ID1592	Mature:ID1592-3P:scaffold361561:1029..1051:+	GATCTACATAATCGGTGCTGTGT	-
scaffold3625	miRDeep2	2E+06	2E+06	15183.2	rnafold:yes	ID=ID1314	Mature:ID1314-3P:scaffold3625:1883815..1883835:	GCAGGAGTAGGCAGTATCACG	-
scaffold363497	miRDeep2	575	685	1203.9	rnafold:yes	ID=ID685	Mature:ID685-3P:scaffold363497:595..615:-	TTATTGCTTGAGAATACACGT	tca-miR-137
scaffold3755	miRDeep2	139085	139196	9680.4	rnafold:yes	ID=ID641	Mature:ID641-5P:scaffold3755:139155..139176:-	CGTGTCGGCATGTTGAGACGCG	-
scaffold38493	miRDeep2	9497	9604	94139.4	rnafold:yes	ID=ID592	Mature:ID592-3P:scaffold38493:9567..9588:+	AAGATCTAGTAGAGACGGCTGC	-
scaffold38493	miRDeep2	267	376	1164	rnafold:yes	ID=ID593	Mature:ID593-5P:scaffold38493:287..307:+	TTGATTATGGGCCGAAGCATT	-
scaffold385215	miRDeep2	655	764	32.7	rnafold:yes	ID=ID266	Mature:ID266-5P:scaffold385215:727..751:-	TTAGTATAGTATAGGTGGGAGTT	-

scaffold3857	miRDeep2	1E+06	1E+06	207.3	rnafold:yes	ID=ID511	Mature:ID511-3P:scaffold3857:1427536..1427559:-	AAAACGTCGTAGCTGCTTTGGCAC	-
scaffold3857	miRDeep2	1E+06	1E+06	1380.6	rnafold:yes	ID=ID513	Mature:ID513-3P:scaffold3857:1438947..1438971:-	TCAAACGTCATAGCTGCTTTGGTA	-
scaffold3857	miRDeep2	1E+06	1E+06	14970.9	rnafold:yes	ID=ID514	Mature:ID514-3P:scaffold3857:1434487..1434511:-	TCAATACGTGTAGCTGCTTTGGAA	-
scaffold3857	miRDeep2	2E+06	2E+06	2255.2	rnafold:yes	ID=ID515	Mature:ID515-5P:scaffold3857:1637306..1637327:-	AAAAGCACCGACGAATTTACCT	-
scaffold3857	miRDeep2	2E+06	2E+06	2371.2	rnafold:yes	ID=ID516	Mature:ID516-3P:scaffold3857:1563097..1563118:-	CAGCATAAGACAGATTACTTCT	-
scaffold3857	miRDeep2	1E+06	1E+06	1380.5	rnafold:yes	ID=ID517	Mature:ID517-3P:scaffold3857:1439348..1439372:-	TCAAACGTCATAGCTGCTTTGGTA	-
scaffold3857	miRDeep2	1E+06	1E+06	187.1	rnafold:yes	ID=ID518	Mature:ID518-3P:scaffold3857:1434890..1434911:-	AAAACGTCGTAGCTGCTTTGGA	-
scaffold3857	miRDeep2	2E+06	2E+06	11.5	rnafold:yes	ID=ID520	Mature:ID520-5P:scaffold3857:1621422..1621443:-	TTCTCGAGTTTCCATCCGAGTG	-
scaffold3857	miRDeep2	2E+06	2E+06	5941.1	rnafold:yes	ID=ID521	Mature:ID521-5P:scaffold3857:1574631..1574653:-	TCTGGACCAGCCATTGCTCGTCA	-
scaffold3857	miRDeep2	1E+06	1E+06	55474.3	rnafold:yes	ID=ID522	Mature:ID522-3P:scaffold3857:1440332..1440355:-	TCAAATCGTCGTAGCTGCTTTGGC	-
scaffold3857	miRDeep2	1E+06	1E+06	1570.7	rnafold:yes	ID=ID523	Mature:ID523-3P:scaffold3857:1422249..1422272:-	AAAACGTCGTAGCTGCTTTGGAAC	-
scaffold3857	miRDeep2	1E+06	1E+06	2785.5	rnafold:yes	ID=ID524	Mature:ID524-5P:scaffold3857:1424310..1424331:-	TCCGGTGGCAATGACGGATTGA	-
scaffold3857	miRDeep2	1E+06	1E+06	3412.3	rnafold:yes	ID=ID525	Mature:ID525-3P:scaffold3857:1428441..1428463:-	TCAATACACGTAGCTGCTTTGGA	-
scaffold3857	miRDeep2	2E+06	2E+06	11.5	rnafold:yes	ID=ID526	Mature:ID526-5P:scaffold3857:1619547..1619568:+	TTCTCGAGTTTCCATCCGAGTG	-
scaffold3857	miRDeep2	1E+06	1E+06	2234	rnafold:yes	ID=ID527	Mature:ID527-5P:scaffold3857:1420273..1420294:-	TCCGGTGGCAATGACGGATTGA	-
scaffold3857	miRDeep2	2E+06	2E+06	63671.5	rnafold:yes	ID=ID528	Mature:ID528-5P:scaffold3857:1574460..1574483:-	TCTTCTGATGAGTGTGCTGTGGGT	-
scaffold3857	miRDeep2	1E+06	1E+06	2065.8	rnafold:yes	ID=ID529	Mature:ID529-5P:scaffold3857:1420672..1420693:-	TCCGGTGGCAATGACGGATTGA	-
scaffold3857	miRDeep2	1E+06	1E+06	6703.9	rnafold:yes	ID=ID530	Mature:ID530-5P:scaffold3857:1421512..1421533:-	CAGTGGCAATGACGGATTGATG	-
scaffold3857	miRDeep2	2E+06	2E+06	55474.3	rnafold:yes	ID=ID531	Mature:ID531-3P:scaffold3857:1650005..1650028:-	TCAAATCGTCGTAGCTGCTTTGGC	-
scaffold3857	miRDeep2	1E+06	1E+06	53.5	rnafold:yes	ID=ID533	Mature:ID533-5P:scaffold3857:1423326..1423347:-	CCGGTGGCAATGACGGATTGAT	-
scaffold3857	miRDeep2	1E+06	1E+06	6849.8	rnafold:yes	ID=ID534	Mature:ID534-5P:scaffold3857:1419870..1419891:-	CAGTGGCAATGACGGATTGATG	-
scaffold3951	miRDeep2	518351	518462	284.7	rnafold:yes	ID=ID427	Mature:ID427-3P:scaffold3951:518371..518393:-	CAGGTCACCAACAAGTCTGCTAG	-
scaffold3951	miRDeep2	507338	507450	207.8	rnafold:yes	ID=ID428	Mature:ID428-5P:scaffold3951:507408..507431:-	AGCTGACTTGTTGGCTGATTCGAA	-
scaffold3951	miRDeep2	523261	523375	2705.2	rnafold:yes	ID=ID429	Mature:ID429-5P:scaffold3951:523331..523355:-	AGCTGACTTGTTGGCTGATTCGAG	-
scaffold3951	miRDeep2	511175	511286	10.4	rnafold:yes	ID=ID430	Mature:ID430-3P:scaffold3951:511195..511217:-	CAGGTCACCAACAAGTCTGCCAG	-

scaffold3951	miRDeep2	545383	545494	284.7	rnafold:yes	ID=ID431	Mature:ID431-3P:scaffold3951:545403..545425:-	CAGGTCACCAACAAGTCTGCTAG	-
scaffold395457	miRDeep2	2179	2289	8488.1	rnafold:yes	ID=ID128	Mature:ID128-3P:scaffold395457:2199..2219:-	TGCAAGCAGTGCGGAAGTGAG	ame-miR-932
scaffold39789	miRDeep2	45642	45753	60628.6	rnafold:yes	ID=ID1507	Mature:ID1507-3P:scaffold39789:45662..45683:-	TGACTAGAGTGACACTCGTCAA	-
scaffold3992	miRDeep2	4157	4268	3161.8	rnafold:yes	ID=ID200	Mature:ID200-5P:scaffold3992:4227..4249:-	CGTCTGAGTGCTGGACAGAGGGC	-
scaffold40211	miRDeep2	15395	15502	1378.4	rnafold:yes	ID=ID799	Mature:ID799-3P:scaffold40211:15465..15486:+	CAATGCCCTTGGAAATCCCAAA	ame-miR-2788
scaffold40397	miRDeep2	7209	7321	1214.4	rnafold:yes	ID=ID351	Mature:ID351-3P:scaffold40397:7232..7253:-	TAGCTCATAGTCCCACACTCGG	-
scaffold40397	miRDeep2	15552	15663	1486.7	rnafold:yes	ID=ID352	Mature:ID352-3P:scaffold40397:15572..15594:-	CAGCTCGTAGTCCCACACTCGGA	-
scaffold40397	miRDeep2	14430	14542	1214.4	rnafold:yes	ID=ID353	Mature:ID353-3P:scaffold40397:14453..14474:-	TAGCTCATAGTCCCACACTCGG	-
scaffold40397	miRDeep2	6759	6870	1486.7	rnafold:yes	ID=ID354	Mature:ID354-3P:scaffold40397:6779..6801:-	CAGCTCGTAGTCCCACACTCGGA	-
scaffold40397	miRDeep2	5245	5355	15922.8	rnafold:yes	ID=ID355	Mature:ID355-5P:scaffold40397:5315..5336:-	TCAAGTGTGCTGGACAGAGGAC	-
scaffold40397	miRDeep2	15100	15211	1486.7	rnafold:yes	ID=ID356	Mature:ID356-3P:scaffold40397:15120..15142:-	CAGCTCGTAGTCCCACACTCGGA	-
scaffold40397	miRDeep2	13981	14092	89.3	rnafold:yes	ID=ID357	Mature:ID357-5P:scaffold40397:14038..14059:-	GAGTGCTGGACTGAGGGCCGGA	-
scaffold40397	miRDeep2	14649	14760	89.3	rnafold:yes	ID=ID358	Mature:ID358-5P:scaffold40397:14706..14727:-	GAGTGCTGGACTGAGGGCCGGA	-
scaffold4177	miRDeep2	890918	891031	19.5	rnafold:yes	ID=ID424	Mature:ID424-3P:scaffold4177:890988..891011:+	GCCTGAATAGGGGAACAAACATTT	-
scaffold4177	miRDeep2	879959	880072	150	rnafold:yes	ID=ID425	Mature:ID425-3P:scaffold4177:880029..880052:+	TGGATAAGGAAAACATGAGCATCT	-
scaffold4181	miRDeep2	1E+06	1E+06	25.6	rnafold:yes	ID=ID1244	Mature:ID1244-3P:scaffold4181:1112960..1112981:	TCACAGCTCCATAACGGTGCAT	-
scaffold4239	miRDeep2	1E+06	1E+06	185.9	rnafold:yes	ID=ID1264	Mature:ID1264-3P:scaffold4239:1464925..1464947:	TTTGTCACCGGCCGTCGTGAACT	-
scaffold43011	miRDeep2	636	743	147.8	rnafold:yes	ID=ID813	Mature:ID813-5P:scaffold43011:706..724:-	TATCCCGGACAAGCCCCCA	-
scaffold4366	miRDeep2	442587	442698	6761.8	rnafold:yes	ID=ID1281	Mature:ID1281-3P:scaffold4366:442607..442628:-	TCGCTATTATCTGTGACGATAT	-
scaffold4366	miRDeep2	444755	444866	15067.3	rnafold:yes	ID=ID1283	Mature:ID1283-3P:scaffold4366:444775..444796:-	TTGCCAGTAGGTGTGACAGAGA	-
scaffold4411	miRDeep2	743906	744016	54.4	rnafold:yes	ID=ID220	Mature:ID220-5P:scaffold4411:743926..743947:+	TGTAATACCAGCGCCTGTCTTC	-
scaffold4428	miRDeep2	1E+06	1E+06	4900.7	rnafold:yes	ID=ID1386	Mature:ID1386-5P:scaffold4428:1461287..1461309:	AGGCAAGATGTCGGCATAGCTGA	dvi-miR-31b
scaffold4428	miRDeep2	1E+06	1E+06	2947.6	rnafold:yes	ID=ID1387	Mature:ID1387-5P:scaffold4428:1037519..1037542:-	CGGAATTTGTCTGTCCTGTCTGGT	-
scaffold4458	miRDeep2	141637	141746	13443.1	rnafold:yes	ID=ID814	Mature:ID814-5P:scaffold4458:141657..141678:+	TCTCGGACGAGGGCAGGTTCAG	-
scaffold45188	miRDeep2	81588	81698	14.1	rnafold:yes	ID=ID851	Mature:ID851-3P:scaffold45188:81608..81628:-	CGGCGTTTGCCATAGCTTGCG	-

scaffold45248	miRDeep2	5416	5527	10.9	rnafold:yes	ID=ID1742	Mature:ID1742-3P:scaffold45248:5473..5495:+	TCAGTGGTTGAACTACAACTATT	-
scaffold457	miRDeep2	5E+06	5E+06	240.1	rnafold:yes	ID=ID649	Mature:ID649-5P:scaffold457:5470081..5470102:-	AGACAGTCGTGGTTGCAACTTT	-
scaffold4605	miRDeep2	518597	518709	17.2	rnafold:yes	ID=ID1614	Mature:ID1614-3P:scaffold4605:518648..518672:+	TCCCGTCGAAGGTCAAAGCAGGGT	-
scaffold4715	miRDeep2	232635	232743	4629.2	rnafold:yes	ID=ID75	Mature:ID75-5P:scaffold4715:232655..232677:+	CGGAGGAAACCTACACGAACACG	-
scaffold4715	miRDeep2	233158	233270	3707.9	rnafold:yes	ID=ID76	Mature:ID76-5P:scaffold4715:233178..233200:+	CGGTGGAAACCTACACGAACACG	-
scaffold4715	miRDeep2	233572	233680	3045.7	rnafold:yes	ID=ID78	Mature:ID78-5P:scaffold4715:233592..233614:+	CGGTGGAAACCTACACGAACACG	-
scaffold4777	miRDeep2	16259	16371	7286	rnafold:yes	ID=ID306	Mature:ID306-3P:scaffold4777:16315..16336:+	TATTGCACTTGTCCCGGCCTAT	api-miR-92b
scaffold47839	miRDeep2	31873	31984	4369.7	rnafold:yes	ID=ID441	Mature:ID441-3P:scaffold47839:31893..31914:-	GGTGGATGTGGTGCACGGCGTC	-
scaffold4796	miRDeep2	200839	200947	167027.8	rnafold:yes	ID=ID802	Mature:ID802-5P:scaffold4796:200859..200881:+	TGTGATGTGCATGTGGGCTTCCC	-
scaffold4860	miRDeep2	656452	656563	20573.4	rnafold:yes	ID=ID1586	Mature:ID1586-3P:scaffold4860:656472..656493:-	TGGACGGAGAACTGATAAGGGC	aga-miR-184
scaffold4874	miRDeep2	1E+06	1E+06	1111.1	rnafold:yes	ID=ID732	Mature:ID732-3P:scaffold4874:1324840..1324861:+	AGGACTGTGTGTGGACATCAAC	dme-miR-219
scaffold4886	miRDeep2	509104	509215	336.5	rnafold:yes	ID=ID843	Mature:ID843-3P:scaffold4886:509174..509196:+	TGACTATCACTATTCTGTCTAGC	-
scaffold4975	miRDeep2	633819	633930	128	rnafold:yes	ID=ID59	Mature:ID59-3P:scaffold4975:633889..633910:+	TCCAAGGTGGAGAAATCGGTTC	-
scaffold4975	miRDeep2	647822	647933	96.9	rnafold:yes	ID=ID60	Mature:ID60-3P:scaffold4975:647892..647913:+	TTCCGACTGTAGTAGTTCCTGG	-
scaffold4975	miRDeep2	656232	656343	49353.3	rnafold:yes	ID=ID61	Mature:ID61-3P:scaffold4975:656308..656330:+	TACGGCCTGAAGATGTTCCTAGG	-
scaffold4975	miRDeep2	634062	634173	96.9	rnafold:yes	ID=ID64	Mature:ID64-3P:scaffold4975:634132..634153:+	TTCCGACTGTAGTAGTTCCTGG	-
scaffold4975	miRDeep2	663143	663250	468.8	rnafold:yes	ID=ID65	Mature:ID65-3P:scaffold4975:663213..663234:+	TCCTAACTGTAGTTGCTGCTGG	-
scaffold4975	miRDeep2	646625	646736	865.2	rnafold:yes	ID=ID66	Mature:ID66-5P:scaffold4975:646645..646666:+	AGGAACACCTTCAGACTGTGCA	-
scaffold4975	miRDeep2	639208	639319	96.8	rnafold:yes	ID=ID67	Mature:ID67-3P:scaffold4975:639278..639299:+	TTCCGACTGTAGTAGTTCCTGG	-
scaffold4975	miRDeep2	638965	639076	127.9	rnafold:yes	ID=ID68	Mature:ID68-3P:scaffold4975:639035..639056:+	TCCAAGGTGGAGAAATCGGTTC	-
scaffold5009	miRDeep2	572099	572210	3671.3	rnafold:yes	ID=ID395	Mature:ID395-5P:scaffold5009:572119..572140:+	TTTAGAATTCCTACGCTTTACC	aae-miR-927
scaffold509	miRDeep2	1E+06	1E+06	1081.4	rnafold:yes	ID=ID1580	Mature:ID1580-3P:scaffold509:1355654..1355673:+	CTGTAATCCAGCTACTTGGA	-
scaffold5119	miRDeep2	34108	34218	18309.6	rnafold:yes	ID=ID41	Mature:ID41-3P:scaffold5119:34128..34150:-	TCGCACGCACTGTGCGAGCGGAA	-
scaffold51459	miRDeep2	73061	73173	209	rnafold:yes	ID=ID684	Mature:ID684-5P:scaffold51459:73081..73103:+	CTCGCGCTTGAGCCATATACGTT	-
scaffold518	miRDeep2	397555	397664	304.3	rnafold:yes	ID=ID745	Mature:ID745-5P:scaffold518:397575..397597:+	AAGTGACTAATTTTCTGTAATAA	-

scaffold5198	miRDeep2	684479	684590	16.1	rnafold:yes	ID=ID374	Mature:ID374-5P:scaffold5198:684499..684520:+	ACCTTCTGGTGGTCGACCAGGA	-
scaffold52329	miRDeep2	132758	132870	2426.3	rnafold:yes	ID=ID876	Mature:ID876-3P:scaffold52329:132778..132800:-	TTGAGCATCATACGTCTGTGTGA	-
scaffold5239	miRDeep2	2E+06	2E+06	2120.9	rnafold:yes	ID=ID461	Mature:ID461-3P:scaffold5239:1501847..1501868:+	CAGGTCGGTGTAAATTGTGTGA	-
scaffold5286	miRDeep2	81801	81912	13625.6	rnafold:yes	ID=ID1650	Mature:ID1650-3P:scaffold5286:81871..81893:+	CACGTGACTCACCTACCGGATAG	-
scaffold5286	miRDeep2	111580	111689	1662	rnafold:yes	ID=ID1651	Mature:ID1651-5P:scaffold5286:111600..111621:+	TCAGGTACCAAGAATGTGATTC	-
scaffold5286	miRDeep2	266252	266363	120.1	rnafold:yes	ID=ID1652	Mature:ID1652-3P:scaffold5286:266322..266343:+	AATCTCTCTCCTGACACTGTCT	-
scaffold5286	miRDeep2	183781	183891	481.7	rnafold:yes	ID=ID1653	Mature:ID1653-3P:scaffold5286:183851..183872:+	AAGCACATTTCTGACACTGTCA	-
scaffold5286	miRDeep2	90917	91028	3034.2	rnafold:yes	ID=ID1654	Mature:ID1654-5P:scaffold5286:90937..90958:+	TGAAGCTCCTCATATCTGACCT	-
scaffold5286	miRDeep2	317613	317722	5858.4	rnafold:yes	ID=ID1655	Mature:ID1655-3P:scaffold5286:317683..317705:+	TCCTGTTGCACCTGTTGGTTGAC	-
scaffold5286	miRDeep2	347567	347674	1127.3	rnafold:yes	ID=ID1656	Mature:ID1656-3P:scaffold5286:347637..347657:+	TTCTGGCTCAATGCTCAGTCG	-
scaffold5286	miRDeep2	89769	89877	5536.2	rnafold:yes	ID=ID1657	Mature:ID1657-3P:scaffold5286:89839..89861:+	GAACTGACTCAACTACCGGACAG	-
scaffold5286	miRDeep2	65857	65965	1743.3	rnafold:yes	ID=ID1659	Mature:ID1659-5P:scaffold5286:65877..65899:+	GAAGCTCATCAGATGTGACCCGC	-
scaffold5286	miRDeep2	35996	36103	8591	rnafold:yes	ID=ID1660	Mature:ID1660-5P:scaffold5286:36016..36038:+	TGATGTGAAGCTCGTGCTGCTGA	-
scaffold5286	miRDeep2	319553	319661	3099.5	rnafold:yes	ID=ID1661	Mature:ID1661-5P:scaffold5286:319573..319595:+	TGATGCTGCAGGAGTTGTTGTGA	-
scaffold5286	miRDeep2	338111	338220	5858.4	rnafold:yes	ID=ID1662	Mature:ID1662-3P:scaffold5286:338181..338203:+	TCCTGTTGCACCTGTTGGTTGAC	-
scaffold5286	miRDeep2	114914	115021	89784.4	rnafold:yes	ID=ID1663	Mature:ID1663-5P:scaffold5286:114934..114956:+	TTAGCAGAAGGTAAGTGACTCGT	-
scaffold5286	miRDeep2	336404	336515	53.4	rnafold:yes	ID=ID1664	Mature:ID1664-3P:scaffold5286:336474..336495:+	CCCTGGTCCAACACCCAGTCGA	-
scaffold5286	miRDeep2	77607	77717	264.5	rnafold:yes	ID=ID1665	Mature:ID1665-3P:scaffold5286:77677..77698:+	AGGACACATCTGATAAGCTTCG	-
scaffold5286	miRDeep2	105991	106100	554.1	rnafold:yes	ID=ID1666	Mature:ID1666-5P:scaffold5286:106011..106032:+	ATAGTAACGGCGATGCGTCTCG	-
scaffold5286	miRDeep2	356015	356127	450.2	rnafold:yes	ID=ID1667	Mature:ID1667-5P:scaffold5286:356035..356056:+	TCCTGAGCCACGGTACAGGACG	-
scaffold5286	miRDeep2	47451	47560	10089.7	rnafold:yes	ID=ID1668	Mature:ID1668-3P:scaffold5286:47521..47541:+	TGCGAGAGCTGTACATGACCC	-
scaffold5286	miRDeep2	139607	139714	3233.7	rnafold:yes	ID=ID1670	Mature:ID1670-5P:scaffold5286:139627..139647:+	TGAGGTATCCAGGATGAGATT	-
scaffold5286	miRDeep2	95608	95718	131.3	rnafold:yes	ID=ID1671	Mature:ID1671-3P:scaffold5286:95680..95701:+	CGACTGCAGACGCAGACTGCAG	-
scaffold5286	miRDeep2	98369	98479	146.8	rnafold:yes	ID=ID1672	Mature:ID1672-3P:scaffold5286:98439..98460:+	ACTGACTGCCCTATTTCTTTGC	-
scaffold5286	miRDeep2	250058	250169	107777.1	rnafold:yes	ID=ID1673	Mature:ID1673-5P:scaffold5286:250078..250100:+	ATCGGAACAAGATGGTCTAATGC	-

scaffold5286	miRDeep2	133023	133133	2970.9	rnafold:yes	ID=ID1674	Mature:ID1674-3P:scaffold5286:133093..133114:+	AATTGCAGCCCAGGTACTCTCA	-
scaffold5286	miRDeep2	172086	172195	35353.4	rnafold:yes	ID=ID1675	Mature:ID1675-5P:scaffold5286:172115..172135:+	TCTGTACCAGGAATATGAATC	-
scaffold5286	miRDeep2	124825	124935	418.6	rnafold:yes	ID=ID1676	Mature:ID1676-5P:scaffold5286:124845..124866:+	GTGAGTACGCGGGTTGTGATTC	-
scaffold5286	miRDeep2	340421	340531	909.2	rnafold:yes	ID=ID1677	Mature:ID1677-3P:scaffold5286:340491..340512:+	TCCTGGTCCACCACCTGGTTGA	-
scaffold5286	miRDeep2	274239	274346	1332.4	rnafold:yes	ID=ID1678	Mature:ID1678-3P:scaffold5286:274309..274329:+	TCTCTCACTCCTGACGCTGCC	-
scaffold5286	miRDeep2	314328	314439	23.7	rnafold:yes	ID=ID1679	Mature:ID1679-3P:scaffold5286:314398..314419:+	CCCTGGTCCAACACCCAGTCGA	-
scaffold5286	miRDeep2	67337	67446	4681.1	rnafold:yes	ID=ID1680	Mature:ID1680-5P:scaffold5286:67368..67388:+	GAAGAGATAGAGGAGTCAACT	-
scaffold5286	miRDeep2	2121	2229	4531.6	rnafold:yes	ID=ID1681	Mature:ID1681-3P:scaffold5286:2191..2212:+	CAGGTACAAATGCACCGACCGC	-
scaffold5286	miRDeep2	293401	293512	6062	rnafold:yes	ID=ID1682	Mature:ID1682-5P:scaffold5286:293421..293443:+	AGGAAGATACGACGGGTCGTTTG	-
scaffold5286	miRDeep2	162296	162406	344.5	rnafold:yes	ID=ID1684	Mature:ID1684-5P:scaffold5286:162316..162337:+	ATGGTGTCAGGAATATGAGTCG	-
scaffold5286	miRDeep2	314982	315091	49738.1	rnafold:yes	ID=ID1685	Mature:ID1685-5P:scaffold5286:315002..315026:+	AACCAGGCGTTGGTCTTCAGGGCA	-
scaffold5286	miRDeep2	115621	115729	1299.5	rnafold:yes	ID=ID1686	Mature:ID1686-5P:scaffold5286:115641..115663:+	TCAGGTACCCAGTTTGTGATTCG	-
scaffold5286	miRDeep2	341759	341869	21.9	rnafold:yes	ID=ID1687	Mature:ID1687-5P:scaffold5286:341779..341802:+	AACTGGGTTTTGGGCACCAGAACG	-
scaffold5286	miRDeep2	84403	84514	157.5	rnafold:yes	ID=ID1688	Mature:ID1688-3P:scaffold5286:84473..84494:+	GAAGTGACCCCCCTAACTCGTT	-
scaffold5286	miRDeep2	59480	59590	1084.9	rnafold:yes	ID=ID1689	Mature:ID1689-5P:scaffold5286:59500..59521:+	TGTGACTCAGCGAAACCACAGC	-
scaffold5286	miRDeep2	314012	314121	258097.3	rnafold:yes	ID=ID1690	Mature:ID1690-5P:scaffold5286:314032..314055:+	AACCAGGCGTTGGTCTTCAGGACA	-
scaffold5286	miRDeep2	135110	135219	81.8	rnafold:yes	ID=ID1691	Mature:ID1691-3P:scaffold5286:135180..135201:+	AATTACAGCCTGAGTACTCTCA	-
scaffold5286	miRDeep2	72050	72160	4981.6	rnafold:yes	ID=ID1692	Mature:ID1692-3P:scaffold5286:72120..72141:+	AAGAACTCTGCCATTTCTTTGC	-
scaffold5286	miRDeep2	119846	119954	6358.6	rnafold:yes	ID=ID1693	Mature:ID1693-3P:scaffold5286:119916..119936:+	ATCACAGCCTCCGTACTCACA	-
scaffold5286	miRDeep2	128905	129016	1684.1	rnafold:yes	ID=ID1694	Mature:ID1694-5P:scaffold5286:128925..128946:+	AGAGTGCCAGGCGAGCAATTCT	-
scaffold5286	miRDeep2	334121	334230	4551.8	rnafold:yes	ID=ID1695	Mature:ID1695-3P:scaffold5286:334191..334213:+	TCCTGTTGCACCTGTTGGTTGAC	-
scaffold5286	miRDeep2	279591	279701	87.9	rnafold:yes	ID=ID1696	Mature:ID1696-3P:scaffold5286:279661..279682:+	ATTTTCGCTCCTGACACTTACC	-
scaffold5286	miRDeep2	288738	288847	31	rnafold:yes	ID=ID1697	Mature:ID1697-5P:scaffold5286:288758..288778:+	AGAGTGTCAAGAGCGCGACTC	-
scaffold5286	miRDeep2	119060	119171	34.3	rnafold:yes	ID=ID1698	Mature:ID1698-3P:scaffold5286:119130..119151:+	AATCACTTTCCTTCTGCTGACT	-
scaffold5286	miRDeep2	322965	323075	113.4	rnafold:yes	ID=ID1699	Mature:ID1699-5P:scaffold5286:322985..323006:+	AGGTTGTCATGGGACGACTGCC	-

scaffold5286	miRDeep2	143519	143630	3249.7	rnafold:yes	ID=ID1700	Mature:ID1700-5P:scaffold5286:143539..143560:+	CGCGTACCTGGGAACTGGGCCG	-
scaffold5286	miRDeep2	59306	59413	20543.4	rnafold:yes	ID=ID1701	Mature:ID1701-5P:scaffold5286:59326..59344:+	TGACTGGGTGATGCAGCAG	-
scaffold5286	miRDeep2	35663	35770	1004.2	rnafold:yes	ID=ID1702	Mature:ID1702-5P:scaffold5286:35683..35704:+	AGGCTCCAGTGATGCGACGGTG	-
scaffold5286	miRDeep2	207856	207965	161.4	rnafold:yes	ID=ID1703	Mature:ID1703-5P:scaffold5286:207876..207897:+	AGAGTGTCAGGAATGTGACTCG	-
scaffold5286	miRDeep2	341323	341433	17.6	rnafold:yes	ID=ID1704	Mature:ID1704-3P:scaffold5286:341383..341404:+	CCCTGGACCAACACCCAGTCGA	-
scaffold5286	miRDeep2	86686	86794	5536.2	rnafold:yes	ID=ID1705	Mature:ID1705-3P:scaffold5286:86756..86778:+	GAACTGACTCAACTACCGGACAG	-
scaffold5286	miRDeep2	93566	93676	5635.7	rnafold:yes	ID=ID1706	Mature:ID1706-5P:scaffold5286:93586..93606:+	GAAGAGATAGGACAGTCAATC	-
scaffold5286	miRDeep2	335491	335602	35	rnafold:yes	ID=ID1707	Mature:ID1707-3P:scaffold5286:335561..335582:+	TTCTCTTCCACCATCTGGTTGA	-
scaffold5286	miRDeep2	99275	99382	1227.4	rnafold:yes	ID=ID1708	Mature:ID1708-5P:scaffold5286:99295..99316:+	TAAGTCTCATCGGATATGACCA	-
scaffold5286	miRDeep2	339040	339150	19.6	rnafold:yes	ID=ID1709	Mature:ID1709-5P:scaffold5286:339060..339083:+	AACTGGGTTTTGGGCACCAGAACG	-
scaffold5286	miRDeep2	87486	87597	157.5	rnafold:yes	ID=ID1710	Mature:ID1710-3P:scaffold5286:87556..87577:+	GAAGTGACCCCCCTAACTCGTT	-
scaffold5286	miRDeep2	154459	154566	458345.8	rnafold:yes	ID=ID1711	Mature:ID1711-5P:scaffold5286:154479..154499:+	AAGTAGAAGGAACGTGAATCG	-
scaffold5286	miRDeep2	108459	108567	2170.1	rnafold:yes	ID=ID1712	Mature:ID1712-5P:scaffold5286:108479..108500:+	TTATGTAGTAGGAAAGTGAATC	-
scaffold5286	miRDeep2	334560	334670	19.7	rnafold:yes	ID=ID1713	Mature:ID1713-5P:scaffold5286:334580..334603:+	AACTGGGTTTTGGGCACCAGAACG	-
scaffold5286	miRDeep2	165217	165328	383.2	rnafold:yes	ID=ID1714	Mature:ID1714-5P:scaffold5286:165237..165258:+	ATGGTGTCAGGAATATGAGTCG	-
scaffold53358	miRDeep2	84852	84962	30.6	rnafold:yes	ID=ID643	Mature:ID643-5P:scaffold53358:84872..84892:+	GAAGGTCGCGGGTTCGTTCCC	-
scaffold5346	miRDeep2	129985	130092	4680.6	rnafold:yes	ID=ID1141	Mature:ID1141-5P:scaffold5346:130005..130026:+	TTAAGTAGTAGCGCCAGAGTGG	-
scaffold54263	miRDeep2	26550	26660	44.7	rnafold:yes	ID=ID1516	Mature:ID1516-5P:scaffold54263:26623..26644:-	AGCCACTACTACAGATGGAACA	-
scaffold54263	miRDeep2	45099	45210	99.4	rnafold:yes	ID=ID1517	Mature:ID1517-3P:scaffold54263:45119..45140:-	TTCCGACTGTAGTAGTTCCTGG	-
scaffold54263	miRDeep2	38491	38603	528.4	rnafold:yes	ID=ID1520	Mature:ID1520-3P:scaffold54263:38511..38533:-	ATGCTTCTGTTTCTCCTGCTGCT	-
scaffold54263	miRDeep2	38320	38431	580.2	rnafold:yes	ID=ID1523	Mature:ID1523-3P:scaffold54263:38340..38361:-	TCCTAACTGTAGTTGCTGCTGG	-
scaffold54263	miRDeep2	41142	41253	579	rnafold:yes	ID=ID1524	Mature:ID1524-3P:scaffold54263:41162..41183:-	TCCTAACTGTAGTTGCTGCTGG	-
scaffold5452	miRDeep2	1E+06	1E+06	76.2	rnafold:yes	ID=ID583	Mature:ID583-3P:scaffold5452:1107225..1107245:-	CAGGTGACTTGAAAATGGCGA	-
scaffold5456	miRDeep2	310828	310940	214.1	rnafold:yes	ID=ID22	Mature:ID22-5P:scaffold5456:310898..310920:-	ATAGAGCAGAGTTGACTTCATTG	-
scaffold55635	miRDeep2	21532	21643	471.7	rnafold:yes	ID=ID113	Mature:ID113-3P:scaffold55635:21602..21623:+	TCCCTGAGACCCATACGTTACT	-

scaffold55635	miRDeep2	32591	32702	93.2	rnafold:yes	ID=ID114	Mature:ID114-3P:scaffold55635:32661..32682:+	TACTTGAGGCACATTCGTTACT	-
scaffold55635	miRDeep2	66863	66973	707.7	rnafold:yes	ID=ID115	Mature:ID115-3P:scaffold55635:66933..66954:+	TTACTCTGGTACATACGTTACT	-
scaffold55635	miRDeep2	44581	44690	262.4	rnafold:yes	ID=ID116	Mature:ID116-3P:scaffold55635:44651..44672:+	TTCCTGTGGTACATACGTTACT	-
scaffold55635	miRDeep2	52796	52906	401	rnafold:yes	ID=ID117	Mature:ID117-3P:scaffold55635:52866..52887:+	TGCCACAACCACATACGTCACT	-
scaffold55635	miRDeep2	27594	27703	273.5	rnafold:yes	ID=ID118	Mature:ID118-5P:scaffold55635:27614..27636:+	TGACGTATGTGGTGGGGGAACAC	-
scaffold55635	miRDeep2	15481	15592	194.7	rnafold:yes	ID=ID119	Mature:ID119-3P:scaffold55635:15551..15572:+	TATCTGAGGCCCATTCGTTACT	-
scaffold55840	miRDeep2	36869	36980	27	rnafold:yes	ID=ID1323	Mature:ID1323-3P:scaffold55840:36939..36960:+	TGACTTTCGCTACTCCATCTAG	-
scaffold5645	miRDeep2	834878	834985	8053	rnafold:yes	ID=ID1743	Mature:ID1743-3P:scaffold5645:834948..834969:+	TTGGTCCCCTTCAACCAGCTGT	tur-miR-133
scaffold5645	miRDeep2	480919	481026	2271976	rnafold:yes	ID=ID1745	Mature:ID1745-3P:scaffold5645:480989..481010:+	TGGAATGTAAAGAAGTATGGAG	tca-miR-1
scaffold57669	miRDeep2	41360	41469	214	rnafold:yes	ID=ID1538	Mature:ID1538-5P:scaffold57669:41380..41401:+	TGAAGGTGGGAGTCTCTTGTGC	-
scaffold57669	miRDeep2	41206	41313	486007.2	rnafold:yes	ID=ID1539	Mature:ID1539-3P:scaffold57669:41276..41297:+	TCAGTCTTTTTCTCTCTCCTAT	dps-miR-14
scaffold5873	miRDeep2	157927	158036	35250	rnafold:yes	ID=ID162	Mature:ID162-5P:scaffold5873:157947..157968:+	ATATTGTCCTGTCACAGCAGTA	ame-miR-1000
scaffold58917	miRDeep2	27020	27128	5029.7	rnafold:yes	ID=ID3	Mature:ID3-5P:scaffold58917:27051..27073:+	CGTCTGAGTGCTGGACAGAGGGC	-
scaffold58917	miRDeep2	15970	16081	7590.3	rnafold:yes	ID=ID4	Mature:ID4-3P:scaffold58917:16040..16062:+	CTCTGTCCAGCACTCAGAGCGGG	-
scaffold58917	miRDeep2	23748	23858	949.1	rnafold:yes	ID=ID5	Mature:ID5-5P:scaffold58917:23768..23789:+	CATCTGAATGCTGGACAGAGGA	-
scaffold59215	miRDeep2	27327	27438	4061.2	rnafold:yes	ID=ID677	Mature:ID677-5P:scaffold59215:27410..27431:-	GGGATTGAACACCTGGCGGTTC	-
scaffold5931	miRDeep2	2682	2793	13708.3	rnafold:yes	ID=ID23	Mature:ID23-3P:scaffold5931:2702..2723:-	TAGCACCACATGATTCAGCTTA	dme-miR-995
scaffold597	miRDeep2	93774	93886	2418.9	rnafold:yes	ID=ID342	Mature:ID342-5P:scaffold597:93794..93816:+	AGACGAATGCATCTGTGGAGGCT	-
scaffold59941	miRDeep2	513	623	588.4	rnafold:yes	ID=ID1340	Mature:ID1340-3P:scaffold59941:533..554:-	TACCGCCTGAAGATGTTCCTAG	-
scaffold59941	miRDeep2	793	905	56649.3	rnafold:yes	ID=ID1341	Mature:ID1341-3P:scaffold59941:813..835:-	TACGGCCTGAAGATGTTCCTAGT	-
scaffold6070	miRDeep2	32914	33025	3652	rnafold:yes	ID=ID396	Mature:ID396-5P:scaffold6070:32934..32958:+	AACGATGGTACTGTACAGTATGTG	-
scaffold6078	miRDeep2	159895	160005	1604.4	rnafold:yes	ID=ID107	Mature:ID107-5P:scaffold6078:159915..159937:+	TAGTCTCAGGCTGGAGCAACGGT	-
scaffold6090	miRDeep2	56119	56228	5772.8	rnafold:yes	ID=ID360	Mature:ID360-5P:scaffold6090:56192..56211:-	AATTAACGTTTACTTCTGAT	-
scaffold61273	miRDeep2	59203	59314	262.5	rnafold:yes	ID=ID735	Mature:ID735-5P:scaffold61273:59223..59245:+	TCTGGCTCAGGATTGCCTGCCAT	-
scaffold6161	miRDeep2	81971	82082	37.8	rnafold:yes	ID=ID1634	Mature:ID1634-3P:scaffold6161:82041..82062:+	CTCTTTTGAATAGTCTGACCCA	-

scaffold61747	miRDeep2	32278	32385	10.7	rnafold:yes	ID=ID917	Mature:ID917-5P:scaffold61747:32348..32366:-	GGGGGCGTAGCTCATTGGC	-
scaffold6185	miRDeep2	40898	41009	70.4	rnafold:yes	ID=ID1272	Mature:ID1272-3P:scaffold6185:40919..40941:-	CCTCCGCATCACCAGCCTTCCGA	-
scaffold6199	miRDeep2	1E+06	1E+06	40.2	rnafold:yes	ID=ID856	Mature:ID856-3P:scaffold6199:1077130..1077151:+	TACCTTCACCAAATCTTCCTCA	-
scaffold62725	miRDeep2	9904	10015	1790.8	rnafold:yes	ID=ID926	Mature:ID926-5P:scaffold62725:9974..9995:-	TGCTGGATACTGAATACAGCGG	-
scaffold62725	miRDeep2	19750	19862	11828.7	rnafold:yes	ID=ID927	Mature:ID927-5P:scaffold62725:19820..19842:-	TGCTGGAGACTGAATACAGCGGT	-
scaffold62726	miRDeep2	719	830	1917.9	rnafold:yes	ID=ID928	Mature:ID928-5P:scaffold62726:789..811:-	CGAACTATCAATTCTATTGCTGA	-
scaffold6420	miRDeep2	2E+06	2E+06	8250.8	rnafold:yes	ID=ID893	Mature:ID893-3P:scaffold6420:1892345..1892366:+	TAGCACCATGGGATTCAGCTGA	api-miR-998
scaffold6420	miRDeep2	2E+06	2E+06	8090.4	rnafold:yes	ID=ID895	Mature:ID895-3P:scaffold6420:1882877..1882898:+	CATCACAGACAGAGTTCTAGTT	bmo-miR-11
scaffold646	miRDeep2	787652	787765	763.6	rnafold:yes	ID=ID1578	Mature:ID1578-5P:scaffold646:787722..787745:-	TGGACTTTGATCGGGATGGGCTAG	-
scaffold6545	miRDeep2	402422	402529	393.1	rnafold:yes	ID=ID1488	Mature:ID1488-5P:scaffold6545:402442..402465:+	TGAGATTGCACGGGAATTGGCAGC	-
scaffold6545	miRDeep2	406434	406545	201.8	rnafold:yes	ID=ID1489	Mature:ID1489-5P:scaffold6545:406454..406475:+	TGGACACGTGGCTGGCCTTGCA	-
scaffold6545	miRDeep2	406904	407015	308.8	rnafold:yes	ID=ID1490	Mature:ID1490-5P:scaffold6545:406924..406945:+	CAGGCGCGAGGTAGATCTAACA	-
scaffold6692	miRDeep2	340051	340162	35.1	rnafold:yes	ID=ID303	Mature:ID303-3P:scaffold6692:340121..340142:+	TGACTTAACAATTTCTGCACCT	-
scaffold6766	miRDeep2	18302	18412	983.2	rnafold:yes	ID=ID1179	Mature:ID1179-3P:scaffold6766:18372..18393:+	CCGCACGCACTGTGCGAGCGGA	-
scaffold6859	miRDeep2	65106	65216	91.8	rnafold:yes	ID=ID1093	Mature:ID1093-5P:scaffold6859:65144..65163:+	ACTCGCACAGTGCGTGCGGG	-
scaffold6931	miRDeep2	271588	271697	157.2	rnafold:yes	ID=ID130	Mature:ID130-5P:scaffold6931:271635..271657:+	AGCCCATATGGAGTCAGCTGGCA	-
scaffold694	miRDeep2	221246	221359	53.9	rnafold:yes	ID=ID8	Mature:ID8-3P:scaffold694:221266..221289:-	TATTACAAAATTTGAGAGTCCAT	-
scaffold7033	miRDeep2	2E+06	2E+06	819.5	rnafold:yes	ID=ID417	Mature:ID417-5P:scaffold7033:1848939..1848960:-	GGTACTCCGTCTTATGCTGACT	-
scaffold7055	miRDeep2	22509	22621	50.9	rnafold:yes	ID=ID210	Mature:ID210-3P:scaffold7055:22532..22553:-	TGAACCAGCAGCACACCCGTGG	-
scaffold70697	miRDeep2	6251	6363	45	rnafold:yes	ID=ID31	Mature:ID31-3P:scaffold70697:6321..6344:+	TGCTTGTAGAAGTTCTTAGACGGT	-
scaffold7070	miRDeep2	232564	232674	154593	rnafold:yes	ID=ID384	Mature:ID384-3P:scaffold7070:232584..232605:-	CAACATAGCGGCGAACACGGGT	-
scaffold728	miRDeep2	2E+06	2E+06	2987.8	rnafold:yes	ID=ID1298	Mature:ID1298-3P:scaffold728:1980602..1980623:-	AACCTTATTTTTGTAAATGGCA	-
scaffold7280	miRDeep2	503498	503605	149645.6	rnafold:yes	ID=ID1262	Mature:ID1262-3P:scaffold7280:503568..503589:+	TGGACGGAGATATGATAAGGTC	-
scaffold7301	miRDeep2	1E+06	1E+06	5079.7	rnafold:yes	ID=ID1367	Mature:ID1367-3P:scaffold7301:1352002..1352023:-	TTCGACGGCCTCAAGAATCTCC	-
scaffold7301	miRDeep2	1E+06	1E+06	1917.9	rnafold:yes	ID=ID1368	Mature:ID1368-5P:scaffold7301:1032200..1032222:-	CGAACTATCAATTCTATTGCTGA	-

scaffold7301	miRDeep2	1E+06	1E+06	1518.3	rnafold:yes	ID=ID1371	Mature:ID1371-5P:scaffold7301:1361390..1361413:-	TCCGTTCACCTGCCTTTCCGCGGG	-
scaffold7303	miRDeep2	419506	419617	29.3	rnafold:yes	ID=ID1300	Mature:ID1300-3P:scaffold7303:419526..419547:-	CGCGTGAACAGAACGGGAGGCC	-
scaffold732	miRDeep2	414928	415035	16785.6	rnafold:yes	ID=ID298	Mature:ID298-3P:scaffold732:414998..415018:+	TAAGGCACGCGGTGAATGCCA	aae-miR-124
scaffold7347	miRDeep2	169164	169273	20855.1	rnafold:yes	ID=ID1238	Mature:ID1238-5P:scaffold7347:169184..169205:+	TGGAACACGAGGTAGATTTGTC	-
scaffold7359	miRDeep2	1E+06	1E+06	13	rnafold:yes	ID=ID891	Mature:ID891-3P:scaffold7359:1372741..1372762:+	TTCTCCTGCTTTGTTCAGTTGT	-
scaffold7361	miRDeep2	229701	229811	4151.2	rnafold:yes	ID=ID1110	Mature:ID1110-3P:scaffold7361:229771..229793:+	CGTACACAGAAAGTATCACCATC	-
scaffold7361	miRDeep2	219321	219430	20920.7	rnafold:yes	ID=ID1111	Mature:ID1111-3P:scaffold7361:219391..219413:+	CGTACCTGTAAAGTAACACCATC	-
scaffold7361	miRDeep2	227145	227256	389.5	rnafold:yes	ID=ID1112	Mature:ID1112-3P:scaffold7361:227215..227238:+	CATACCTGTAAAGTATCACCATCT	-
scaffold7361	miRDeep2	223829	223938	20920.8	rnafold:yes	ID=ID1114	Mature:ID1114-3P:scaffold7361:223899..223921:+	CGTACCTGTAAAGTAACACCATC	-
scaffold7361	miRDeep2	220866	220977	389.5	rnafold:yes	ID=ID1115	Mature:ID1115-3P:scaffold7361:220936..220959:+	CATACCTGTAAAGTATCACCATCT	-
scaffold7361	miRDeep2	217670	217779	20920.7	rnafold:yes	ID=ID1117	Mature:ID1117-3P:scaffold7361:217740..217762:+	CGTACCTGTAAAGTAACACCATC	-
scaffold7361	miRDeep2	228820	228930	4151.2	rnafold:yes	ID=ID1118	Mature:ID1118-3P:scaffold7361:228890..228912:+	CGTACACAGAAAGTATCACCATC	-
scaffold7405	miRDeep2	696609	696722	60.7	rnafold:yes	ID=ID1362	Mature:ID1362-3P:scaffold7405:696629..696652:-	AAGGAGGTGAAATGTCGAGCGGTA	-
scaffold75019	miRDeep2	5944	6054	1121.2	rnafold:yes	ID=ID295	Mature:ID295-5P:scaffold75019:6014..6035:-	TAGCCTCTCCTAGGCTTTGTCT	dme-miR-282
scaffold75114	miRDeep2	34310	34420	622.5	rnafold:yes	ID=ID43	Mature:ID43-5P:scaffold75114:34331..34352:+	CTCTGAGTGCTGGACAGAGGAT	-
scaffold75114	miRDeep2	10880	10991	949.2	rnafold:yes	ID=ID44	Mature:ID44-5P:scaffold75114:10950..10971:-	CATCTGAATGCTGGACAGAGGA	-
scaffold75114	miRDeep2	8637	8748	4405.2	rnafold:yes	ID=ID45	Mature:ID45-5P:scaffold75114:8707..8729:-	CGTCTGAGTGCTGGACAGAGGGA	-
scaffold75114	miRDeep2	34306	34416	969.8	rnafold:yes	ID=ID46	Mature:ID46-3P:scaffold75114:34327..34349:-	CTCTGTCCAGCACTCAGAGCGGG	-
scaffold75114	miRDeep2	15281	15393	111.4	rnafold:yes	ID=ID47	Mature:ID47-5P:scaffold75114:15351..15374:-	CGATCTGAGTGCTGGACAGAGCAC	-
scaffold7540	miRDeep2	2E+06	2E+06	8350.9	rnafold:yes	ID=ID1582	Mature:ID1582-5P:scaffold7540:1575223..1575244:-	TTTTGATTGTTGCTCAGAAAGC	isc-miR-315
scaffold7554	miRDeep2	147677	147790	147565	rnafold:yes	ID=ID50	Mature:ID50-5P:scaffold7554:147747..147770:-	ATTGTACTTCATCAGGTGCTCTGG	dpu-miR-305
scaffold762	miRDeep2	249091	249202	260464.8	rnafold:yes	ID=ID1203	Mature:ID1203-5P:scaffold762:249161..249182:-	TCTCAGTTGTTGTAGGGACGGA	-
scaffold762	miRDeep2	249822	249933	7405.1	rnafold:yes	ID=ID1205	Mature:ID1205-3P:scaffold762:249843..249864:-	CTACAGCAACTGAAGCGGGTTG	-
scaffold762	miRDeep2	245264	245376	2406.2	rnafold:yes	ID=ID1206	Mature:ID1206-3P:scaffold762:245284..245307:-	TCTACAGCAACTGAACTGGGCTGG	-
scaffold762	miRDeep2	185314	185425	11963.4	rnafold:yes	ID=ID1207	Mature:ID1207-3P:scaffold762:185335..185356:-	CTACACCAACTGAAGCGGGTCG	-

scaffold762	miRDeep2	244180	244292	2380.1	rnafold:yes	ID=ID1208	Mature:ID1208-3P:scaffold762:244200..244223:-	TCTACAGCAACTGAACTGGGCTGG	-
scaffold762	miRDeep2	488365	488476	11965.9	rnafold:yes	ID=ID1210	Mature:ID1210-3P:scaffold762:488386..488407:-	CTACACCAACTGAAGCGGGTCG	-
scaffold762	miRDeep2	248354	248466	2406.2	rnafold:yes	ID=ID1211	Mature:ID1211-3P:scaffold762:248374..248397:-	TCTACAGCAACTGAACTGGGCTGG	-
scaffold762	miRDeep2	220401	220512	13159.2	rnafold:yes	ID=ID1212	Mature:ID1212-3P:scaffold762:220421..220443:-	CTATAGCAACTGAATCGGGCGGG	-
scaffold762	miRDeep2	209468	209579	7405.1	rnafold:yes	ID=ID1213	Mature:ID1213-3P:scaffold762:209489..209510:-	CTACAGCAACTGAAGCGGGTTG	-
scaffold762	miRDeep2	491668	491778	9009.8	rnafold:yes	ID=ID1214	Mature:ID1214-3P:scaffold762:491688..491708:-	TAAAGCAACTGAGGCGAGTGG	-
scaffold762	miRDeep2	210242	210352	34044.6	rnafold:yes	ID=ID1215	Mature:ID1215-3P:scaffold762:210262..210283:-	CTAGAGCAACTGAAGTGGACCG	-
scaffold762	miRDeep2	208735	208846	260003.8	rnafold:yes	ID=ID1218	Mature:ID1218-5P:scaffold762:208805..208826:-	TCTCAGTTGTTGTAGGGACGGA	-
scaffold765	miRDeep2	108380	108491	43.6	rnafold:yes	ID=ID1478	Mature:ID1478-3P:scaffold765:108400..108421:-	CATATATAGATCAACTGAAAGA	-
scaffold765	miRDeep2	1E+06	1E+06	302.5	rnafold:yes	ID=ID1479	Mature:ID1479-3P:scaffold765:1118710..1118732:-	TATTTGGCAGAGTGTATGGAAAT	-
scaffold766	miRDeep2	2E+06	2E+06	12555.6	rnafold:yes	ID=ID1440	Mature:ID1440-3P:scaffold766:1699035..1699056:-	TGGGAACCTTACACAGGTGTCT	-
scaffold76624	miRDeep2	13715	13826	5619.3	rnafold:yes	ID=ID1389	Mature:ID1389-3P:scaffold76624:13735..13756:-	TCCATTATACACTTCTGACGAC	-
scaffold7671	miRDeep2	10634	10743	485.8	rnafold:yes	ID=ID666	Mature:ID666-5P:scaffold7671:10704..10723:-	ACTCGCACAGTGCCTGCGGG	-
scaffold768	miRDeep2	96737	96847	326710.3	rnafold:yes	ID=ID1193	Mature:ID1193-5P:scaffold768:96757..96779:+	TCTTTGGTGATCTAGCTGTATGA	hme-miR-9b
scaffold768	miRDeep2	96502	96613	34689	rnafold:yes	ID=ID1194	Mature:ID1194-5P:scaffold768:96522..96543:+	CTTTGGTGATGTAGCTGTATGA	dme-miR-79
scaffold768	miRDeep2	94413	94523	1292200	rnafold:yes	ID=ID1195	Mature:ID1195-5P:scaffold768:94433..94454:+	TCTTTGGTATCCTAGCTGTAGG	-
scaffold768	miRDeep2	96143	96251	374225.9	rnafold:yes	ID=ID1196	Mature:ID1196-5P:scaffold768:96163..96184:+	TCAGGTACTGAGTGACTCTGAG	aae-miR-306
scaffold7800	miRDeep2	1E+06	1E+06	581.1	rnafold:yes	ID=ID1603	Mature:ID1603-3P:scaffold7800:1269625..1269646:-	CACCAGAGGAACTCACAGCTCC	-
scaffold7844	miRDeep2	135074	135184	560.5	rnafold:yes	ID=ID925	Mature:ID925-3P:scaffold7844:135144..135166:+	CCCGTCATGCACAAGTAGCTTCG	-
scaffold7876	miRDeep2	667042	667153	3051.3	rnafold:yes	ID=ID439	Mature:ID439-3P:scaffold7876:667063..667085:-	TATGCCCGAGGGAGGGCTCGAGG	-
scaffold78945	miRDeep2	64974	65083	20.2	rnafold:yes	ID=ID1534	Mature:ID1534-5P:scaffold78945:64994..65015:+	TCGGGTGAACTGCCGGCGATCC	-
scaffold796	miRDeep2	2E+06	2E+06	6253.2	rnafold:yes	ID=ID106	Mature:ID106-3P:scaffold796:2414372..2414393:+	TAGCACCATTTGAAATCAGTGC	isc-miR-285
scaffold7972	miRDeep2	504060	504174	148.4	rnafold:yes	ID=ID343	Mature:ID343-5P:scaffold7972:504083..504107:+	TGAGAGATATGGAGAATGTGCTGG	-
scaffold799	miRDeep2	2E+06	2E+06	22.6	rnafold:yes	ID=ID1352	Mature:ID1352-3P:scaffold799:1749643..1749663:+	TTTTATCACCCTCACATCACC	-
scaffold7995	miRDeep2	176305	176415	6869.1	rnafold:yes	ID=ID710	Mature:ID710-5P:scaffold7995:176335..176356:+	AGGATCAGCGGAAGTGGACGGT	-

scaffold7995	miRDeep2	188851	188961	106469.5	rnafold:yes	ID=ID711	Mature:ID711-3P:scaffold7995:188921..188943:+	TGTCTACTTCCACTGATCCCGCC	-
scaffold7995	miRDeep2	180563	180674	1047.9	rnafold:yes	ID=ID712	Mature:ID712-5P:scaffold7995:180593..180614:+	AGGATCAGCGGAAGTGGACGGT	-
scaffold7995	miRDeep2	186004	186114	106469.6	rnafold:yes	ID=ID714	Mature:ID714-3P:scaffold7995:186074..186096:+	TGTCTACTTCCACTGATCCCGCC	-
scaffold7995	miRDeep2	165093	165204	1047.8	rnafold:yes	ID=ID715	Mature:ID715-5P:scaffold7995:165123..165144:+	AGGATCAGCGGAAGTGGACGGT	-
scaffold7995	miRDeep2	183367	183478	1396.7	rnafold:yes	ID=ID716	Mature:ID716-5P:scaffold7995:183398..183418:+	GGATCAGCGGAAGTGGACGGT	-
scaffold7995	miRDeep2	258644	258752	125629.4	rnafold:yes	ID=ID717	Mature:ID717-3P:scaffold7995:258714..258736:+	TGTCTACCTCCACTGATCCCGCT	-
scaffold7995	miRDeep2	174546	174656	100530	rnafold:yes	ID=ID718	Mature:ID718-3P:scaffold7995:174616..174638:+	TGTCTACTTCCACTGATCCCGCC	-
scaffold8147	miRDeep2	253894	254006	199321.5	rnafold:yes	ID=ID1326	Mature:ID1326-5P:scaffold8147:253914..253935:+	TGCTTGATAGGACAGACACTGG	-
scaffold8147	miRDeep2	267711	267823	199333.6	rnafold:yes	ID=ID1327	Mature:ID1327-5P:scaffold8147:267731..267752:+	TGCTTGATAGGACAGACACTGG	-
scaffold8147	miRDeep2	267098	267210	199333.9	rnafold:yes	ID=ID1328	Mature:ID1328-5P:scaffold8147:267118..267139:+	TGCTTGATAGGACAGACACTGG	-
scaffold8147	miRDeep2	253781	253892	142.1	rnafold:yes	ID=ID1329	Mature:ID1329-5P:scaffold8147:253801..253822:+	TGCTTCATATGACAGACACAGG	-
scaffold8147	miRDeep2	268329	268441	199333.7	rnafold:yes	ID=ID1330	Mature:ID1330-5P:scaffold8147:268349..268370:+	TGCTTGATAGGACAGACACTGG	-
scaffold8147	miRDeep2	268626	268737	353.4	rnafold:yes	ID=ID1331	Mature:ID1331-5P:scaffold8147:268646..268667:+	TTCTTTATATGACAGACACTGG	-
scaffold8147	miRDeep2	268013	268125	199333.9	rnafold:yes	ID=ID1332	Mature:ID1332-5P:scaffold8147:268033..268054:+	TGCTTGATAGGACAGACACTGG	-
scaffold8147	miRDeep2	205527	205639	132301.8	rnafold:yes	ID=ID1333	Mature:ID1333-5P:scaffold8147:205547..205568:+	TGCTTGATAGGACAGACACTGG	-
scaffold8147	miRDeep2	267400	267509	20972.3	rnafold:yes	ID=ID1334	Mature:ID1334-5P:scaffold8147:267420..267441:+	TGCTTGATAGGACAGACACAGG	-
scaffold8150	miRDeep2	618827	618939	4197.3	rnafold:yes	ID=ID29	Mature:ID29-3P:scaffold8150:618897..618920:+	TGAGATTCAACTCCTCCAACTTAT	tca-miR-1175
scaffold8150	miRDeep2	618449	618557	77497.7	rnafold:yes	ID=ID30	Mature:ID30-3P:scaffold8150:618519..618541:+	CCAGATCTAACTCTTCCAGCTCA	ame-miR-750
scaffold8201	miRDeep2	373851	373958	2431.5	rnafold:yes	ID=ID1509	Mature:ID1509-5P:scaffold8201:373884..373905:+	TGATGGGCGTGCGATTTCACAT	-
scaffold8201	miRDeep2	368210	368320	3629.6	rnafold:yes	ID=ID1510	Mature:ID1510-5P:scaffold8201:368230..368252:+	CGGTGGAAACCTACACGAACACG	-
scaffold8201	miRDeep2	367710	367818	4669	rnafold:yes	ID=ID1511	Mature:ID1511-5P:scaffold8201:367730..367752:+	CGGAGGAAACCTACACGAACACG	-
scaffold8201	miRDeep2	374152	374262	252.1	rnafold:yes	ID=ID1513	Mature:ID1513-5P:scaffold8201:374172..374193:+	TCACAGAAGCGTTAGTTGCACA	-
scaffold82852	miRDeep2	68905	69016	47.3	rnafold:yes	ID=ID133	Mature:ID133-5P:scaffold82852:68925..68947:+	TTGGTATTTCGGGTTCCTGCCTG	-
scaffold83103	miRDeep2	782	893	6593.1	rnafold:yes	ID=ID1233	Mature:ID1233-3P:scaffold83103:802..824:-	CTCTGTCTATCATTTGGAGGACA	-
scaffold8343	miRDeep2	15411	15519	172262.7	rnafold:yes	ID=ID1318	Mature:ID1318-3P:scaffold8343:15481..15501:+	TGACTAGATCCATACTCGTCT	ame-miR-996

scaffold8349	miRDeep2	26217	26329	67.7	rnafold:yes	ID=ID1177	Mature:ID1177-5P:scaffold8349:26237..26259:+	CCACTGCAGAACCCTCAAACTCT	-
scaffold8428	miRDeep2	57999	58112	110.9	rnafold:yes	ID=ID1313	Mature:ID1313-5P:scaffold8428:58019..58040:+	AAGGATGTGACTGTAGCAGATG	-
scaffold854	miRDeep2	162553	162664	63.6	rnafold:yes	ID=ID646	Mature:ID646-3P:scaffold854:162623..162645:+	TTATCGGATCAACTCAATTTAAC	-
scaffold8803	miRDeep2	516775	516884	4434.6	rnafold:yes	ID=ID1001	Mature:ID1001-5P:scaffold8803:516795..516817:+	TGATATGCGTTTGATGGAAACAG	-
scaffold8803	miRDeep2	319515	319624	779.6	rnafold:yes	ID=ID1002	Mature:ID1002-5P:scaffold8803:319535..319556:+	TGATGGTTGTGTGGCATTAACA	-
scaffold8803	miRDeep2	499080	499187	5698.9	rnafold:yes	ID=ID1003	Mature:ID1003-5P:scaffold8803:499100..499122:+	TGATGTGCGTTTGATGGAACTAG	-
scaffold8803	miRDeep2	411976	412083	853.9	rnafold:yes	ID=ID1004	Mature:ID1004-3P:scaffold8803:412046..412067:+	TTGAACATACACGGCCATCACT	-
scaffold8803	miRDeep2	332944	333051	2950.8	rnafold:yes	ID=ID1005	Mature:ID1005-3P:scaffold8803:333014..333035:+	TTGAGGCCATGTAGTCATCACT	-
scaffold8803	miRDeep2	542853	542960	5175.8	rnafold:yes	ID=ID1006	Mature:ID1006-5P:scaffold8803:542873..542895:+	TGATGTGCGTTTGATGGAAACAG	-
scaffold8803	miRDeep2	475765	475876	1055.7	rnafold:yes	ID=ID1007	Mature:ID1007-5P:scaffold8803:475785..475806:+	TGGCGATTGCATGACTAATACA	-
scaffold8803	miRDeep2	746435	746546	117.2	rnafold:yes	ID=ID1009	Mature:ID1009-5P:scaffold8803:746455..746476:+	CGGCGACTGCATGAACACCACT	-
scaffold8803	miRDeep2	424520	424629	399.2	rnafold:yes	ID=ID1010	Mature:ID1010-5P:scaffold8803:424540..424560:+	TTGTGGCTGTGTATGACAACA	-
scaffold8803	miRDeep2	518172	518281	107.3	rnafold:yes	ID=ID1011	Mature:ID1011-5P:scaffold8803:518204..518224:+	TGATGTCTGTGTGGGTCCAAC	-
scaffold8803	miRDeep2	490688	490798	230.6	rnafold:yes	ID=ID1012	Mature:ID1012-3P:scaffold8803:490758..490780:+	TCACAGACGCCCAACCGTTACCT	-
scaffold8803	miRDeep2	402762	402871	274.4	rnafold:yes	ID=ID1013	Mature:ID1013-3P:scaffold8803:402832..402853:+	TTGCACTTCCACACACATCACT	-
scaffold8803	miRDeep2	213046	213157	711.1	rnafold:yes	ID=ID1014	Mature:ID1014-5P:scaffold8803:213080..213104:+	TGACGGAAGCGAGGTCTGTACAAG	-
scaffold8803	miRDeep2	432856	432967	344.1	rnafold:yes	ID=ID1015	Mature:ID1015-5P:scaffold8803:432889..432909:+	TGATGGCTGTGTCTACCAGCA	-
scaffold8803	miRDeep2	592320	592431	290.4	rnafold:yes	ID=ID1016	Mature:ID1016-5P:scaffold8803:592352..592372:+	TGATGTCTGTGTGGGTCCAAC	-
scaffold8803	miRDeep2	511296	511406	1535.4	rnafold:yes	ID=ID1017	Mature:ID1017-3P:scaffold8803:511366..511387:+	TTGAACACACACGGCTATCACT	-
scaffold8803	miRDeep2	392251	392362	118.7	rnafold:yes	ID=ID1019	Mature:ID1019-3P:scaffold8803:392321..392343:+	TCACAATCACCCAGTCGTCAGCT	-
scaffold8803	miRDeep2	705656	705767	27.9	rnafold:yes	ID=ID1020	Mature:ID1020-5P:scaffold8803:705692..705713:+	CGGCGATTGCATGACTACCACA	-
scaffold8803	miRDeep2	445194	445305	241.1	rnafold:yes	ID=ID1021	Mature:ID1021-5P:scaffold8803:445227..445247:+	TGATGGCTGTGTCTACCAGCA	-
scaffold8803	miRDeep2	645227	645338	72.7	rnafold:yes	ID=ID1022	Mature:ID1022-3P:scaffold8803:645297..645318:+	TTGTATAGGCCCAGCTATCATT	-
scaffold8803	miRDeep2	598031	598140	201	rnafold:yes	ID=ID1023	Mature:ID1023-3P:scaffold8803:598101..598122:+	TTGAGCATACACTGTCATCACT	-
scaffold8803	miRDeep2	470003	470113	661.2	rnafold:yes	ID=ID1024	Mature:ID1024-3P:scaffold8803:470073..470094:+	TTTCAGTCACCCAATCGTCAGC	-

scaffold8803	miRDeep2	621771	621880	1971.3	rnafold:yes	ID=ID1025	Mature:ID1025-3P:scaffold8803:621841..621862:+	TAACAGACACCCTGACGTCAGC	-
scaffold8803	miRDeep2	382314	382425	1378.8	rnafold:yes	ID=ID1026	Mature:ID1026-3P:scaffold8803:382384..382406:+	TCACAACCACCCAATCGTCAGCT	-
scaffold8803	miRDeep2	3224	3332	151.7	rnafold:yes	ID=ID1027	Mature:ID1027-3P:scaffold8803:3294..3315:+	TTCGCCAGCAACAGTAGCGGGC	-
scaffold8803	miRDeep2	544280	544387	2054.9	rnafold:yes	ID=ID1028	Mature:ID1028-3P:scaffold8803:544350..544371:+	TTGAACAGACACGGCCATCACT	-
scaffold8803	miRDeep2	699836	699947	256.1	rnafold:yes	ID=ID1029	Mature:ID1029-5P:scaffold8803:699869..699889:+	TGATAGCTGTGTCTACCAACA	-
scaffold8803	miRDeep2	19805	19917	835.9	rnafold:yes	ID=ID1030	Mature:ID1030-5P:scaffold8803:19839..19859:+	GGGGGCTGCTGCAGGCGAGCA	-
scaffold8803	miRDeep2	676924	677034	57.9	rnafold:yes	ID=ID1031	Mature:ID1031-5P:scaffold8803:676944..676965:+	TGGCGATTGTGTGACTGTTACG	-
scaffold8803	miRDeep2	742807	742918	53.7	rnafold:yes	ID=ID1032	Mature:ID1032-3P:scaffold8803:742877..742898:+	TGGCAGTGACGCAGTCGTCAGA	-
scaffold8803	miRDeep2	406346	406455	456.2	rnafold:yes	ID=ID1033	Mature:ID1033-5P:scaffold8803:406366..406386:+	TGATATCTGTGTGGGCCCAAC	-
scaffold8803	miRDeep2	349163	349270	16144.7	rnafold:yes	ID=ID1034	Mature:ID1034-5P:scaffold8803:349183..349204:+	TTATGGTAGAGTGGTGACAACA	-
scaffold8803	miRDeep2	241625	241734	353.7	rnafold:yes	ID=ID1035	Mature:ID1035-5P:scaffold8803:241645..241669:+	TAACGGAAGCCGAGGCTACACAAG	-
scaffold8803	miRDeep2	444531	444639	771.8	rnafold:yes	ID=ID1036	Mature:ID1036-3P:scaffold8803:444601..444622:+	TTGTTAAGACCCAGCCATCACT	-
scaffold8803	miRDeep2	328608	328715	2951	rnafold:yes	ID=ID1037	Mature:ID1037-3P:scaffold8803:328678..328699:+	TTGAGGCCATGTAGTCATCACT	-
scaffold8803	miRDeep2	767082	767189	490.1	rnafold:yes	ID=ID1038	Mature:ID1038-5P:scaffold8803:767102..767124:+	TGACGGATGTCTGACTGGAACAT	-
scaffold8803	miRDeep2	458280	458389	779.8	rnafold:yes	ID=ID1039	Mature:ID1039-3P:scaffold8803:458350..458371:+	TTTCAGTCATGCAATCGTCAGC	-
scaffold8803	miRDeep2	726689	726799	177.9	rnafold:yes	ID=ID1040	Mature:ID1040-3P:scaffold8803:726759..726780:+	TCACAGCCGCGCAATCGTCAGC	-
scaffold8803	miRDeep2	522701	522808	1962.4	rnafold:yes	ID=ID1041	Mature:ID1041-3P:scaffold8803:522771..522792:+	TTGAACAGACACGGCCATCACT	-
scaffold8803	miRDeep2	577062	577172	240.1	rnafold:yes	ID=ID1043	Mature:ID1043-5P:scaffold8803:577082..577103:+	CTCCGACTGCATGAACGCCACA	-
scaffold8803	miRDeep2	765593	765700	511.1	rnafold:yes	ID=ID1044	Mature:ID1044-5P:scaffold8803:765613..765635:+	TGACGGATGTCTGACTGGAACAT	-
scaffold8803	miRDeep2	450565	450674	399.2	rnafold:yes	ID=ID1046	Mature:ID1046-5P:scaffold8803:450585..450605:+	TTGTGGCTGTGTATGACAACA	-
scaffold8803	miRDeep2	214848	214957	1281.5	rnafold:yes	ID=ID1047	Mature:ID1047-3P:scaffold8803:214918..214939:+	TTGAGATCTCGATTCCGTTACT	-
scaffold8803	miRDeep2	349428	349539	11.6	rnafold:yes	ID=ID1048	Mature:ID1048-3P:scaffold8803:349498..349519:+	TGTGGCCTGCCCTACCATCACT	-
scaffold8803	miRDeep2	20968	21079	1803.8	rnafold:yes	ID=ID1049	Mature:ID1049-5P:scaffold8803:20988..21010:+	TGGGGGCTGCTGCAGACGAGCAT	-
scaffold8803	miRDeep2	437720	437831	365.9	rnafold:yes	ID=ID1050	Mature:ID1050-5P:scaffold8803:437753..437773:+	TGATGGCTATGTCTACCAACA	-
scaffold8803	miRDeep2	626459	626570	155.6	rnafold:yes	ID=ID1052	Mature:ID1052-5P:scaffold8803:626479..626500:+	TGGCGACCGCATGAACACCACT	-

scaffold8803	miRDeep2	228132	228241	929.1	rnafold:yes	ID=ID1053	Mature:ID1053-3P:scaffold8803:228202..228223:+	TTGAGATCTCGATTCCGTTACT	-
scaffold8803	miRDeep2	428035	428145	438.2	rnafold:yes	ID=ID1054	Mature:ID1054-5P:scaffold8803:428055..428075:+	TTGTGGCTGTGTATGACAACA	-
scaffold8803	miRDeep2	500466	500576	1535.1	rnafold:yes	ID=ID1055	Mature:ID1055-3P:scaffold8803:500536..500557:+	TTGAACACACACGGCTATCACT	-
scaffold8803	miRDeep2	755145	755256	113.9	rnafold:yes	ID=ID1057	Mature:ID1057-5P:scaffold8803:755165..755186:+	CGGCGACTGCATGAACACCACT	-
scaffold8803	miRDeep2	419247	419356	114.5	rnafold:yes	ID=ID1059	Mature:ID1059-3P:scaffold8803:419317..419338:+	TTGCATTTGCACACACATCACC	-
scaffold8803	miRDeep2	537815	537922	5201	rnafold:yes	ID=ID1060	Mature:ID1060-5P:scaffold8803:537835..537857:+	TGATGTGCGTTTGATGGAAACAG	-
scaffold8803	miRDeep2	634611	634720	24.3	rnafold:yes	ID=ID930	Mature:ID930-5P:scaffold8803:634631..634654:+	TTGACGAGTGCCTGACTGGAACAT	-
scaffold8803	miRDeep2	531848	531955	6143.7	rnafold:yes	ID=ID931	Mature:ID931-5P:scaffold8803:531868..531890:+	TGATGTGCGTTTGATGGAACTAG	-
scaffold8803	miRDeep2	435270	435379	5289.4	rnafold:yes	ID=ID932	Mature:ID932-5P:scaffold8803:435290..435310:+	TTGTGACTGTGTATGACAACA	-
scaffold8803	miRDeep2	510082	510192	22.6	rnafold:yes	ID=ID933	Mature:ID933-3P:scaffold8803:510152..510172:+	TTTTATCACCCTCACATCACC	-
scaffold8803	miRDeep2	585177	585288	15.3	rnafold:yes	ID=ID934	Mature:ID934-5P:scaffold8803:585210..585230:+	TGACGGATGTCTCACTGGAAA	-
scaffold8803	miRDeep2	407191	407302	40.1	rnafold:yes	ID=ID936	Mature:ID936-5P:scaffold8803:407211..407232:+	TGATGTCTGTGTGGGCCCAACA	-
scaffold8803	miRDeep2	349743	349850	16551.3	rnafold:yes	ID=ID937	Mature:ID937-5P:scaffold8803:349763..349784:+	TTATGGTAGAGTGGTGACAACA	-
scaffold8803	miRDeep2	534300	534407	1790.6	rnafold:yes	ID=ID938	Mature:ID938-3P:scaffold8803:534370..534391:+	TTGAACAGACACGGCCATCACT	-
scaffold8803	miRDeep2	503513	503623	22.6	rnafold:yes	ID=ID939	Mature:ID939-3P:scaffold8803:503583..503603:+	TTTTATCACCCTCACATCACC	-
scaffold8803	miRDeep2	605271	605381	176.4	rnafold:yes	ID=ID940	Mature:ID940-3P:scaffold8803:605341..605362:+	TTGTACAGACCCAGTTATCACT	-
scaffold8803	miRDeep2	112056	112165	693.1	rnafold:yes	ID=ID941	Mature:ID941-3P:scaffold8803:112126..112147:+	TGGATTGCTCCTTCTGACGCCT	-
scaffold8803	miRDeep2	658284	658393	628.5	rnafold:yes	ID=ID942	Mature:ID942-3P:scaffold8803:658354..658375:+	TTTCAGTCATGCAATCGTCACC	-
scaffold8803	miRDeep2	350570	350679	329.4	rnafold:yes	ID=ID943	Mature:ID943-5P:scaffold8803:350590..350613:+	TGATGGTAGAGTAGTGCCAACACA	-
scaffold8803	miRDeep2	243532	243639	5087	rnafold:yes	ID=ID944	Mature:ID944-5P:scaffold8803:243552..243574:+	AGCATGATCAGTGGGCACGAATT	-
scaffold8803	miRDeep2	466078	466188	1225	rnafold:yes	ID=ID945	Mature:ID945-3P:scaffold8803:466148..466170:+	TATCAGTCACCCAATCGTCATTT	-
scaffold8803	miRDeep2	528528	528635	1962.4	rnafold:yes	ID=ID946	Mature:ID946-3P:scaffold8803:528598..528619:+	TTGAACAGACACGGCCATCACT	-
scaffold8803	miRDeep2	586683	586792	514.3	rnafold:yes	ID=ID947	Mature:ID947-5P:scaffold8803:586703..586725:+	TGACGGATGTCTGACTGCAACAT	-
scaffold8803	miRDeep2	425710	425819	402.8	rnafold:yes	ID=ID948	Mature:ID948-5P:scaffold8803:425730..425750:+	TTGTGGCTGTGTATGACAACA	-
scaffold8803	miRDeep2	325283	325392	308.9	rnafold:yes	ID=ID949	Mature:ID949-5P:scaffold8803:325316..325337:+	TGACGACTGTGTGGCTGCTGCA	-

scaffold8803	miRDeep2	350294	350401	16144.5	rnafold:yes	ID=ID950	Mature:ID950-5P:scaffold8803:350314..350335:+	TTATGGTAGAGTGGTGACAACA	-
scaffold8803	miRDeep2	486301	486411	230.7	rnafold:yes	ID=ID951	Mature:ID951-3P:scaffold8803:486371..486393:+	TCACAGACGCCCAACCGTTACCT	-
scaffold8803	miRDeep2	416427	416536	118.8	rnafold:yes	ID=ID952	Mature:ID952-3P:scaffold8803:416497..416518:+	TTGCATTTGCACACACATCACC	-
scaffold8803	miRDeep2	25950	26059	1590.7	rnafold:yes	ID=ID954	Mature:ID954-5P:scaffold8803:25970..25992:+	TGGGGGCTGCTGCAGACGAGCAT	-
scaffold8803	miRDeep2	240224	240336	52.3	rnafold:yes	ID=ID956	Mature:ID956-3P:scaffold8803:240294..240316:+	TCAGGACCGTCCCTTCCGTTTCT	-
scaffold8803	miRDeep2	457602	457712	3844.5	rnafold:yes	ID=ID957	Mature:ID957-3P:scaffold8803:457672..457693:+	TATCACTCTCCTAATCGTCACC	-
scaffold8803	miRDeep2	413854	413961	161.9	rnafold:yes	ID=ID958	Mature:ID958-5P:scaffold8803:413886..413906:+	TGATGTCTGTGTGGGTCCAAC	-
scaffold8803	miRDeep2	218128	218239	82.9	rnafold:yes	ID=ID959	Mature:ID959-3P:scaffold8803:218198..218219:+	TTGAGTACTCCCTTCCGTCACT	-
scaffold8803	miRDeep2	354070	354180	329.3	rnafold:yes	ID=ID960	Mature:ID960-3P:scaffold8803:354140..354162:+	TCACATACGCCCAATCGTCAGCT	-
scaffold8803	miRDeep2	348572	348681	127.3	rnafold:yes	ID=ID961	Mature:ID961-5P:scaffold8803:348592..348613:+	TGATGGTAGAGTGGTGCCAACA	-
scaffold8803	miRDeep2	222925	223039	2225.4	rnafold:yes	ID=ID962	Mature:ID962-5P:scaffold8803:222945..222969:+	CGACGAAAGCGAGGTCTGTACAAG	-
scaffold8803	miRDeep2	553704	553814	207.3	rnafold:yes	ID=ID963	Mature:ID963-3P:scaffold8803:553774..553795:+	TTGTACAGACCCAGTTATCACT	-
scaffold8803	miRDeep2	231863	231970	13393.5	rnafold:yes	ID=ID964	Mature:ID964-5P:scaffold8803:231883..231902:+	AGCATGATCAGTGGGCACGA	-
scaffold8803	miRDeep2	513232	513342	1535.4	rnafold:yes	ID=ID965	Mature:ID965-3P:scaffold8803:513302..513323:+	TTGAACACACACGGCTATCACT	-
scaffold8803	miRDeep2	747741	747848	511.1	rnafold:yes	ID=ID966	Mature:ID966-5P:scaffold8803:747761..747783:+	TGACGGATGTCTGACTGGAACAT	-
scaffold8803	miRDeep2	443868	443976	771.8	rnafold:yes	ID=ID967	Mature:ID967-3P:scaffold8803:443938..443959:+	TTGTTAAGACCCAGCCATCACT	-
scaffold8803	miRDeep2	344286	344397	40	rnafold:yes	ID=ID968	Mature:ID968-5P:scaffold8803:344306..344327:+	TAATGACTGCGTGACATTGACA	-
scaffold8803	miRDeep2	350007	350118	11.6	rnafold:yes	ID=ID970	Mature:ID970-3P:scaffold8803:350077..350098:+	TGTGGCCTGCCCTACCATCACT	-
scaffold8803	miRDeep2	716660	716770	60.7	rnafold:yes	ID=ID971	Mature:ID971-3P:scaffold8803:716730..716751:+	TAACAGACACTCTGACGTCAGC	-
scaffold8803	miRDeep2	559814	559924	660.9	rnafold:yes	ID=ID973	Mature:ID973-3P:scaffold8803:559884..559905:+	TTTCAGTCACCCAATCGTCAGC	-
scaffold8803	miRDeep2	704750	704857	688.4	rnafold:yes	ID=ID975	Mature:ID975-3P:scaffold8803:704820..704841:+	TATCACCCTCCTAATCGTCAGC	-
scaffold8803	miRDeep2	214057	214166	908.3	rnafold:yes	ID=ID977	Mature:ID977-3P:scaffold8803:214127..214148:+	TTGAGATCTCGATTCCGTTACT	-
scaffold8803	miRDeep2	447621	447730	5315.9	rnafold:yes	ID=ID979	Mature:ID979-5P:scaffold8803:447641..447661:+	TTGTGACTGTGTATGACAACA	-
scaffold8803	miRDeep2	403115	403223	77.2	rnafold:yes	ID=ID980	Mature:ID980-3P:scaffold8803:403185..403206:+	TTGCACTTCCACACACATCACC	-
scaffold8803	miRDeep2	476765	476875	226.9	rnafold:yes	ID=ID983	Mature:ID983-3P:scaffold8803:476835..476856:+	TCACAGCCGCGCAATCGTCAGC	-

scaffold8803	miRDeep2	665512	665621	1971.3	rnafold:yes	ID=ID984	Mature:ID984-3P:scaffold8803:665582..665603:+	TAACAGACACCCTGACGTCAGC	-
scaffold8803	miRDeep2	15941	16048	258.7	rnafold:yes	ID=ID985	Mature:ID985-3P:scaffold8803:16011..16032:+	TTCGGTTGTACTAGATACCCGC	-
scaffold8803	miRDeep2	226174	226283	576.2	rnafold:yes	ID=ID986	Mature:ID986-3P:scaffold8803:226244..226265:+	TTGAGATCTCGCTTCCGTTACT	-
scaffold8803	miRDeep2	776727	776838	75.7	rnafold:yes	ID=ID987	Mature:ID987-5P:scaffold8803:776747..776768:+	CGGCGACTGCGTGGACGCCACA	-
scaffold8803	miRDeep2	211058	211169	37.6	rnafold:yes	ID=ID989	Mature:ID989-3P:scaffold8803:211128..211149:+	TTGGGATCTCCCTTCCGTTACT	-
scaffold8803	miRDeep2	718005	718115	373.5	rnafold:yes	ID=ID990	Mature:ID990-3P:scaffold8803:718075..718096:+	TATCAGTCACCCAATCGTCATT	-
scaffold8803	miRDeep2	567510	567621	56.7	rnafold:yes	ID=ID991	Mature:ID991-3P:scaffold8803:567580..567601:+	TCACAGACGTGCAATCGTCAGC	-
scaffold8803	miRDeep2	247134	247245	64	rnafold:yes	ID=ID993	Mature:ID993-3P:scaffold8803:247204..247225:+	TAAGGCTCTCGTTTCGGTTCCT	-
scaffold8803	miRDeep2	651174	651281	688.4	rnafold:yes	ID=ID994	Mature:ID994-3P:scaffold8803:651244..651265:+	TATCACCCTCCTAATCGTCAGC	-
scaffold8803	miRDeep2	234144	234251	3099	rnafold:yes	ID=ID995	Mature:ID995-5P:scaffold8803:234164..234185:+	AGCATGATCAGTGGCATGAATT	-
scaffold8803	miRDeep2	532845	532955	28.2	rnafold:yes	ID=ID996	Mature:ID996-3P:scaffold8803:532915..532935:+	TTTTATCACCCTCACATCACC	-
scaffold8803	miRDeep2	383958	384069	1378.8	rnafold:yes	ID=ID997	Mature:ID997-3P:scaffold8803:384028..384050:+	TCACAACCACCCAATCGTCAGCT	-
scaffold8803	miRDeep2	230317	230428	73.2	rnafold:yes	ID=ID998	Mature:ID998-3P:scaffold8803:230387..230408:+	TTGAGTACTCCCTTCCGTCACT	-
scaffold8803	miRDeep2	524323	524433	27.9	rnafold:yes	ID=ID999	Mature:ID999-3P:scaffold8803:524393..524413:+	TTTTATCACCCTCACATCACC	-
scaffold8889	miRDeep2	45609	45721	100.4	rnafold:yes	ID=ID1505	Mature:ID1505-3P:scaffold8889:45679..45701:+	CTGTACCTGGTCACCTTCTTCCT	-
scaffold889	miRDeep2	229581	229694	28.8	rnafold:yes	ID=ID1752	Mature:ID1752-3P:scaffold889:229651..229674:+	AAAGTCGAAAGCTGAAGAATCTGG	-
scaffold8892	miRDeep2	53816	53924	2156.9	rnafold:yes	ID=ID568	Mature:ID568-5P:scaffold8892:53836..53855:+	AAAGTTCTGGTAGAAGAGAG	-
scaffold8958	miRDeep2	169310	169421	4640.7	rnafold:yes	ID=ID772	Mature:ID772-3P:scaffold8958:169330..169351:-	TTGGTGTTCTACCTTACAGTGA	api-miR-971
scaffold9031	miRDeep2	170989	171099	16.2	rnafold:yes	ID=ID569	Mature:ID569-5P:scaffold9031:171062..171083:-	AATTGCTACGTAGGTAATCAGA	-
scaffold91855	miRDeep2	485005	485116	3352.2	rnafold:yes	ID=ID551	Mature:ID551-5P:scaffold91855:485075..485096:-	TGGGGTATCTCGGCAAGTGTCG	-
scaffold920	miRDeep2	333587	333699	22335	rnafold:yes	ID=ID1142	Mature:ID1142-3P:scaffold920:333607..333629:-	ACTATTCTGTTAGTGACACTGAT	-
scaffold9298	miRDeep2	700646	700756	17730.1	rnafold:yes	ID=ID720	Mature:ID720-3P:scaffold9298:700716..700738:+	TCGCACGCACTGTGCGAGCGGAA	-
scaffold9368	miRDeep2	203671	203783	12.7	rnafold:yes	ID=ID1643	Mature:ID1643-5P:scaffold9368:203691..203713:+	TACCCTGTGTTGTTTGAGAGAAA	-
scaffold943	miRDeep2	5E+06	5E+06	352.5	rnafold:yes	ID=ID1561	Mature:ID1561-5P:scaffold943:5316310..5316331:-	AGAGACAGATTTTGTTAAGTTA	-
scaffold943	miRDeep2	5E+06	5E+06	462.9	rnafold:yes	ID=ID1562	Mature:ID1562-5P:scaffold943:5319853..5319874:-	AGACACAGGAGTTGTTAAGTTA	-

scaffold943	miRDeep2	5E+06	5E+06	1046.7	rnafold:yes	ID=ID1563	Mature:ID1563-5P:scaffold943:5321452..5321474:-	CCAGAGACAGATTTTGTTAAGTT	-
scaffold943	miRDeep2	5E+06	5E+06	109.6	rnafold:yes	ID=ID1564	Mature:ID1564-5P:scaffold943:5311452..5311473:-	AGAAACAGAAGTTGTTAAGTTA	-
scaffold943	miRDeep2	5E+06	5E+06	462.9	rnafold:yes	ID=ID1565	Mature:ID1565-5P:scaffold943:5313013..5313034:-	AGACACAGGAGTTGTTAAGTTA	-
scaffold943	miRDeep2	5E+06	5E+06	417.4	rnafold:yes	ID=ID1566	Mature:ID1566-5P:scaffold943:5314648..5314669:-	AGACATAGGAGTTGTTAAGTTA	-
scaffold951	miRDeep2	3E+06	3E+06	156.9	rnafold:yes	ID=ID631	Mature:ID631-3P:scaffold951:2919271..2919292:-	TGCAGTACAGTCTACGGGGCGA	-
scaffold9589	miRDeep2	15384	15496	6247.3	rnafold:yes	ID=ID1630	Mature:ID1630-3P:scaffold9589:15404..15426:-	TAATTATGGTAAATGGAGGCTGT	-
scaffold9632	miRDeep2	130761	130869	1663.9	rnafold:yes	ID=ID443	Mature:ID443-5P:scaffold9632:130781..130803:+	TGGGAATCGAACTTGTGTCCTTT	-
scaffold9773	miRDeep2	1E+06	1E+06	83.8	rnafold:yes	ID=ID182	Mature:ID182-3P:scaffold9773:1213158..1213179:-	TTTGACACTCAGAGGCCTGACA	-
scaffold9791	miRDeep2	43005	43116	1121.5	rnafold:yes	ID=ID498	Mature:ID498-5P:scaffold9791:43075..43096:-	CCTTCATCTCCCTAGCAAGACT	-
scaffold9808	miRDeep2	13643	13755	689.2	rnafold:yes	ID=ID1188	Mature:ID1188-3P:scaffold9808:13663..13685:-	CAGGGAAGACATGTTCAGTGATG	-
scaffold9859	miRDeep2	180335	180446	3527.5	rnafold:yes	ID=ID272	Mature:ID272-3P:scaffold9859:180355..180376:-	CCTCTTGAGACAGGTTGTCTGC	-
scaffold98893	miRDeep2	28453	28564	958.4	rnafold:yes	ID=ID576	Mature:ID576-3P:scaffold98893:28523..28544:+	TGTAGCCATAGTGCTAAGCCGA	-
scaffold98990	miRDeep2	39320	39428	55493.7	rnafold:yes	ID=ID766	Mature:ID766-5P:scaffold98990:39340..39364:+	TTGGAAAGAGACGACTGCATGAAA	-
scaffold101355	MapMi	3529	3652	40.82632	rnafold:yes	ID=IM1	Mature:IM1-5P:scaffold101355:3599..3620:+	ACTCCTGCTGCAGCTGTTGGCG	dme-miR-9380-5p
scaffold103800	MapMi	36386	36510	35.28333	rnafold:yes	ID=IM2	Mature:IM2-3P:scaffold103800:36425..36445:-	GCGGACGAACTAAAAGAAGAG	bmo-miR-3254
scaffold10491	MapMi	228338	228462	46.85	rnafold:yes	ID=IM3	Mature:IM3-3P:scaffold10491:228372..228393:-	TAGGAACTTCATACCGTGCTCT	dme-miR-276b-3p
scaffold1055	MapMi	817828	817955	35.48793	rnafold:yes	ID=IM4	Mature:IM4-5P:scaffold1055:817898..817916:+	CCAGCAGTGTACGTTTGTG	bmo-miR-2723
scaffold11025	MapMi	149563	149673	64.88	rnafold:yes	ID=IM5	Mature:IM5-5P:scaffold11025:149631..149650:+	TGCATTTTCTTTCTGTAAAC	dme-miR-4967-5p
scaffold1227	MapMi	259697	259822	35.19737	rnafold:yes	ID=IM6	Mature:IM6-3P:scaffold1227:259732..259753:-	TGTTAACTGTCAGACTGTGACT	dme-miR-999-3p
scaffold12869	MapMi	261290	261419	46.34444	rnafold:yes	ID=IM7	Mature:IM7-5P:scaffold12869:261358..261379:+	GTAAGGATTTAAATTAAATGTT	dps-miR-2504
scaffold12910	MapMi	319919	320040	41.58529	rnafold:yes	ID=IM8	Mature:IM8-3P:scaffold12910:319952..319972:-	CCAGCTGCCAGCTGCGAGAGG	dme-miR-4957-3p
scaffold1330	MapMi	566856	566975	41.85614	rnafold:yes	ID=IM9	Mature:IM9-5P:scaffold1330:566923..566941:+	AGGAGGTGTGATCGGTTTG	tca-miR-3900-3p
scaffold16166	MapMi	96142	96269	37.00424	rnafold:yes	ID=IM10	Mature:IM10-5P:scaffold16166:96211..96229:+	CCAGCAGTAGACGTGTGTG	bmo-miR-2723
scaffold1883	MapMi	424983	425102	37.09706	rnafold:yes	ID=IM11	Mature:IM11-5P:scaffold1883:425049..425068:-	AGGGCGCGGTGGTGAGCATG	mja-miR-6494
scaffold22270	MapMi	87027	87135	57.46923	rnafold:yes	ID=IM12	Mature:IM12-3P:scaffold22270:87053..87073:+	ACCCTGTAGATCCGAATTTGT	dya-miR-10

scaffold24530	MapMi	276332	276460	46.65714	rnafold:yes	ID=IM13	Mature:IM13-5P:scaffold24530:276402..276421:+	CAAGGGAAAATATCTGTATG	bmo-miR-2818
scaffold24765	MapMi	194165	194267	37.43947	rnafold:yes	ID=IM14	Mature:IM14-3P:scaffold24765:194178..194197:-	TAAGTATAGAGAGGATGTGG	dme-miR-1016-5p
scaffold259	MapMi	2E+06	2E+06	37.47619	rnafold:yes	ID=IM15	Mature:IM15-5P:scaffold259:2294670..2294688:+	GAACATCTGAGGAGCTAAA	tca-miR-3818-3p
scaffold26061	MapMi	17425	17518	36.82273	rnafold:yes	ID=IM16	Mature:IM16-3P:scaffold26061:17432..17449:+	TAAAAGAGCCGCGGCGGA	dvi-miR-9544a-3p
scaffold266	MapMi	350959	351061	36.02727	rnafold:yes	ID=IM17	Mature:IM17-3P:scaffold266:350975..350993:+	ATCATAACTTGCTCACTGA	tca-miR-3809-5p
scaffold27788	MapMi	85372	85472	35.43333	rnafold:yes	ID=IM18	Mature:IM18-3P:scaffold27788:85395..85416:+	TCAGGTACTGAGTGACTCTGAG	ame-miR-306
scaffold281180	MapMi	8	106	45.70556	rnafold:yes	ID=IM19	Mature:IM19-5P:scaffold281180:77..95:-	TGGGTTATTGTGCGCCGGC	tca-miR-3879-5p
scaffold28161	MapMi	160978	161099	35.07456	rnafold:yes	ID=IM20	Mature:IM20-3P:scaffold28161:161014..161035:-	AGTTGTTGACGTGGCTTTAGCG	tca-miR-3843-5p
scaffold2945	MapMi	1E+06	1E+06	70.8	rnafold:yes	ID=IM21	Mature:IM21-5P:scaffold2945:1084136..1084156:-	CTGGGACCTTTGGCTTTCGGT	dme-miR-4960-3p
scaffold2962	MapMi	1E+06	1E+06	65.05	rnafold:yes	ID=IM22	Mature:IM22-5P:scaffold2962:1280273..1280294:-	TTACAAAATATTCAGTATTTTG	api-miR-3037
scaffold30052	MapMi	63166	63249	43.46111	rnafold:yes	ID=IM23	Mature:IM23-3P:scaffold30052:63177..63199:-	TGTCATGGAGTTGCTCTCTTTAT	ame-miR-281
scaffold30061	MapMi	130076	130183	39.4	rnafold:yes	ID=IM24	Mature:IM24-5P:scaffold30061:130142..130162:+	CTTTGGTGTTTTAACTTATGA	tca-miR-9d-5p
scaffold30163	MapMi	14604	14709	54.39	rnafold:yes	ID=IM25	Mature:IM25-5P:scaffold30163:14674..14694:+	GATCGGAAAGTTTTATATGTT	ame-miR-3737
scaffold31719	MapMi	43113	43245	53.50645	rnafold:yes	ID=IM26	Mature:IM26-5P:scaffold31719:43183..43207:-	ACTCACTCAACCTGGGTGTGATGT	tca-miR-307-5p
scaffold32557	MapMi	12597	12698	54.44286	rnafold:yes	ID=IM27	Mature:IM27-5P:scaffold32557:12652..12669:+	CGTTGATTTGGTGTCCTA	tca-miR-3851f-5p
scaffold342387	MapMi	1081	1189	43.27647	rnafold:yes	ID=IM28	Mature:IM28-5P:scaffold342387:1150..1170:+	ACGGTTCTGTTACAGTGTAGA	smr-miR-3930-5p
scaffold344723	MapMi	308	410	36.44583	rnafold:yes	ID=IM29	Mature:IM29-5P:scaffold344723:378..398:-	TGATGGATGTTGGTGTAGAGG	dps-miR-2527
scaffold357	MapMi	399530	399650	35.87558	rnafold:yes	ID=IM30	Mature:IM30-3P:scaffold357:399570..399591:+	GAAACGTTCACTCTGCAGTTCT	dme-miR-3642-3p
scaffold36102	MapMi	169718	169831	36.44151	rnafold:yes	ID=IM31	Mature:IM31-3P:scaffold36102:169741..169761:-	TATCTGTATGTGCAGTCTTGC	dme-miR-2281-3p
scaffold3855	MapMi	366133	366253	41.63293	rnafold:yes	ID=IM32	Mature:IM32-5P:scaffold3855:366200..366218:-	GGAAGGGGGAGCCGCGGTG	tca-miR-3905-5p
scaffold3862	MapMi	1E+06	1E+06	66.64362	rnafold:yes	ID=IM33	Mature:IM33-5P:scaffold3862:1217160..1217180:+	CCAGCTGCCAGCTGCGAGCGC	dme-miR-4957-3p
scaffold3866	MapMi	677776	677863	47.43333	rnafold:yes	ID=IM34	Mature:IM34-3P:scaffold3866:677779..677800:+	TTACTGGACATTGTAAGAACGG	api-miR-3015a
scaffold3953	MapMi	2E+06	2E+06	47.51667	rnafold:yes	ID=IM35	Mature:IM35-3P:scaffold3953:2161088..2161110:-	CTTGTGCGTGTGACAGCGGCTAT	cqu-miR-210-3p
scaffold4012	MapMi	760589	760711	46.69348	rnafold:yes	ID=IM36	Mature:IM36-3P:scaffold4012:760627..760649:-	TAATACTGTCAGGTAACGATGTC	aae-miR-8-3p
scaffold4012	MapMi	760628	760688	40.08043	rnafold:yes	ID=IM37	Mature:IM37-3P:scaffold4012:760666..760687:-	CATCTTACCGGGCAGCATTAGA	dme-miR-8-5p

scaffold42266	MapMi	23984	24105	35.31111	rnafold:yes	ID=IM38	Mature:IM38-5P:scaffold42266:24052..24073:+	TTTTGTGAAACTGGTGGCTGCT	dps-miR-2570-3p
scaffold42420	MapMi	153340	153459	44.12	rnafold:yes	ID=IM39	Mature:IM39-5P:scaffold42420:153403..153420:-	TGTAGCTGCTTTGGGGCG	pxy-miR-2756
scaffold4374	MapMi	158532	158658	40.55	rnafold:yes	ID=IM40	Mature:IM40-5P:scaffold4374:158601..158620:+	CACAGTTGCTGTTTCTCTTA	dps-miR-994-3p
scaffold4513	MapMi	403090	403212	48.58448	rnafold:yes	ID=IM41	Mature:IM41-5P:scaffold4513:403154..403173:+	GCAAGTCAATGACATTATTC	dps-miR-2562-5p
scaffold4777	MapMi	16377	16498	39.10455	rnafold:yes	ID=IM42	Mature:IM42-5P:scaffold4777:16446..16467:+	AATTGCACTTTTCCCGGCCTGC	dwi-miR-92b
scaffold4927	MapMi	76754	76854	42.06744	rnafold:yes	ID=IM43	Mature:IM43-5P:scaffold4927:76817..76835:-	GTTGACCGAAACTGAGGAG	mja-miR-6492
scaffold4979	MapMi	57453	57574	54.44091	rnafold:yes	ID=IM44	Mature:IM44-3P:scaffold4979:57486..57504:+	CCAGCAGTCAACGTCTGTG	bmo-miR-2723
scaffold499	MapMi	109311	109417	42.87391	rnafold:yes	ID=IM45	Mature:IM45-3P:scaffold499:109330..109348:+	TAGACGGAGAACTGCTAAG	pxy-miR-184
scaffold5091	MapMi	1E+06	1E+06	37.72903	rnafold:yes	ID=IM46	Mature:IM46-3P:scaffold5091:1065225..1065248:-	CCTGCAGAGACATCTGTCGGACTC	ame-miR-3782
scaffold50987	MapMi	30146	30267	53.01552	rnafold:yes	ID=IM47	Mature:IM47-3P:scaffold50987:30177..30199:+	TCTTTGGTTATCTAGCTGTATGA	dsi-miR-9a
scaffold50987	MapMi	30146	30267	43.01552	rnafold:yes	ID=IM48	Mature:IM48-5P:scaffold50987:30215..30237:+	ATAAAGCTAGGTTACCGAAGTTA	isc-miR-79
scaffold52445	MapMi	52564	52692	38.29032	rnafold:yes	ID=IM49	Mature:IM49-3P:scaffold52445:52603..52624:-	TTACTGGACATTGTAAAAACGG	api-miR-3015a
scaffold544	MapMi	702665	702789	36.2	rnafold:yes	ID=IM50	Mature:IM50-3P:scaffold544:702703..702721:-	AACTGCAAACGGGTAATGT	tca-miR-3896-3p
scaffold54464	MapMi	44099	44217	35.05	rnafold:yes	ID=IM51	Mature:IM51-5P:scaffold54464:44164..44181:-	TAAAAGAGCCGCGGCGGA	dvi-miR-9544a-3p
scaffold5605	MapMi	106090	106205	35.73	rnafold:yes	ID=IM52	Mature:IM52-3P:scaffold5605:106130..106151:-	TTACTGGACATTGTAAGAACGG	api-miR-3015a
scaffold56943	MapMi	36089	36206	35.52143	rnafold:yes	ID=IM53	Mature:IM53-3P:scaffold56943:36128..36146:-	CCAGCAGTTCACGTCTGTG	bmo-miR-2723
scaffold5703	MapMi	118233	118323	36.86512	rnafold:yes	ID=IM54	Mature:IM54-5P:scaffold5703:118302..118319:-	GAAAAGAGCCGCGGCGCA	dvi-miR-9544a-3p
scaffold5880	MapMi	25730	25818	37.95	rnafold:yes	ID=IM55	Mature:IM55-5P:scaffold5880:25800..25817:+	AGGCCGATCTTAGGCTAA	tca-miR-3850-5p
scaffold59	MapMi	1E+06	1E+06	39.04672	rnafold:yes	ID=IM56	Mature:IM56-5P:scaffold59:1485043..1485062:+	TCACTGGGTGGAGTTTGGCT	tca-miR-309b-3p
scaffold5907	MapMi	389116	389207	39.23659	rnafold:yes	ID=IM57	Mature:IM57-5P:scaffold5907:389186..389204:+	AGCGGCGAGAGCACAGGAT	pxy-miR-8503
scaffold6056	MapMi	43920	44043	36.15345	rnafold:yes	ID=IM58	Mature:IM58-5P:scaffold6056:43990..44011:-	ACCCGAGCGGTCTAAGGAAACT	bmo-miR-375-5p
scaffold611	MapMi	2E+06	2E+06	53.01552	rnafold:yes	ID=IM59	Mature:IM59-5P:scaffold611:1531437..1531459:-	TCTTTGGTTATCTAGCTGTATGA	ame-miR-9a
scaffold611	MapMi	2E+06	2E+06	43.01552	rnafold:yes	ID=IM60	Mature:IM60-3P:scaffold611:1531399..1531421:-	ATAAAGCTAGGTTACCGAAGTTA	isc-miR-79
scaffold694	MapMi	708561	708672	64.23868	rnafold:yes	ID=IM61	Mature:IM61-3P:scaffold694:708583..708602:-	CACAGTTGCTGTTGTTTTTA	dps-miR-994-3p
scaffold71059	MapMi	63811	63928	39.25946	rnafold:yes	ID=IM62	Mature:IM62-5P:scaffold71059:63878..63897:-	TCAGGTACCTGTAGTAGCGC	aae-miR-275-3p

scaffold713	MapMi	665380	665504	38.50454	rnafold:yes	ID=IM63	Mature:IM63-3P:scaffold713:665416..665436:-	TTGTGCGTGTGACCTCGGCCA	api-miR-210
scaffold72558	MapMi	9988	10115	36.2	rnafold:yes	ID=IM64	Mature:IM64-3P:scaffold72558:10025..10045:-	GGGTGGTGGTGGAAGGGGGGG	ame-miR-3751
scaffold7301	MapMi	2E+06	2E+06	61	rnafold:yes	ID=IM65	Mature:IM65-3P:scaffold7301:2190370..2190389:+	CATTTATATTTCATTCACGT	tca-miR-3901-3p
scaffold74693	MapMi	30495	30603	43.27647	rnafold:yes	ID=IM66	Mature:IM66-5P:scaffold74693:30564..30584:+	ACGGTTCTGTTACAGTGTAGA	smr-miR-3930-5p
scaffold749	MapMi	1E+06	1E+06	36.78404	rnafold:yes	ID=IM67	Mature:IM67-5P:scaffold749:1190702..1190720:+	TACTGAATTTTATGTGGAC	tca-miR-998-5p
scaffold75	MapMi	633041	633156	36.59898	rnafold:yes	ID=IM68	Mature:IM68-3P:scaffold75:633072..633089:-	AGGCAAGATCTTGAGCAG	tca-miR-3865-5p
scaffold75550	MapMi	454	574	36.4	rnafold:yes	ID=IM69	Mature:IM69-3P:scaffold75550:494..512:+	TGGGTTATAGTGTGCCGGC	tca-miR-3879-5p
scaffold78654	MapMi	33172	33287	50.69091	rnafold:yes	ID=IM70	Mature:IM70-3P:scaffold78654:33197..33217:-	CCAGCTGCCAGCTGCGAGCGG	dme-miR-4957-3p
scaffold90	MapMi	1E+06	1E+06	43.8	rnafold:yes	ID=IM71	Mature:IM71-5P:scaffold90:1440311..1440329:-	CCAATGTCCCACGCCTTGC	tca-miR-3845-5p
scaffold9318	MapMi	734763	734886	38.2579	rnafold:yes	ID=IM72	Mature:IM72-5P:scaffold9318:734829..734847:+	ACTGGCCTACTAGGTCACA	dvi-miR-193-3p
scaffold9720	MapMi	25467	25595	55.42627	rnafold:yes	ID=IM73	Mature:IM73-5P:scaffold9720:25535..25555:-	CTGGGACCTTTGCCTTACGGT	dme-miR-4960-3p

Table S2. Summary of the predicted miRNAs in the different annotation steps

Methods	Functional molecules	Number	Number of miRNAs
miRDeep			779
	tRNA	2	
	rRNA	12	
	snRNA	1	
	snoRNA	3	
	transposable element	1	
	miRNA	760	
MapMi			144
Total (Combined by the two methods)			883
